# Genome-Wide Analysis of Watermelon HSP20s and Their Expression Profiles and Subcellular Locations under Stresses

**DOI:** 10.3390/ijms20010012

**Published:** 2018-12-20

**Authors:** Yanjun He, Min Fan, Yuyan Sun, Lili Li

**Affiliations:** Zhejiang Academy of Agricultural Sciences, Institute of Vegetables, Hangzhou 310021, China; hyj1009@163.com (Y.H.); Syy1111@126.com (Y.S.); 19906734996@163.com (L.L.)

**Keywords:** small heat shock protein 20, *Citrullus lanatus*, phylogeny, evolution, expression profiles, subcellular location, *cucumber green mottle mosaic virus* (CGMMV)

## Abstract

Watermelon (*Citrullus lanatus* L.), which is an economically important cucurbit crop that is cultivated worldwide, is vulnerable to various adverse environmental conditions. Small heat shock protein 20s (HSP20s) are the most abundant plant HSPs and they play important roles in various biotic and abiotic stress responses. However, they have not been systematically investigated in watermelon. In this study, we identified 44 watermelon *HSP20* genes and analyzed their gene structures, conserved domains, phylogenetic relationships, chromosomal distributions, and expression profiles. All of the watermelon HSP20 proteins have a conserved the α-crystallin (ACD) domain. Half of the *ClHSP20s* arose through gene duplication events. Plant HSP20s were grouped into 18 subfamiles and a new subfamily, nucleo-cytoplasmic XIII (CXIII), was identified in this study. Numerous stress- and hormone-responsive *cis*-elements were detected in the putative promoter regions of the watermelon *HSP20* genes. Different from that in other species, half of the watermelon *HSP20s* were repressed by heat stress. Plant *HSP20s* displayed diverse responses to different virus infections and most of the *ClHSP20s* were generally repressed by *Cucumber green mottle mosaic virus* (CGMMV). Some *ClHSP20s* exhibited similar transcriptional responses to abscisic acid, melatonin, and CGMMV. Subcellular localization analyses of six selected HSP20- green fluorescence protein fusion proteins revealed diverse subcellular targeting. Some ClHSP20 proteins were affected by CGMMV, as reflected by changes in the size, number, and distribution of fluorescent granules. These systematic analyses provide a foundation for elucidating the physiological functions and biological roles of the watermelon HSP20 gene family.

## 1. Introduction

Heat shock proteins (HSPs), an important group of molecular chaperones, have been proven to participate in various plant stress responses and in regulating plant growth and development [1]. On the basis of their molecular weight, HSPs can be classified into six families, namely, HSP100, HSP90, HSP70, HSP60, HSP20 (or small heat shock protein), and ubiquitin. Of these groups, HSP20 is the largest family and the most well studied [2]. Numerous investigations have revealed the roles of HSP20 proteins in various abiotic stress responses. Most *HSP20s* are highly induced by heat stress and transgenic plants overexpressing *HSP20s* in *Arabidopsis*, rice, maize, wheat, and *Chenopodium* sp., by protecting proteins from irreversible denaturation, enhanced tolerance to heat stress [3,4,5,6,7]. Overexpression of *OsHSP17.0* and *OsHSP23.7* in rice decreases membrane damage, increases the abundance of protective molecules, and leads to improved seed vigor under osmotic and salt stress conditions [8]. Transgenic rice overexpressing *OsHSP20s* exhibited enhanced tolerance to ultraviolet-B radiation, salt, drought, and dehydration stresses [4,9]. Promoter analyses revealed that the expression of *TaHSP26* in wheat may be induced by heat, cold, salt, and drought stresses [6,10]. Meanwhile, the ectopic expression of *LimHSP16.45* from David Lily (*Lilium davidii*) in *Arabidopsis* increased the levels of superoxide dismutase and catalase activities and seed germination vigor under salt stress [11]. *CaHsp22.5* in *Capsicum annuum* and *AsHSP17* in *Salix suchowensis* were shown to regulate plant responses to salt stress via abscisic acid (ABA) signaling pathways [12,13].

Previous studies confirmed that HSP20s play significant roles in plant immunity [14,15]. In rice, some *HSP20* genes were induced under *Magnaporthe grisea* infection, while other genes were repressed. Transgenic *Arabidopsis* with reduced *HSP20* expression levels was more susceptible to pathogens [16]. Tomato HSP20, which interacts with the R protein I-2, was shown to improve resistance to *Fusarium oxysporum* [17]. Proteomic analyses indicated that the expression of tomato HSP17.6 protein was repressed by *Rhizopus nigricans* infection [18]. Tobacco *HSP17.6* was found to participate in the immune response to *Ralstonia solanacearum* infection [16]. Potato *StHSP17.8* could activate the *Phytophthora infestans* resistance gene *WRKY1* only in resistant potato genotypes [19]. Two barley powdery mildew effector candidates, CSEP0105 and CSEP0162, which contributed to the success of *Blumeria graminis f.* sp. *hordei* infection, were found to interact with barley Hsp16.9 and Hsp17.5 [20]. *GmHsp20s* in soybean showed different response patterns between resistant and susceptible soybean genotypes under *Meloidogyne javanica* infection [21]. Microarray analyses have indicated that *Arabidopsis HSP20s* show various response patterns to different viral infections [22]. In rice, HSP20 was significantly affected by *Rice stripe virus* infection, and it was found to play an important role in RSV replication and movement via interacting with the RSV protein (RdRp) and the movement protein (NSVc4) [23,24]. 

Watermelon (*Citrullus lanatus*) is an important cucurbit crop that is consumed worldwide, but various adverse environmental conditions affect its development and decrease its yield and quality. Plants HSP20 proteins play important roles in the responses to various biotic and abiotic stresses. However, watermelon *HSP20* genes have not been systematically investigated at the whole genome level in watermelon. In this study, all putative *HSP20* elements in watermelon were identified in an in silico analysis. The classification, conserved domains, gene structures, chromosomal distributions, phylogenetic relationships, synteny relationships, and gene duplication events of watermelon *HSP20* genes were predicted and analyzed in detail. Their subcellular localizations were also predicted and verified after the transient transformation of tobacco leaf epidermal cells with (or without) *Cucumber green mottle mosaic virus* (CGMMV) infection. The expression profiles of *ClHSP20* genes were analyzed using quantitative real-time PCR analysis (qRT-PCR) to determine their responses to various stresses and plant hormone treatments. Our comprehensive analyses have elucidated the potential roles of *ClHSP20s* in stress and hormone responses. Our findings provide a framework for future studies to better understand the functions of watermelon *HSP20* family genes in stress tolerance and hormone responses.

## 2. Results

### 2.1. Identification of HSP20 Genes in Watermelon and Cucumber

We conducted a genome-wide analysis of *HSP20* gene family members in watermelon. Protein Basic Local Alignment Search Tool (BLASTP) searches were performed in the Cucurbit Genomics Database to identify putative HSP20s in watermelon. The search queries were 273 HSP20 protein sequences from *Arabidopsis*, rice, soybean, tomato, pepper, wheat, barley, and switchgrass. A total of 53 putative hits were identified in the watermelon genome database. Meanwhile, 41 sequences were annotated as being watermelon *HSP20* genes after searches, with HMMER 3.0 using the global hidden Markov models (HMM) profile of the HSP20 characteristic domain. Redundant sequences by the above two methods were omitted to obtain unique putative *HSP20* genes, and the remaining hits were further filtered using Conserved Domains Database (CDD) and Simple Modular Architecture Research Tool (SMART) according to the presence of structural characteristics and the conserved ACD domain. Finally, 44 *HSP20* genes were identified in watermelon. The same methods were applied to identify 45 *HSP20s* in cucumber. *HSP20* genes have been intensively studied in some model plant species and important crops. Among the plants studied to date, *Arabidopsis* has the smallest number of *HSP20s* and switchgrass has the largest number. The numbers of *HSP20* genes in watermelon (44) and cucumber (45) were greater than those in all other plant species, except for soybean (51) and switchgrass (63) (Table 1).

**Figure 1:**
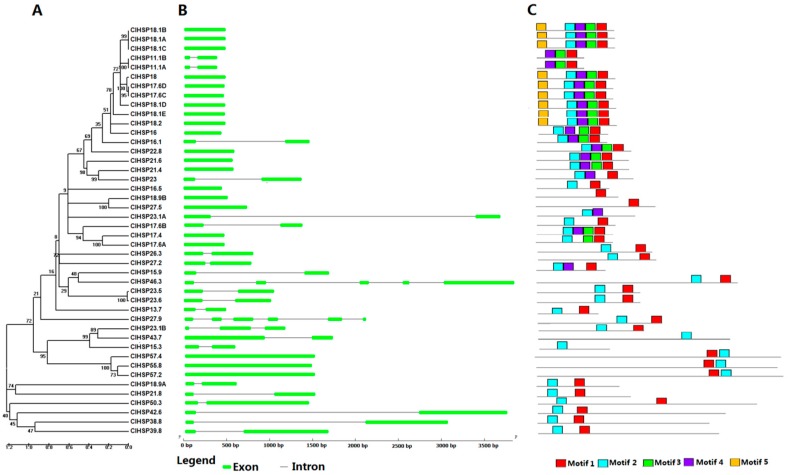
Phylogenetic relationships, gene structures, and conserved motifs of all *ClHSP20s* in watermelon. (**A**) Unrooted phylogenetic tree was generated based on the amino acid sequences by the neighbor-joining (NJ) method using MEGA 5. Bootstrap supports from 1000 replicates are indicated at each branch. (**B**) Gene structure was analyzed using the Gene Structure Display Server online. Green boxes indicate exons, lines indicate introns. (**C**) Motif analysis was performed using MEME 4.0 software. Different coloured boxes represent different motifs in corresponding position of each ClHSP20 protein.

**Figure 2:**
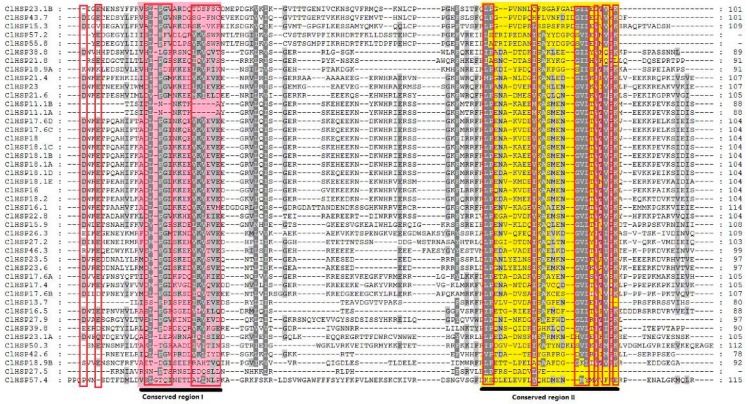
Amino acid sequence alignment of the α-crystallin (ACD) domain from watermelon HSP20s. Sequences were aligned by Clustal X program. Conserved motifs are marked. Conserved region I and II were shown for red and yellow background, respectively, and the typical amino acid residues within these regions were indicated by red boxes.

The amino acid sequences of all ClHSP20s ranged from 97 to 502 amino acids in length, corresponding to molecular weights ranging from 13.72 to 57.37 kDa (Table S1). The HSP20s in watermelon were named according to their protein molecular weight, as in previous studies [2,25,26]. Almost all of the ClHSP20s had a highly conserved α-crystallin domain (ACD) at the C-terminal end, as identified by SMART, as well as two conserved motifs (motif 1 and motif 2), as identified by MEME [30] (Figure 1 and Figure 2). Multiple sequence alignments indicated that the ACD domain contained two conserved regions with a β-sandwich of two antiparallel β-sheets domains (Figure S1 and Figure 2). These conserved regions are known to play important roles in the chaperone function of HSP20 proteins [31,32]. Seven ClHSP20 proteins (ClHSP18.9A, ClHSP21.8, ClHSP27.5, ClHSP38.8, ClHSP39.8, ClHSP42.6, and ClHSP50.3) had a transmembrane domain in the C- or N-terminal region, respectively.

According to the number of introns, the watermelon *ClHSP20* genes could be divided into three types: Type I genes have no introns (21 *ClHSP20s*); type II genes have one intron (20 *ClHSP20s*); and, type III genes have more than one intron (*ClHSP23.1B*, *ClHSP27.9*, and *ClHSP46.3*) (Figure 1).

### 2.2. Phylogenetic Relationship of Plant HSP20 Members

**Figure 3:**
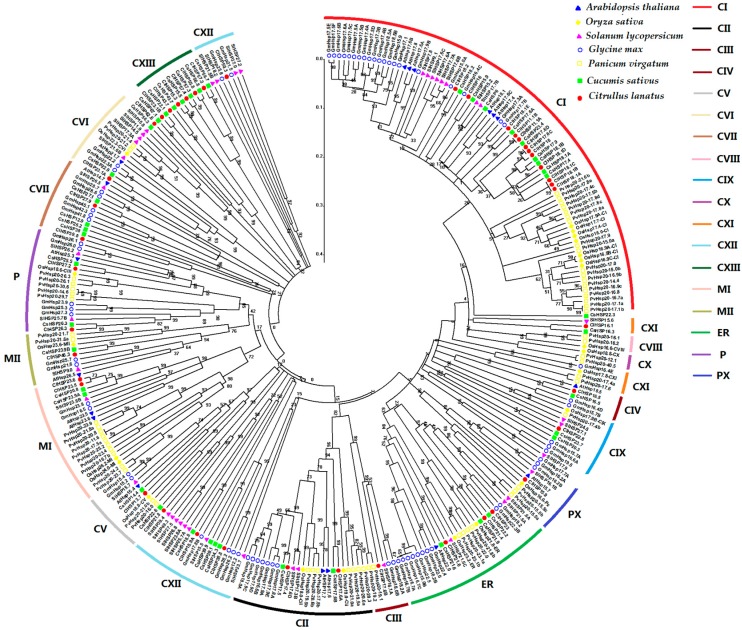
Phylogenetic relationship of HSP20 proteins in *Arabidopsis*, rice, tomato, soybean, switchgrass, cucumber, and watermelon. Phylogenetic trees were constructed using neighbor-joining method with bootstrap tests by MEGA 5.0. The Bar represents the relative divergence of the sequences examined. The diverse HSP20 subgroups are indicated with different color arcs. The different colored symbols at the branch tips represent different species., C, cytoplasmic/nuclear; ER, endoplasmic reticulum; P, plastid; PX, peroxisome; M, mitochondria.

To further analyze the phylogenetic relationships of HSP20 proteins, the amino acid sequences of HSP20 proteins from *Arabidopsis*, rice, tomato, soybean, switchgrass, cucumber, and watermelon were used to perform multiple alignments analyses and to construct a phylogenetic tree (Figure 3). The HSP20s were divided into 18 subfamilies. Notably, a new subfamily, nucleo-cytoplasmic XIII (CXIII), was identified in this study. This subfamily comprised only HSP20 proteins from watermelon and cucumber. Of the 18 subfamilies, 16 contained watermelon HSP20 proteins. Among all the watermelon HSP20 genes, 34 were nucleocytoplasmic (C) HSP20 genes (11 subfamilies); three were mitochondrial (M) HSP20 genes (two subfamilies); three were endoplasmic reticulum (ER) HSP20 genes; two were plastidic (P) HSP20 genes; one was a peroxisomal (Px) HSP20 gene; and, one was an orphan gene (ClHSP42.6). Watermelon had no HSP20 genes in the CVIII or CX subfamilies, since these two subfamilies were exclusively found in monocots. The CIV and CXIII subfamilies each had only one dicotyledon HSP20 gene. The largest subfamily was CI, which contained 12 ClHSP20 genes. Members of the CI subfamily were highly conserved. In fact, the gene pairs ClHSP11.1A and ClHSP11.1B, ClHSP17.6C and ClHSP17.6D, and ClHSP18.1A and ClHSP18.1D in CI subfamily shared identical amino acid sequences with each other (Table S2). 

### 2.3. Genomic Distribution and Evolutionary Analysis of Watermelon HSP20s

**Figure 4:**
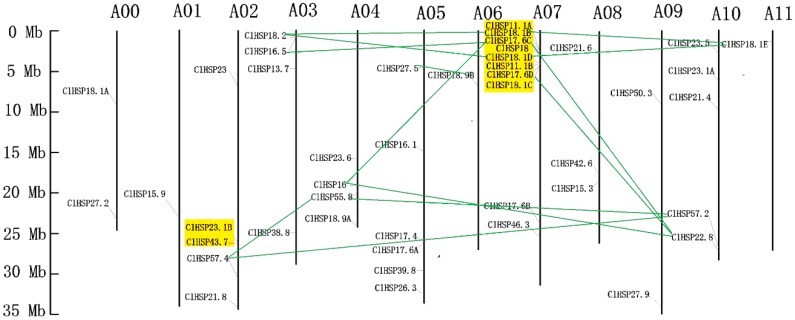
Chromosomal distribution of *HSP20* genes in watermelon. Chromosome number is indicated at the top of each chromosome. Tandemly duplicated genes are highlighted in yellow. Duplicated gene pairs are linked by green line.

All of the *HSP20* genes in watermelon were non-randomly located on the 11 watermelon chromosomes (Chr), except for *ClHSP18.1A* and *ClHSP27.2*. Chr07 had the most *ClHSP20* genes. Only one *ClHSP20* was mapped to each of Chr01, Chr06, and Chr11 (Figure 4). Three tandem duplication clusters involving in 10 *ClHSP20* genes were identified (Table 2). A duplicated gene pair (*ClHSP23.1B* and *ClHSP43.7*) was mapped on Chr02. This pair shared 54.2% amino acid sequence similarity (Table S2). The *Ks* value (i.e., synonymous substitution rate) for this duplicated pair was 1.7552, with the corresponding divergence time being 135.02 million years ago (Mya). *ClHSP11.1A*, *ClHSP18.1B*, *ClHSP17.6C*, and *ClHSP18* were tandemly repeated on Chr 07 and shared 69.1% to 96.8% similarity with each other (Figure 4 and Table S2). The *Ks* values of these duplicates ranged from 0.0865 to 1.2543 corresponding to divergence times of 6.65 to 96.48 Mya (Table 2). *ClHSP18.1D*, *ClHSP11.1B*, ClHSP18.1C, and *ClHSP17.6D* were also distributed on Chr 07 and showed 70.1% to 90.6% amino acid similarity. The *Ks* values of these duplicated genes ranged from 0.3202 to 1.1953, corresponding to divergence times of 24.63 to 91.95 Mya. Interestingly, all of the eight tandem duplicates mapped on Chr 07 belonged to the CI subfamily, suggesting that the watermelon CI subfamily has probably expanded via tandem duplication. Thirteen gene pairs involving 14 *ClHSP20s* resulted from segment duplication (Table 2). Eight pairs of segment duplicates occurred between four *ClHSP20s* on Chr 07 and five genes that were scattered on Chr 03, Chr 04, Chr 10, and Chr 11. Two putative duplicated gene pairs (*ClHSP16/ClHSP22.8* and *ClHSP55.8/ClHSP57.2*) were present on Chr 4 and Chr 10. *ClHSP57.4* on Chr 02 was mapped to a duplicated region shared with *ClHSP55.8* on Chr 04 and with *ClHSP57.2* on Chr 10. Another duplicate pair was detected between *ClHSP27.5* on Chr 05 and *ClHSP18.9B* on Chr 06 (Figure 4). The *Ks* vakues of these segment duplicated gene pairs ranged from 1.54 to 19.78, corresponding to divergence times of 118.42 to 1521.36 Mya. The *Ka/Ks* values of all tandem and segment duplicates were less than 1, indicating that they have undergone purifying selection.

**Figure 5:**
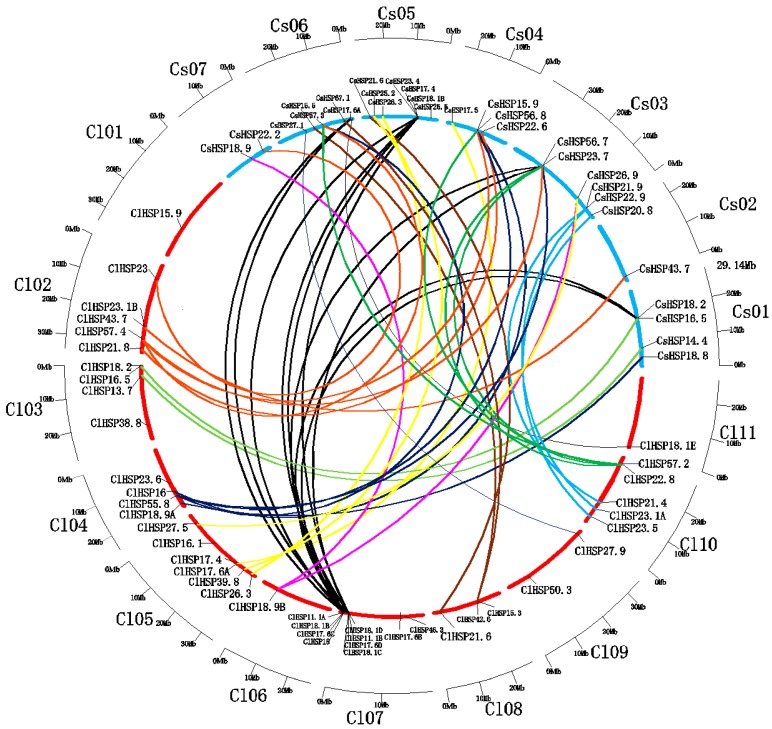
Relationship diagrams of syntenic *HSP20* genes in chromosomal synteny regions distributed in watermelon and cucumber. Chromosomes of watermelon and cucumber are represented by blue and red arcs according to their own sizes. Differently colored lines link representative syntenic HSP20 genes in watermelon and cucumber.

There is a high degree of intergenomic homology between watermelon and cucumber, which belong to the Cucurbitaceae family [33]. Therefore, we assessed the syntenic relationships of *HSP20* genes from watermelon and cucumber genomes using the Cucurbit Genomics Database to investigate cucurbit chromosomal evolution. A total of 42 syntenic gene pairs involving in 30 *ClHSP20s* that were located on all watermelon chromosomes, except for Chr 01, were identified between watermelon and cucumber. Four genes (*ClHSP17.6C*, *ClHSP17.6D*, *ClHSP18.1B*, and *ClHSP18.1D*) on watermelon Chr07 had the highest number of syntenic genes (10) in the cucumber genome (Figure 5).

### 2.4. Analysis of Putative cis-Acting Elements in ClHSP20 Promoters

Heat shock elements (HSE) are common *cis*-acting elements in the promoters of *HSP20s* that confer heat shock-inducible expression. We identified and analyzed *cis*-acting elements in the putative promoter regions of the HSP20 genes in watermelon (Table S3). Numerous hormone- and stress-responsive elements were found in the *ClHSP20* promoters. We detected MeJA-, GA-, salicylic acid (SA)-, and ABA- responsive elements in 23, 20, 20, and 10 *HSP20* promoters, respectively. Of the 44 watermelon *HSP20* genes, 27 had HSE motifs in their promoter regions. Drought- and low temperature- responsiveness motifs were identified in the promoters of 8 and 10 *ClHSP20* genes, respectively. 

### 2.5. Subcellular Localizations of HSP20 Proteins in Watermelon

**Figure 6:**
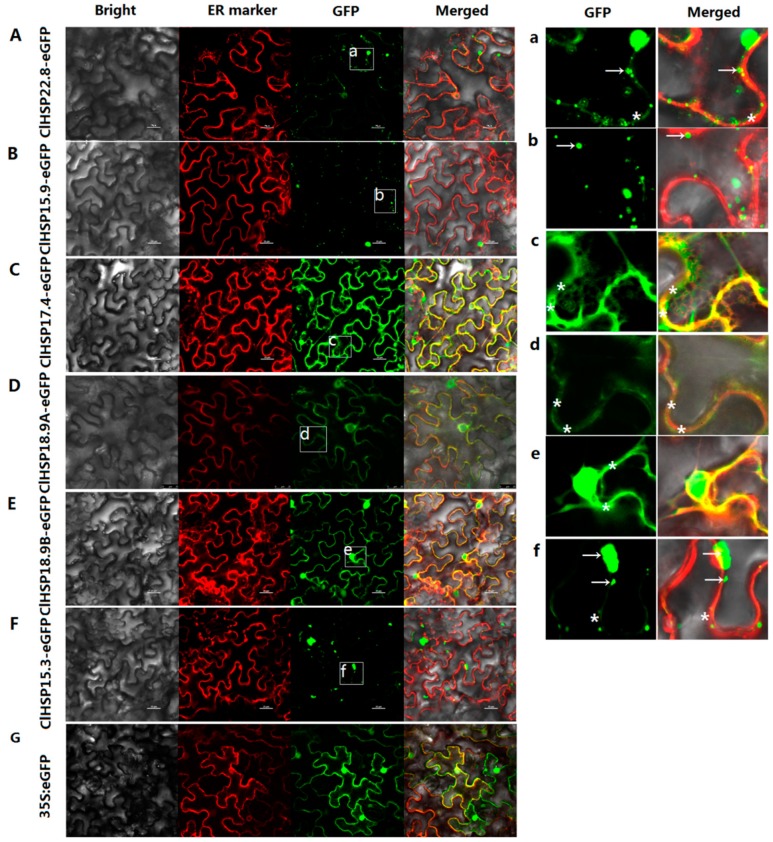
Subcellular localizations of ClHSP20 proteins. Green fluorescent protein (GFP)-fusion proteins were transiently expressed in tobacco leaves. The results were observed after a 48 h-incubation. Bright-field, endoplasmic reticulum (ER) marker (red fluorescence), green fluorescence (GFP), and merged images (from left to right) of 35S:ClHSP22.8-EGFP (**A**); 35S:ClHSP15.9-EGFP (**B**); 35S:ClHSP17.4-EGFP (**C**); 35S:ClHSP18.9A-EGFP (**D**); 35S:ClHSP18.9B-EGFP (**E**); 35S:ClHSP15.3-EGFP (**F**); and, 35S:EGFP (**G**) in tobacco leaf epidermal cells. (**a**–**f**) displays enlarged images of the section delimited by a white square in A to F, respectively. Green fluorescence signal indicated the localizations of ClHSP20 fusion proteins. Red fluorescence signal indicated endoplasmic reticulum (ER) marker. White arrows indicated fluorescent granules. White stars labeled the GFP fluorescent signal located on endoplasmic reticulum. Scale bars, 25 μm.

**Figure 7:**
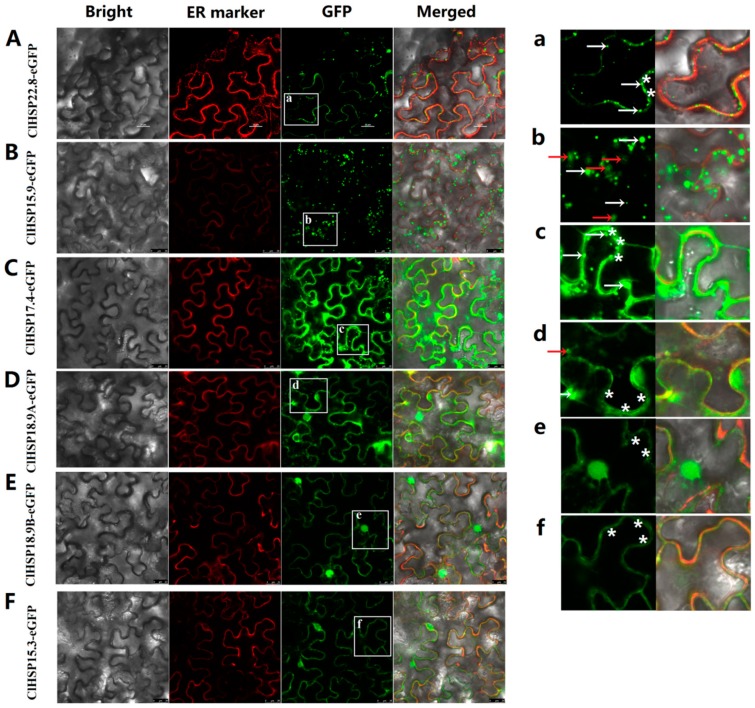
Subcellular localizations of ClHSP20 proteins under *Cucumber green mottle mosaic virus* (CGMMV) infection. Green fluorescent protein (GFP)-fusion proteins were transiently expressed in tobacco leaves with CGMMV infectious clones. Fluorescence signal was detected after 48 h of incubation. Bright-field, endoplasmic reticulum (ER) marker (red fluorescence), green fluorescence (GFP), and merged images (from left to right) of 35S: ClHSP22.8-EGFP (**A**); 35S: ClHSP15.9-EGFP (**B**); 35S:ClHSP17.4-EGFP (**C**); 35S:ClHSP18.9A-EGFP (**D**); 35S:ClHSP18.9B-EGFP (**E**); and, 35S:ClHSP15.3-EGFP (**F**) in tobacco leaf epidermal cells. (**a**–**f**) displays enlarged images of the section delimited by a white square in A to F, respectively. Fluorescent granules were indicated by white arrows. Vesicae were marked by red arrows. White stars labeled the GFP fluorescent signal located on endoplasmic reticulum. Scale bars, 25 μm.

The subcellular localizations of six watermelon HSP20 proteins from different subfamilies, namely ClHSP22.8, ClHSP15.9, ClHSP17.4, ClHSP18.9A, ClHSP18.9B, and ClHSP15.3, were analyzed by transiently expressing their green fluorescent protein (GFP) fusion proteins in tobacco leaf epidermal cells. The results showed that these six ClHSP20 proteins were localized to various subcellular compartments, the watermelon ClHSP20 proteins have diverse functions (Figure 6). Specifically, ClHSP15.9 was predicted to be located in peroxisomal according to the WoLF PSORT website, however, it was predominantly detected in cytoplasmic granules (Table S1 and Figure 6B,b). ClHSP22.8 and ClHSP15.3-GFP were predicted to be cytoplasmic and nuclear proteins, respectively, but their GFP fusion proteins were mainly agglomerated into granules in cytoplasm, and they were weakly detected in endoplasmic reticulum (ER) (Figure 6A,F,a,f). In contrast, ClHSP18.9A, ClHSP17.4, and ClHSP18.9B were predicted to be located in golgi, cytoplasm, and chloroplasts, respectively, but, in fact, the corresponding GFP fusion proteins were localized to the endoplasmic reticulum (Figure 6C–E,c–e). Additionally, ClHSP17.4-, ClHSP18.9A-, ClHSP18.9B-, and ClHSP15.3-GFP fusion proteins were also detected in nuclear (Figure S2). After CGMMV infections, the subcellular localizations of some ClHSP20s appeared to change (Figure 7). For example, ClHSP15.9-GFP fusion proteins accumulated into more vesicles and some newly formed vesicae in the cytoplasm, whereas ClHSP17.4-GFP began to agglomerate in relatively large granules along the endoplasmic reticulum, and ClHSP15.3-GFP appeared to no longer agglomerate in granules, but instead it was present at endoplasmic reticulum. 

### 2.6. Expression Analyses of ClHSP20 Genes in Response to Abscisic Acid and Melatonin 

**Figure 8:**
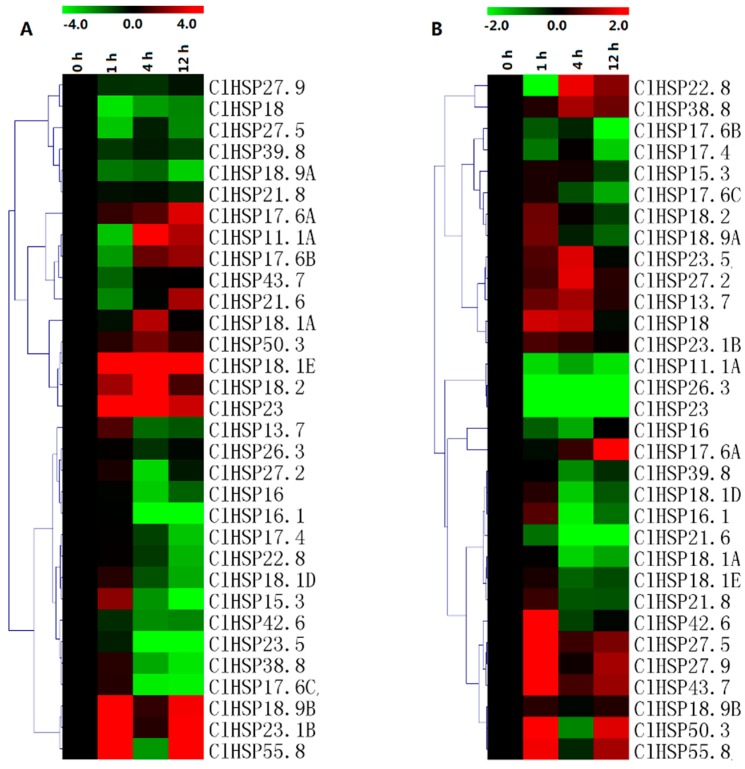
Heat map representation of the responses of watermelon *ClHSP20* genes to exogenous melatonin. (**A**) and abscisic acid (**B**). Watermelon leaves were collected at 0, 1, 4, and 12 h after 150 μM melatonin or 100 μM abscisic acid treatment. Heatmaps were constructed with the MeV4.8 program. Color scales representing the relative expression values are shown on the upper left of heatmap (log_2_ scale).

The expression patterns of 30 randomly selected *ClHSP20* genes in response to ABA and melatonin (MT) were analyzed in a qRT-PCR assay (Figure 8, Table S6). Most of the *ClHSP20s* could respond to ABA and MT. Under ABA treatment, the expression levels of 14 *ClHSP20s* were obviously downregulated from 4 h, while eight *ClHSP20s* exhibited upregulated expression. Of these genes, *ClHSP18.1E*, *ClHSP18.2*, and *ClHSP23* expression levels increased by 70-, 40-, and 39-fold, respectively, at 4 h after the ABA treatment (Figure 8A, Table S6). Some *ClHSP20s*, such as *ClHSP11.1A*, *ClHSP17.6B*, and *ClHSP21.6,* were induced after repressed by ABA treatment. In contrast, MT downregulated the expression of most of the analyzed *ClHSP20* genes, especially at 4 and 12 h after treatments. Moreover, the expression levels of some *ClHSP20* genes, including *ClHSP11.1A*, *ClHSP26.3*, *ClHSP21.6*, and *ClHSP23*, were downregulated from 1 to 12 h after the MT treatment (Figure 8B). Meanwhile, MT upregulated the expression of 11 *ClHSP20* genes, especially at 1 h after treatments. The *ClHSP17.6A* expression level increased by 145-fold at 12 h, whereas *ClHSP22.8* expression was evidently downregulated at 1 h, but it increased by nearly three-fold at 4 h after the MT treatment.

### 2.7. Expression Analyses of ClHSP20 Genes in Response to Heat and CGMMV 

**Figure 9:**
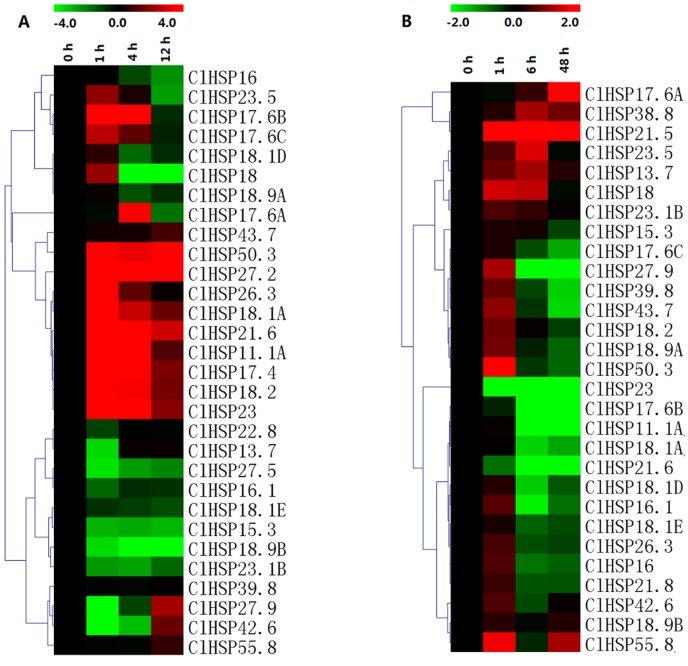
Heat map representation of response patterns of watermelon *HSP20* genes to heat stress (**A**) and CGMMV infection (**B**). For heat treatment, leaves were collected at 0, 1, 4, and 12 h after 42 °C treatment. Watermelon leaves were collected at 0, 1, 6, and 48 h after CGMMV infection. For other details, see Figure 7.

The qRT-PCR results indicated that the expression levels of only 11 of 30 analyzed *ClHSP20* genes were upregulated by heat stress (Figure 9, Table S6). We observed that *ClHSP11.1A*, *ClHSP50.3*, and *ClHSP17.4* expression levels considerably increased (>100-fold) in response to heat stress. Meanwhile, 11 genes exhibited the opposite trend following an exposure to heat, especially *ClHSP27.5*, *ClHSP18.2*, *ClHSP15.3*, *ClHSP18.9B*, and *ClHSP23.1B*. Infection by CGMMV induced eight watermelon *HSP20s*, especially *ClHSP17.6A,* and *ClHSP21.5*, which showed drastic increases in their transcript levels (145- and 25022-fold, respectively). Nevertheless, nine *ClHSP20s* (*ClHSP11.1A*, *ClHSP16.1*, *ClHSP17.6B*, *ClHSP17.6C*, *ClHSP18.1A*, *ClHSP18.1D*, *ClHSP23*, *ClHSP21.6*, and *ClHSP43.7*) were obviously repressed by CGMMV infection from 6 h. The *ClHSP27.9* expression level was upregulated at 1 h, but it was downregulated at 6 and 48 h after the CGMMV infection. The other *ClHSP20* genes appeared unresponsive to CGMMV.

### 2.8. Gene Ontology Enrichment Analysis 

**Figure 10:**
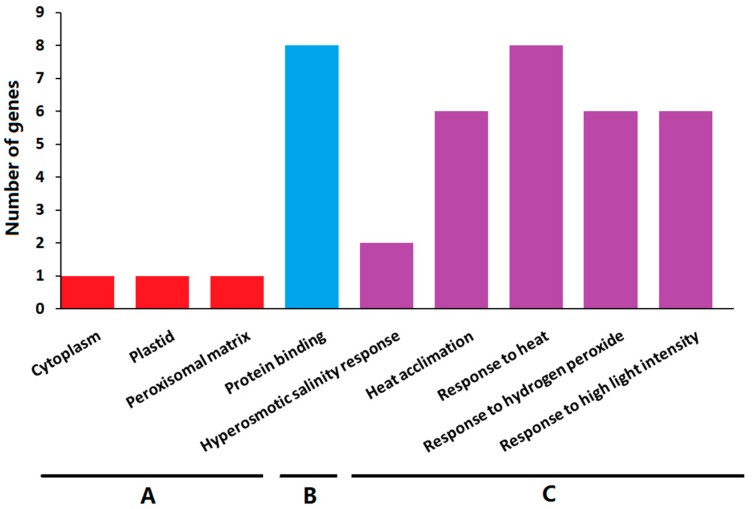
The Gene Ontology (GO) analysis of *HSP20* genes in watermelon. The *ClHSP20* genes were categorized into three groups: cell component (**A**); biological process (**B**); and, molecular function (**C**).

To further explore their functions, the *ClHSP20* genes underwent a Gene Ontology (GO) enrichment analysis. The enriched GO terms were grouped into the following three categories; biological process, molecular function, and cellular component (Figure 10 and Table S4). The significantly enriched molecular function GO term for molecular function was protein binding (GO:0005515). The ClHSP20 proteins were mainly located in the cytoplasm, plastid, and peroxisomal matrix. The main enriched biological process terms were hyperosmotic salinity response (GO:0042538), heat acclimation (GO:0010286), response to heat (GO:0009408), response to hydrogen peroxide (GO:0042542), and response to high light intensity (GO:0042538). The results of the GO enrichment analysis suggested that the *ClHSP20* genes have significant roles in various stress responses.

## 3. Discussion

The *HSP20* gene family has been identified in *Arabidopsis*, rice, soybean, wheat, barley, tomato, pepper, and switchgrass, respectively [2,22,25,27,28,29,34,35]. The number of *HSP20* gene family members ranges from 13 (barley) to 63 (soybean). In this study, we identified 44 *ClHSP20s* in watermelon, fewer than in switchgrass (63), soybean (51), and cucumber (45), but more than in the other species (Table 1). Although the genome size of watermelon (425 Mb) is obviously bigger than in *Arabidopsis* (125 Mb) and cucumber (367 Mb), the sizes of many gene families in watermelon are similar to those in cucumber but slightly smaller than those in *Arabidopsis* [36,37,38]. This is probably because watermelon avoided more recent wholegenome duplication events, except for the core-eudicot common hexaploidization (ECH) event [39,40]. However, the number of *HSP20s* in watermelon is twice than that in *Arabidopsis* in this study, which appears to contradict the above findings [39], but is consistent with the latest research that a cucurbit-common tetraploidization (CCT, 90–102 Mya) event occurred in the Cucurbitaceae shortly after the ECH event (115–130 Mya) [33]. The 13 tandemly duplicated gene pairs of *ClHSP20s* arose 6.65 to 135.02 Mya, probably as a result of gene losses and chromosomal rearrangements after the CCT event, However, there are relatively low rates of preserved CCT colinear genes in the watermelon genome (2.5–5.5%), which explains why many other watermelon gene families are quite small [36,37,38,39]. Watermelon has retained high genomic homology and it shares more colinear genes with cucumber [33]. Therefore, we analyzed the linear relationships between *HSP20* genes in watermelon and cucumber in this study. Forty-two colinear gene pairs between the two species were identified (Figure 5). Some *HSP20s* in the watermelon genome had more than one syntenic gene in the cucumber genome. These watermelon colinear genes were distributed on all of the chromosomes, except for Chr 01, with the largest number of colinear genes on Chr 07. The existence of so many colinear genes and colinear blocks between the watermelon and cucumber genomes was probably due to another round of genomic repatterning after the CCT event [33]. 

The *HSP20* genes are important for responses to various stresses [3,5,6,14,15]. In this study, GO enrichment results indicated that *ClHSP20* genes were mainly enriched in the heat, salinity, hydrogen peroxide, and high light signal response pathway. The *HSP20* genes are characterized by high and rapid upregulation in response to heat stress. Almost all of *HSP20s* from soybean, pepper, and switchgrass exhibit upregulated expression in response to heat stress [21,28,29]. In watermelon, all *HSP20s* could respond to heat stress, except for *ClHSP39.8*, *ClHSP43.7*, and *ClHSP55.8*. However, the expression levels of approximately half of the *ClHSP20* genes are repressed by heat shock, especially *ClHSP27.5*, *ClHSP18.1E*, *ClHSP16.1*, *ClHSP18.9B*, *ClHSP15.3*, and *ClHSP23.1B*. The induced *HSP20s* could enhance heat tolerance by protecting proteins from irreversible denaturation, but it is necessary to elucidate the roles of these downregulated genes [41]. Infections by CGMMV are devastating for cucurbit crops, and they have been responsible for considerable watermelon yield losses [42]. However, little is known about the molecular mechanism underlying the CGMMV-induced watermelon disease. In response to CGMMV infection, most watermelon *HSP20s* were obviously repressed from 6 h after being slightly induced at 1 h in this study. Just few reports indicated plant *HSP20s* involved in viral response. Microarray technology analysis indicated that *Arabidopsis HSP17.4* was induced under five plant viruses infection and it was speculated to have a common mechanism in response to viral infection, but its homologous gene, *ClHSP18.1E* in watermelon, was repressed by CGMMV infection [43]. The RNA-Seq data analysis found that two *HSP20s* from ER subfamily, namely, *ClHSP23* and *ClHSP21.6*, in CGMMV-inoculated watermelon fruits, were significantly repressed, which had similar responses in leaves in this study [44]. The results suggested that ER subfamily genes in watermelon probably played negative roles in response to CGMMV. 

Recently, studies found that melatonin widely participated in plant biotic and abiotic stress responses, even it has begun to be considered by some experts in plant hormones [45]. *HSP20s* have been to proved to participate in stress responses via the MT signaling pathway, and the expression levels of tomato *HSP20s* increased under heat and cadmium stress after melatonin treatment [46,47]. In this study, the qRT-PCR results indicated that most of the watermelon *HSP20* genes were induced by MT at 1 h but obviously repressed at 4 and 12 h. Similarly, most of the watermelon *HSP20s* were also generally repressed by ABA treatment, as well as the expression levels of nearly all switchgrass *HSP20* genes are downregulated in response to an ABA treatment [29]. Notably, more *ClHSP20s* has similar response patterns to ABA, MT, and CGMMV, as they were generally repressed by the three treatments. Take the newly identified CXIII subfamily gene *ClHSP55.8* and *ClHSP15.3* as examples, following ABA and MT treatments, the *ClHSP55.8* transcript abundance dramatically increased at 1 h, decreased at 4 h, and then increased to high levels at 12 h. Similarly, *ClHSP55.8* expression was induced by CGMMV at 1 and 48 h, but was repressed at 6 h. In contrast, *ClHSP15.3* expression was repressed by ABA, MT, and CGMMV, except for a transient increase at 1 h in response to ABA. These findings imply that these *ClHSP20* genes probably participate in a common signaling pathway in response to MT, ABA, and CGMMV.

Plant HSP20 proteins have been detected in various cellular locations, including the cytosol, nucleus, chloroplast, endoplasmic reticulum, mitochondrion, and peroxisome [25]. This diversity in subcellular localization is probably due to the multiple functions for these proteins. To analyze the subcellular localization and distribution pattern of watermelon HSP20 proteins, six ClHSP20s fused with GFP at their N terminus were constructed and introduced into tobacco epidermal cells. The ClHSP20s displayed different subcellular localizations that were expressed alone in tobacco epidermal cells. The fluorescence signal of ClHSP18.9A-, ClHSP17.4-, and ClHSP18.9B-GFP proteins were mainly detected in endoplasmic reticulum and nuclear (Figure 6, Figure S2). ClHSP22.8, ClHSP15.9, and ClHSP15.3 formed different fluorescent granules in cytoplasm. These granules probably were large oligomers that were formed in vitro by HSP20 proteins as previous reports (Figure 6) [48]. Recently, a study found that the *Rice stripe virus* could alter the sub-cellular distribution of HSP20s in rice and tobacco [23]. In this study, some ClHSP20-GFP fusion proteins, including ClHSP15.9-GFP, ClHSP17.4-GFP, and ClHSP15.3-GFP, were affected by CGMMV, especially regarding the size, number, and distribution of the granules that they formed (Figure 7). The ClHSP15.9-GFP and ClHSP17.4-GFP proteins primarily accumulated in granules. Notably, ClHSP15.9-GFP proteins formed new cytoplasmic vesicles. In contrast, the fluorescence of ClHSP15.3-GFP proteins that was initially detected in granules was subsequently mainly observed in the membrane. These granules or vesicles detected in this study were similar to stress granules, and were identified in animal cells in response to virus infection. The formation of stress granules probably was an anti-viral response [23,49]. A more thorough characterization of these granules will likely clarify the mechanism underlying ClHSP20 responses to viral infections.

## 4. Materials and Methods 

### 4.1. Identification of HSP20s in Watermelon and Cucumber

Protein sequences of all known *HSP20* genes, that is, 19, 23, 42, 27, 13, 35, 51, and 63 members of the *HSP20* family in *Arabidopsis*, rice, tomato, wheat, barely, pepper, soybean, and switchgrass, respectively, were downloaded from Phytozome (http://phytozome.jgi.doe.gov/pz/portal.html). These sequences were then used as queries to carry out BLASTP searches with E-value of 1 × 10^−5^ as the threshold. Meanwhile, Hidden Markov Model (HMM) profiles were generated using HSP20 conserved domain sequences from Pfam (http://pfam.janelia.org/). The HMM profiles were used to identify the putative watermelon and cucumber HSP20 proteins using HMMER 3.0 software (http:// hmmer.janelia.org/) with a default E-value. Finally, redundant sequences were omitted to retain unique HSP20 genes. ExPASy (http://web.expasy.org/compute_pi/) was used to calculate the molecular weights and isoelectric points (PIs) of putative watermelon HSP20 proteins. Subcellular localizations were predicted using WoLF PSORT website (https://wolfpsort.hgc.jp/).

### 4.2. Phylogenetic Analysis, Gene Structure Construction, and Motif Analysis

The similarity of HSP20s from *Arabidopsis*, watermelon, and cucumber was calculated using DNAStar software (Madison, WI, USA). Phylogenetic analysis based on full-length protein sequences was performed using the MEGA 5.0 program by the neighbor-joining (NJ) method with 1000 bootstrap replicates [50]. The structures of all watermelon *HSP20* genes were analyzed via the Gene Structure Display Server (http://gsds.cbi.pku.edu.cn/). Motif analysis and annotation of conserved motifs in HSP20 proteins were conducted using MEME (http://meme.nbcr.net/meme/cgi-bin/meme.cgi). The predicted peptide sequences of conserved domains in HSP20 proteins were confirmed using CDD (https://www.ncbi.nlm.nih.gov/Structure/bwrpsb/bwrpsb.cgi) and SMART databases (http://smart.emblheidelberg.de/). Multiple-sequence alignment of predicted peptide sequences of the conserved α-crystallin (ACD) domain was carried out using Clustal X v1.81 with default parameters [51]. 

### 4.3. Chromosomal Localization, Gene Duplication, and Evolutionary Analysis 

All *HSP20* genes were assigned to corresponding watermelon chromosomes based on the Cucurbit Genomics Database. Gene pairs that were separated by fewer than five intervening genes and sharing ≥40% amino acid sequence similarity were considered to have undergone a tandem duplication event [52]. The synonymous (*Ks*) and non-synonymous (*Ka*) substitution rates were estimated, as described by Tang et al., 2008 [53]. Synteny analysis was performed with the Cucurbit Genomics Database. CLUSTALW (http://www.genome.jp/tools/clustalw/) was used to align the amino acid sequences and corresponding CDS sequences of HSP20 elements, and then *Ks* and *Ka* values were calculated using the Codeml procedure of the PAML (http://www.bork.embl.de/pal2nal/). The divergence time of each duplicated gene pair was estimated using the synonymous mutation rate of substitutions per synonymous site per year, as follows: T = *Ks*/2x (x = 6.56 × 10^−9^) [54]. Gene Ontology (GO) enrichment analysis was performed while using AgBase (http://agbase.arizona.edu/cgi-bin/tools/index.cgi).

### 4.4. Analysis of cis-Acting Elements in HSP20 Putative Promoter Regions in Watermelon

To identify the *cis*-elements in the promoter sequences of *HSP20* genes in watermelon, the 1.5-kb upstream regions were obtained from the Cucurbit Genomics Database, and analyzed using the online tools at the PlantCARE website (http://bioinformatics.psb.ugent.be/webtools/plantcare/html/search_CARE.html).

### 4.5. Watermelon Plant Growth and Treatments

The watermelon advanced inbred line ‘JJZ-M’ was used for expression analyses. The plants were grown in a growth chamber in temperature-controlled greenhouses under day/night temperatures of 28/22 ± 1 °C, light intensity of 200 μmol·m^−2^·s^−1^, and a 16-h light/8-h dark photoperiod. Three-week-old watermelon seedlings were used for stress and exogenous hormone treatments. For MT and ABA treatments, the seedlings were sprayed with 150 μM MT and 100 μM ABA, respectively [38,55]. The second true leaf on each plant was sampled at 0 (control), 1, 4, and 12 h after treatment. In the heat treatment, watermelon plants were subjected to heat treatment at 42 °C and the leaves were collected at 0 (control), 1, 4, and 12 h. For CGMMV infection, transformed *Agrobacterium* cells containing the infectious full-length cDNA of the CGMMV genome were incubated at 28 °C for two days. The cells were then resuspended in infiltration solution (500 mM acetosyringone, 10 mM MES, pH 5.8, and 10 mM MgCl_2_) and injected into the abaxial side of watermelon leaves, as described by Voinnet et al., 2003 [56]. Infiltrated leaves were collected at 0 (control), 1, 6, and 48 h after infection. All treatments were repeated three times and each treatment contained 20 seedlings. All materials were frozen at −75 °C until RNA isolation. 

### 4.6. RNA Isolation and qRT–PCR

The total RNA was extracted from samples using TRIZOL reagent (Invitrogen, Germany), according to the manufacturer’s protocol. First-strand cDNA was generated from 1 μg of total RNA using the PrimeScript RT reagent kit (Takara, Japan), according to the manufacturer’s instructions. Specific primers that were used in the qRT-PCR were designed using Primer 5 software, and each primer was searched in the watermelon database to ensure its specificity. The qRT-PCR reactions (reaction volume, 15 μL) were performed on a CFX96 Real Time System machine (Bio-RAD, USA), programmed to heat for 30 s at 95 °C, followed by 40 cycles of 5 s at 95 °C and 45 s at 55 °C, and at the end, one cycle of 1 min at 95 °C, 30 s at 50 °C, and 30 s at 95 °C. Two biological and three technical replicates for each sample were analyzed using the SYBR Premix Ex Taq kit (TOYOBO, Japan). Watermelon *β-actin* (*Cla007792*) was selected as an internal control [57]. The relative gene expression level was calculated using the 2^−ΔΔ*C*t^ method. The heatmap was generated from relative gene expression data using Multiple Array Viewer.

### 4.7. Subcellular Localization Analyses

The CDS sequences of *ClHSP22.8*, *ClHSP15.9*, *ClHSP17.4*, *ClHSP18.9A*, *ClHSP18.9B*, and *ClHSP15.3* were amplified using gene-specific primers and they were cloned into the pFGC-eGFP plasmid via the *Xba* I and *BamH* I restriction sites (Table S5). Recombinant expression vectors of ClHSP20s fused to the CDS of enhanced green fluorescent protein (eGFP) protein were constructed. These plasmids were transformed into *Agrobacteriumt tumefaciens* GV3101 and transiently expressed in tobacco leaf cells with (or without) transformed *Agrobacterium* containing the infectious full-length cDNA clone of the CGMMV genome. The pFGC:eGFP empty vector served as the positive control. Images were acquired at 48 h using a Leica DMLE camera (Leica, Wetzlar, Germany).

## 5. Conclusions

HSP20s are the most abundant HSPs in plants and they play important roles in various biotic and abiotic stresses. However, *HSP20* genes in watermelon had not been systematically analyzed. Here, 44 *HSP20* genes in watermelon were identified and their gene structure, conserved domains, phylogenetic relationships, chromosome evolution, expression profiles, and subcellular localizations were analyzed. All of the watermelon HSP20 proteins contained a conserved α-crystallin (ACD) domain. Plant HSP20s could be divided into 18 subfamilies and a new subfamily, nucleo-cytoplasmic XIII (CXIII) was identified in this study. Of the 26 duplicated gene pairs in the watermelon genome, 13 arose by tandem duplication and 13 arose by segmental duplication. Numerous stress- and hormone-responsive *cis*-elements were detected in the putative promoter regions of the watermelon *HSP20* genes. Almost all *HSP20s* from soybean, pepper, and switchgrass induced by heat stress, but about half of the watermelon HSP20s were repressed by heat stress via qRT-PCR analyse. Plant *HSP20s* displayed diverse responses to different virus infections in previous studies and most of the *ClHSP20s* were generally repressed by *Cucumber green mottle mosaic virus* (CGMMV). Several *ClHSP20s* showed similar response patterns to ABA, MT, and CGMMV. Subcellular localization analyses of six selected HSP20-GFP fusion proteins revealed diverse subcellular targeting in the epidermal cells of *N. benthamiana*. The subcellular localization and distribution patterns of ClHSP20 proteins, especially the size, number, and distribution patterns of the granules formed by ClHSP20-GFP proteins, were markedly affected by CGMMV infection. This systematic analysis will provide a foundation for elucidating the physiological functions and biological roles of the *HSP20* family.

## Figures and Tables

**Table 1:** Summary of the number of *heat shock protein 20 (HSP20)* genes in diverse plant species.

Species	The Number of HSP20s	Reference
*Arabidopsis thaliana*	19	[25]
*Oryza* *sativa*	23	[2]
*Solanum lycopersicum*	42	[26]
*Triticum aestivum*	27	[27]
*Hordeum vulgare*	13	[27]
*Capsicum annuum*	35	[28]
*Glycine max*	51	[21]
*Panicum virgatum*	63	[29]
*Cucumis sativus*	45	This work
*Citrullus lanatus*	44	This work

**Table 2:** *Ka/Ks* calculation and divergent time of the duplicated watermeolon *HSP20* gene pairs.

Duplicated Gene Pairs	*Ks*	*Ka*	*Ka/Ks*	Duplicated Type	Purify Selection	Time * (MYA)
*ClHSP22.8/ClHSP17.6D*	19.7650	0.5612	0.0284	Segmental	Yes	1520.38
*ClHSP22.8/ClHSP17.6C*	19.7777	0.5611	0.0284	Segmental	Yes	1521.36
*ClHSP22.8/ClHSP16*	12.8876	0.4231	0.0328	Segmental	Yes	991.35
*ClHSP18.2/ClHSP18.1B*	2.4385	0.2044	0.0838	Segmental	Yes	187.58
*ClHSP18.2/ClHSP18.1D*	1.6708	0.1864	0.1115	Segmental	Yes	128.52
*ClHSP18.1E/ClHSP18.1D*	2.4264	0.1057	0.0435	Segmental	Yes	186.65
*ClHSP18.1E/ClHSP18.1B*	2.5427	0.1239	0.0487	Segmental	Yes	195.59
*ClHSP18.9B/ClHSP27.5*	3.4679	0.2528	0.0729	Segmental	Yes	266.76
*ClHSP55.8/ClHSP57.2*	1.5394	0.1181	0.0767	Segmental	Yes	118.42
*ClHSP55.8/ClHSP57.4*	3.2918	0.1509	0.0458	Segmental	Yes	253.22
*ClHSP57.2/ClHSP57.4*	3.0423	0.1941	0.0638	Segmental	Yes	234.02
*ClHSP16/ClHSP17.6C*	9.3352	0.1704	0.0183	Segmental	Yes	718.09
*ClHSP16.5/ClHSP17.6C*	2.7813	0.5545	0.1994	Segmental	Yes	213.95
*ClHSP11.1A/ClHSP18.1B*	0.3202	0.2266	0.7076	Tandem	Yes	24.63
*ClHSP11.1A/ClHSP17.6C*	1.1953	0.2548	0.2131	Tandem	Yes	91.95
*ClHSP11.1A/ClHSP18*	1.2543	0.2635	0.2101	Tandem	Yes	96.48
*ClHSP18.1B/ClHSP17.6C*	0.8027	0.0525	0.0653	Tandem	Yes	61.75
*ClHSP18.1B/ClHSP18*	0.8097	0.0517	0.0639	Tandem	Yes	62.28
*ClHSP17.6C/ClHSP18*	0.0865	0.0152	0.1756	Tandem	Yes	6.65
*ClHSP18.1D/ClHSP11.1B*	0.7433	0.2644	0.3557	Tandem	Yes	57.18
*ClHSP18.1D/ClHSP18.1C*	0.4589	0.0427	0.0931	Tandem	Yes	35.30
*ClHSP18.1D/ClHSP17.6D*	0.6050	0.0565	0.0934	Tandem	Yes	46.54
*ClHSP11.1B/ClHSP18.1C*	0.3202	0.2266	0.7076	Tandem	Yes	24.63
*ClHSP11.1B/ClHSP17.6D*	1.1953	0.2548	0.2131	Tandem	Yes	91.95
*ClHSP18.1C/ClHSP17.6D*	0.8027	0.0525	0.0653	Tandem	Yes	61.75
*ClHSP43.7/ClHSP23.1B*	1.7552	0.4791	0.2730	Tandem	Yes	135.02

* MYA, million years ago.

## Supplementary Materials

Supplementary materials can be found at http://www.mdpi.com/1422-0067/20/1/12/s1.
