# Supplementary material for: Genome-Wide Analysis of Watermelon HSP20s and Their Expression Profiles and Subcellular Locations under Stresses
Source: Int J Mol Sci. 2018 Dec 20;20(1):12. doi: 10.3390/ijms20010012 (PMC6337729; doi:10.3390/ijms20010012)
Supplement: Supplementary file 1 [file ijms-20-00012-s001.zip › Table S5 Primers used in qRT-PCT and sublocation.pdf]

Table S 5.1 Primers used for subcellular localization

Primer Name	Sequences
subClHSP22.8-S	CGGGATCCATGTCGCTCCGTTTCAG
subClHSP22.8-A	GCTCTAGATTAATGGCCAGAAATCTGG
subClHSP15.9-S	CGGGATCCATGGCGAATGATTTGTTTG
subClHSP15.9-A	GCTCTAGATTAAAGCTTGCTAATAATGTTG
subClHSP17.4-S	CGGGATCCATGGATCTCAGAATCATGGGC
subClHSP17.4-A	GCTCTAGATCAATTGACCTTGACCTCAACG
subClHSP18.9A-S	CGGGATCCGAGAAAGGTGAACCATCG
subClHSP18.9A-A	GCTCTAGACTAGTTTTAATCAAGTAGTAAAC
subClHSP18.9B-S	CGGGATCCAGGAAGGTTCGTCCAGTATC
subClHSP18.9B-A	GCTCTAGATTACTGTACAACTACTAAATGGCC
subClHSP15.3-S	CGGGATCCGAAGGCCATGGCGATCAGAC
subClHSP15.3-A	GCTCTAGATCACAGTGGTGAGTGGGAATCG

Table S 5.2 Primers used for Quantitative real-time PCR (qRT-PCR)

Primer Name	Sequences
β-actin (Cla007792)-S	CCATGTATGTTGCCATCCAG
β-actin (Cla007792)-A	GGATAGCATGGGGTAGAGCA
ClHSP22.8-S	CGGATGTGGATAAGGTGAG
ClHSP22.8-A	CGTGGGTTTCTTGAAGTGG
ClHSP27.2-S	CAGGATGGAGATGGAGCAG
ClHSP27.2-A	GGTGTTGGTGGTCTCAGTTTC
ClHSP18.1A-S	TCAAGGCCGATCTTCCC
ClHSP18.1A-A	CTTTCCACTGCTCCGCTC
ClHSP18.2-S	TCGGATAGAGCGAAGCAG
ClHSP18.2-A	CAGCCAGAAATCTCAACG
ClHSP18.1E-S	GGATGCAATTTCCAGGG
ClHSP18.1E-A	CAACCTCCACTTTCACCTC
ClHSP23-S	CGGATTGGAAGGAGACG
ClHSP23-A	AACCTGCCCGAGGAACT
ClHSP16.1-S	GGAGACGCTACTACCGCC
ClHSP16.1-A	GCCCGCCTTCACATTC
ClHSP21.6-S	GCGAGAGTTGACTGGAAAG
ClHSP21.6-A	CGTTCCAATCTATGCCAATG
ClHSP15.9-S	AGGGTTGAGGAGGGAAATG
ClHSP15.9-A	TTCCCAACACTTTCCTCTTTAC
ClHSP21.4-S	CGACCCATTTAGAATCCTTG
ClHSP21.4-A	CATCTTCCTTCTTCATCCCTG
ClHSP17.4-S	GGAGAGAAGAGTCGGCAAG
ClHSP17.4-A	GACAGTCACAGTCAGCACCC
ClHSP16.5-S	CTCTTGGGGAGGCACC
ClHSP16.5-A	ATAAACGCACTCAACTCCTC
ClHSP11.1A-S	AGAATGCTAAAGCGGAGGA
ClHSP11.1A-A	TAGGCACCGTCACAGTCAG

ClHSP26.3-S	AGAACTAACGGAAAGGAACA
ClHSP26.3-A	GCTCGTAAATGGAGGTGAT
ClHSP23.1A-S	GTCCATAAAGAAGACCACCATAC
ClHSP23.1A-A	GTCCATAAAGAAGACCACCATAC
ClHSP38.8-S	GGGACAGCCAGTTCAAG
ClHSP38.8-A	TCTCCTCCGCCATTTT
ClHSP17.6A-S	CGCCCTCCACGACCTTC
ClHSP17.6A-A	GCCGCCATCGCTTTTG
ClHSP46.3-S	GCCGTTCCCTCAACCTATAC
ClHSP46.3-A	GTCAGCCCTGTCCGTGTC
ClHSP17.6B-S	CCTAAAGCCATCTCCACCA
ClHSP17.6B-A	CTCTTCCACCGTCACCTG
ClHSP23.5-S	GACCAACTACGCCGATGA
ClHSP23.5-A	AAGCGATCACGAGAACGAG
ClHSP13.7-S	ACTACTCATTTCCTCCTCGTC
ClHSP13.7-A	GAACCGTAACTCCATCCAC
ClHSP15.3-S	GCCCTCCTATTGGTCTTGT
ClHSP15.3-A	CTGGTCCTGTCATCACTCC
ClHSP16-S	GTCCTCATTCGCCAACAC
ClHSP16-A	GCTGATCTGAAGCACGCC
ClHSP17.6C-S	GCCTAATCTTCCTTCCTCTG
ClHSP17.6C-A	CTCTGCCTTCTTCAACCTC
ClHSP18-S	TTGGCTAATCTTCCTTCCTC
ClHSP18-A	TCTTCTCCTCCTGTTCTTTG
ClHSP18.9A-S	AAAGATGGGAAGAAGATGC
ClHSP18.9A-A	GTTAGTTTACCGTCAGAGGC
ClHSP18.9B-S	CTCGTGCTCAATCGCTTC
ClHSP18.9B-A	ATTCCATCAGTTTCCGCTAC
ClHSP21.8-S	GCACCATTCTCACCCTCTAC
ClHSP21.8-A	GATGCGATTTCAAACTCC
ClHSP23.1B-S	CTATGGTGAAGGTTGGGC
ClHSP23.1B-A	GAGAAACACGGAAGAAGTATGAG
ClHSP27.5-S	GAACACCAATCCCATCAAG
ClHSP27.5-A	CAAAGGTTAAGACCCAAATC
ClHSP27.9-S	ATCGGCAAACGCTCAAATC
ClHSP27.9-A	GTCCCAGAACTCAGCAAATACTC
ClHSP39.8-S	CTTCGCCTCCAACTTCC
ClHSP39.8-A	CCCACAATCCGATCACC
ClHSP42.6-S	ACTGTTCCGACCGACTGC
ClHSP42.6-A	GATCATCATCTTCTCGCTTTG
ClHSP43.7-S	GACTCATACTTGTTTCGTGTG
ClHSP43.7-A	TCGTAACTCCCTGTATCACC
ClHSP50.3-S	CAGCAGATGACATTGTGATAAC
ClHSP50.3-A	CTTGAACCTCCTTGTCTCTG
ClHSP55.8-S	AGAAATCTCCCAAACACCG
ClHSP55.8-A	TCTTCCTCTTGCTGCTGAC
