# Supplementary material for: Genome-Wide Analysis of Watermelon HSP20s and Their Expression Profiles and Subcellular Locations under Stresses
Source: Int J Mol Sci. 2018 Dec 20;20(1):12. doi: 10.3390/ijms20010012 (PMC6337729; doi:10.3390/ijms20010012)
Supplement: Supplementary file 1 [file ijms-20-00012-s001.zip › Table S6 The response pattern of ClHSP20 to hormones and stress.pdf]

Table S6. The expression patterns of watermelon HSP20 genes in response to various hormone (abscisic acid (**A**) and melatonin (**B**)) and stresses (heat (**C**) and CGMMV (**D**)). The second true leaves were collected at 0, 1, 4, and 8 h after the onset of treatments. Asterisks on the top of bars (SE values) indicate statistically significant difference between the compared pairs (* p< 0.05; ** p < 0.01).

(A)

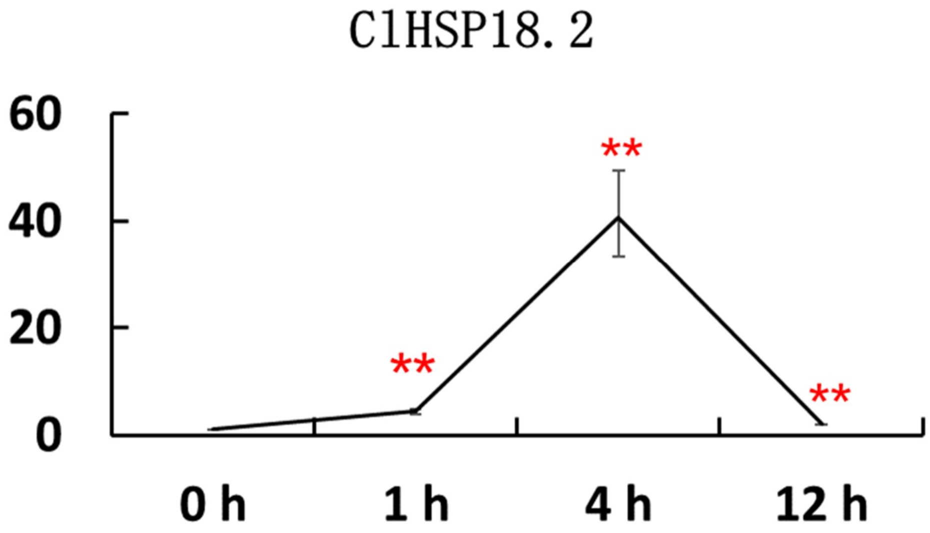

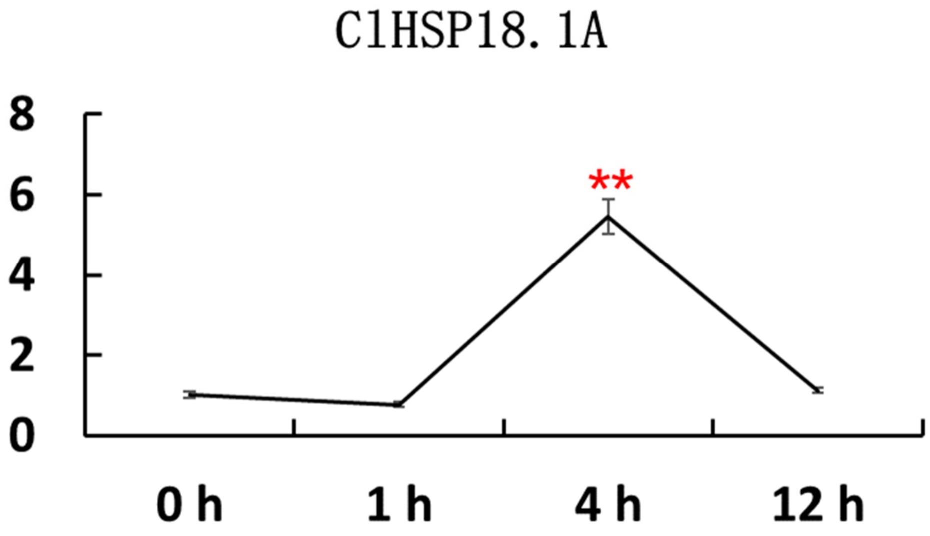

ClHSP23

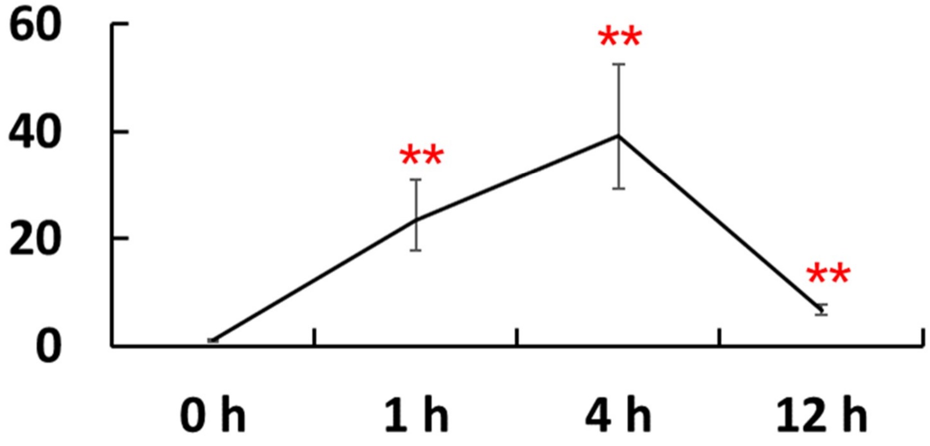

ClHSP21.6

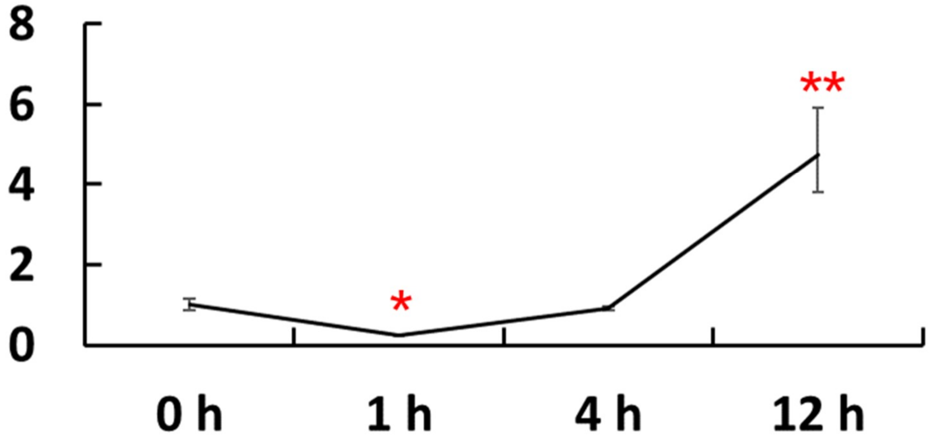

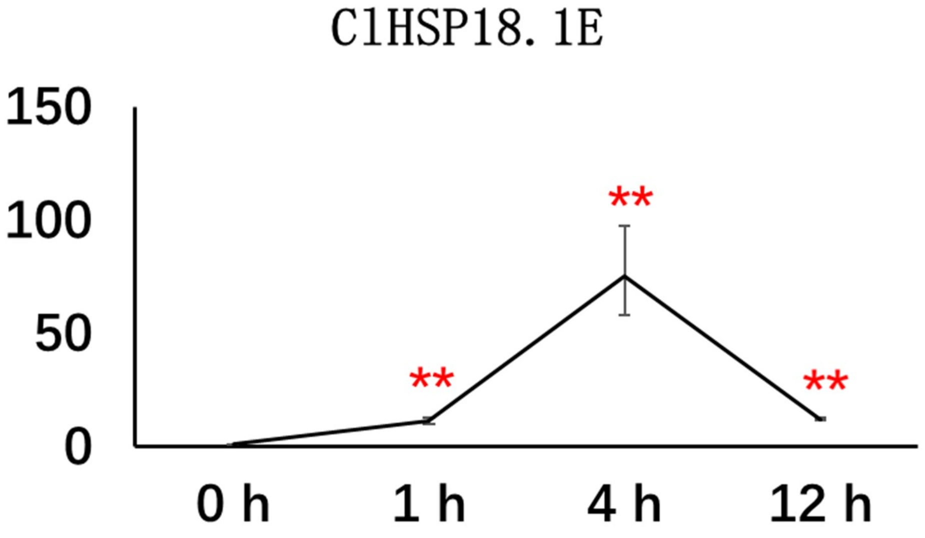
ClHSP18.1E
150
100
50
0
**
**
**
0 h
1 h
4 h
12 h

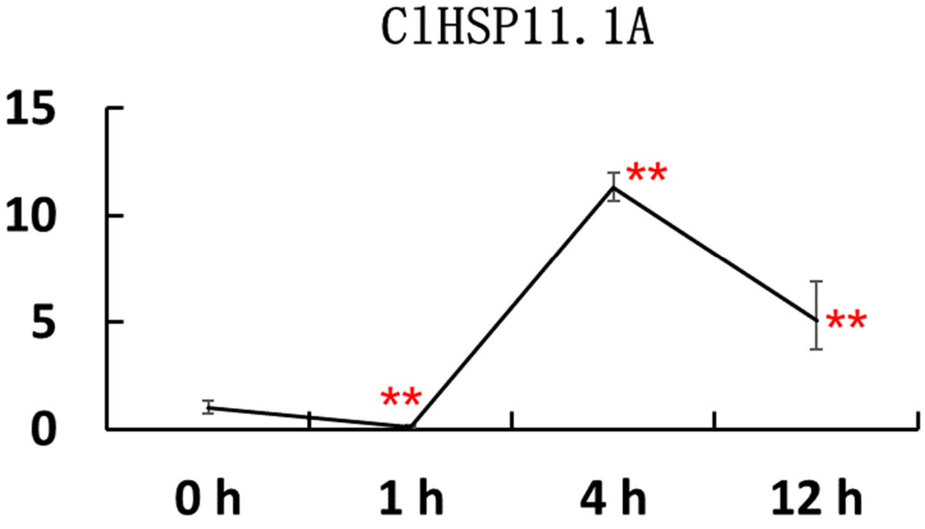
ClHSP11.1A
15
10
5
0
**
**
**
0 h
1 h
4 h
12 h

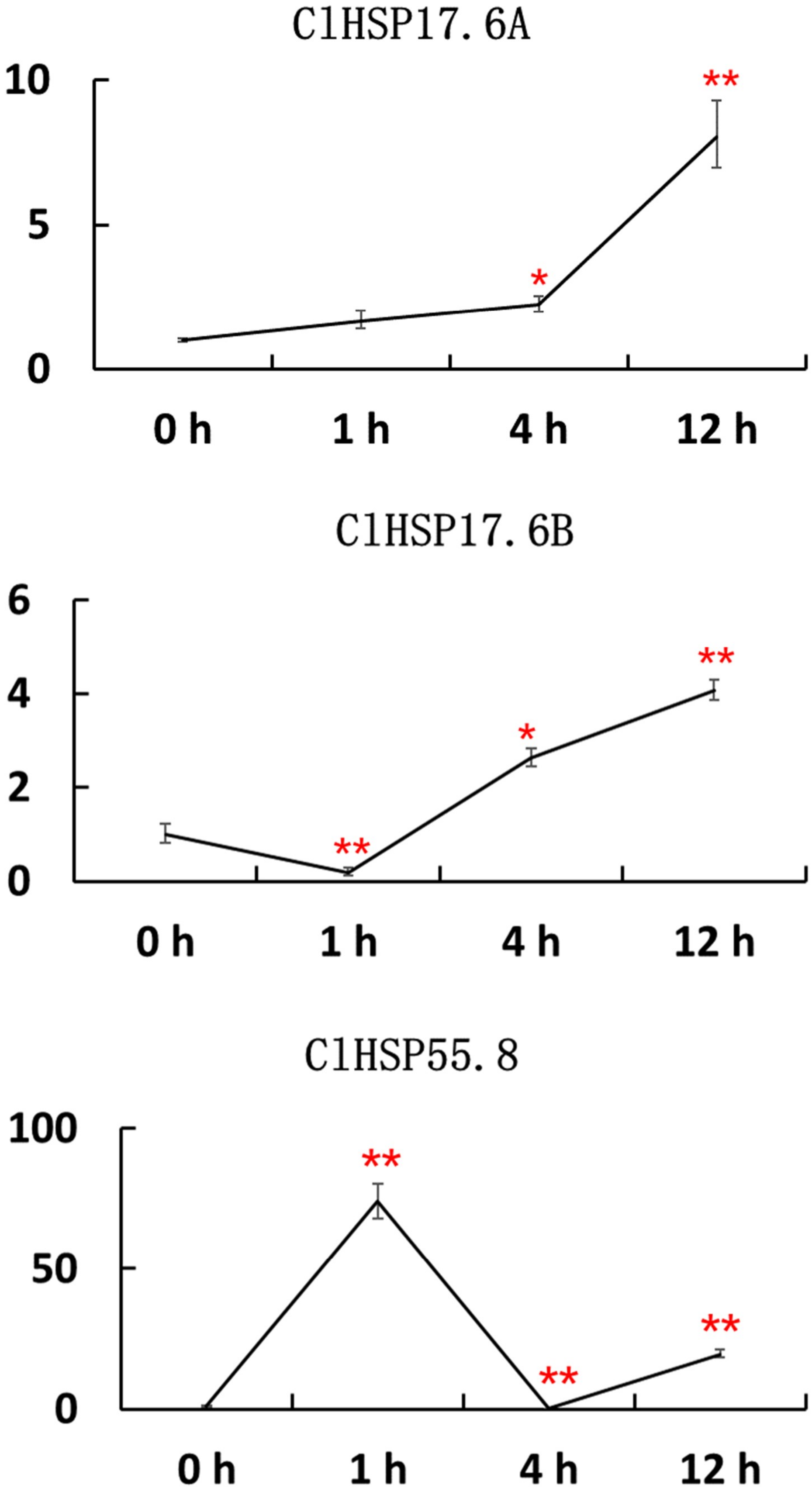

ClHSP17.6A
10
5
0
**
*
0 h
1 h
4 h
12 h
ClHSP17.6B
6
4
2
0
**
*
**
0 h
1 h
4 h
12 h
ClHSP55.8
100
50
0
**
**
**
0 h
1 h
4 h
12 h

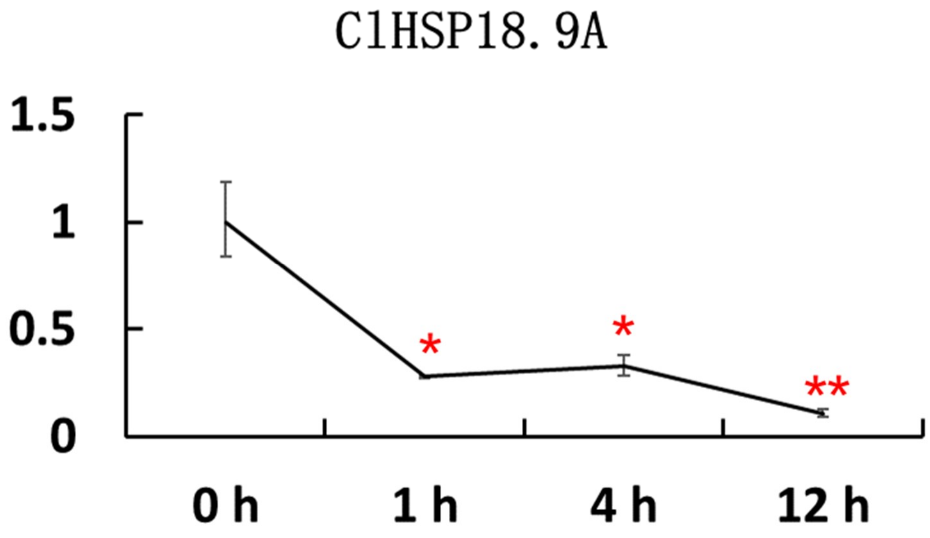
ClHSP18.9A
1.5
1
0.5
0
*
*
**
0 h
1 h
4 h
12 h

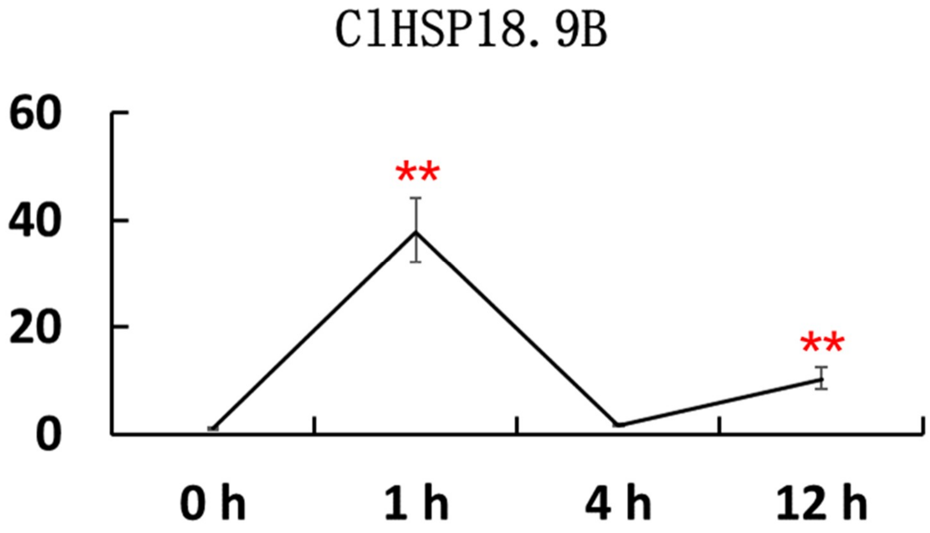
ClHSP18.9B
60
40
20
0
**
**
0 h
1 h
4 h
12 h

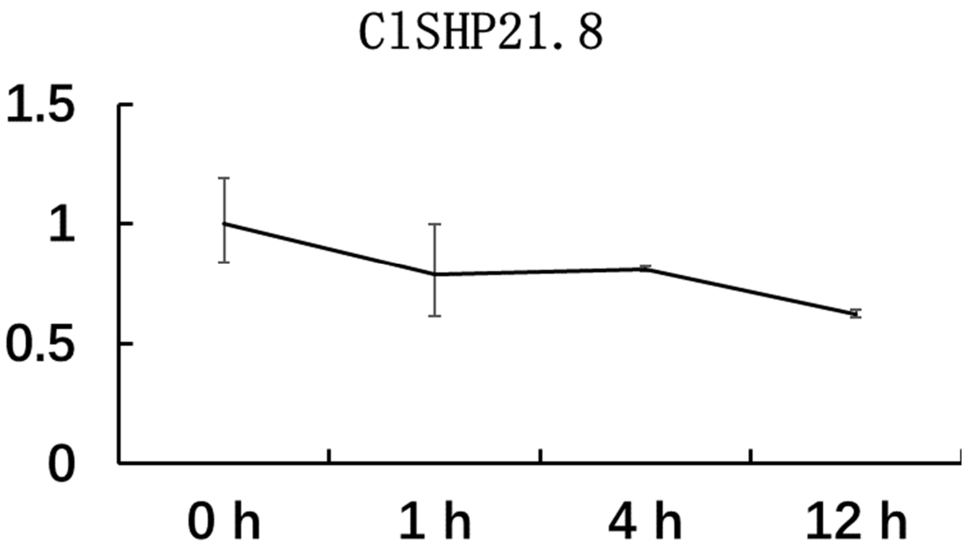
ClSHP21.8
1.5
1
0.5
0
0 h
1 h
4 h
12 h

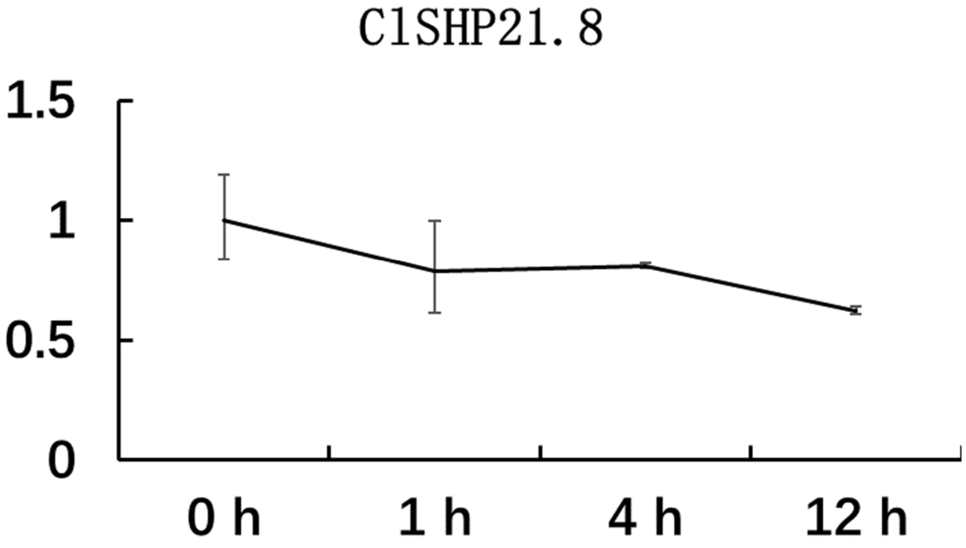
ClSHP21.8
1.5
1
0.5
0
0 h
1 h
4 h
12 h

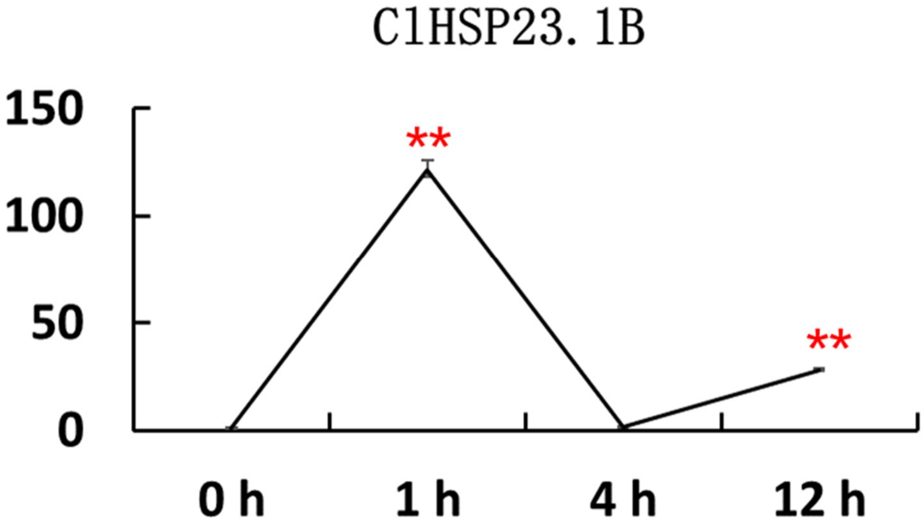
ClHSP23.1B
150
100
50
0
**
**
0 h
1 h
4 h
12 h

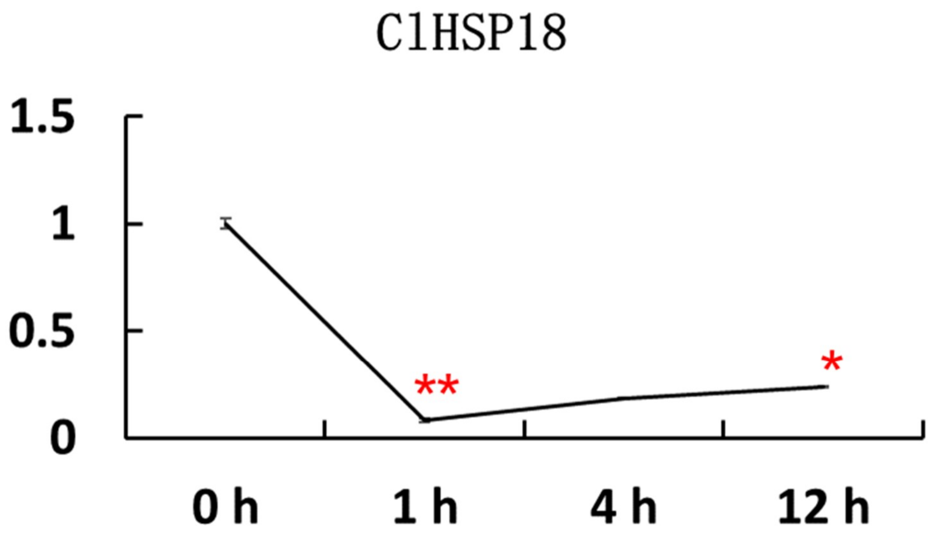
ClHSP18
1.5
1
0.5
0
**
*
0 h
1 h
4 h
12 h

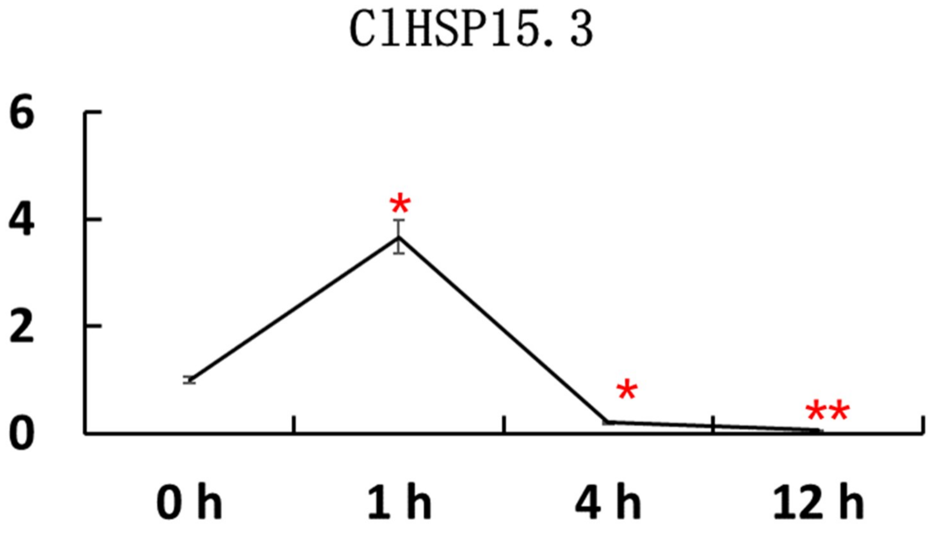
ClHSP15.3
6
4
2
0
*
*
**
0 h
1 h
4 h
12 h

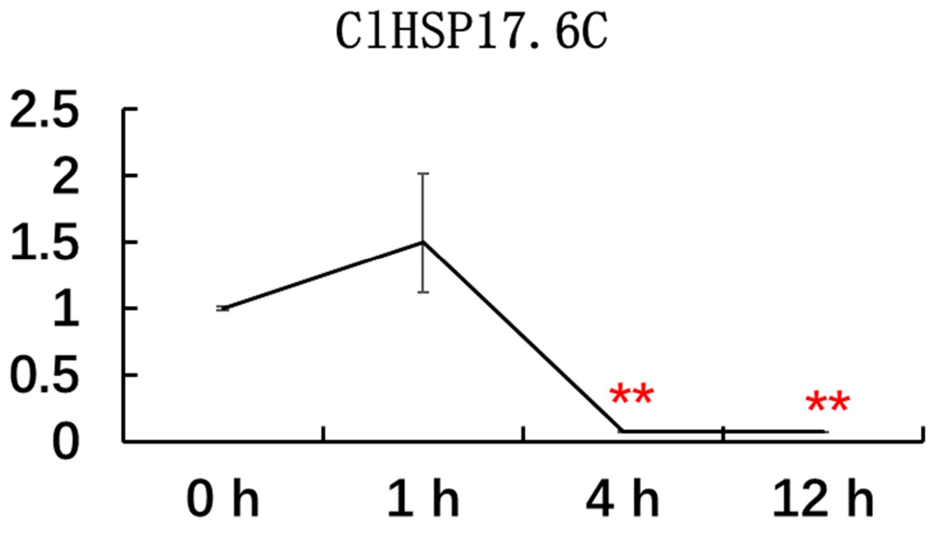
ClHSP17.6C
2.5
2
1.5
1
0.5
0
**
**
0 h
1 h
4 h
12 h

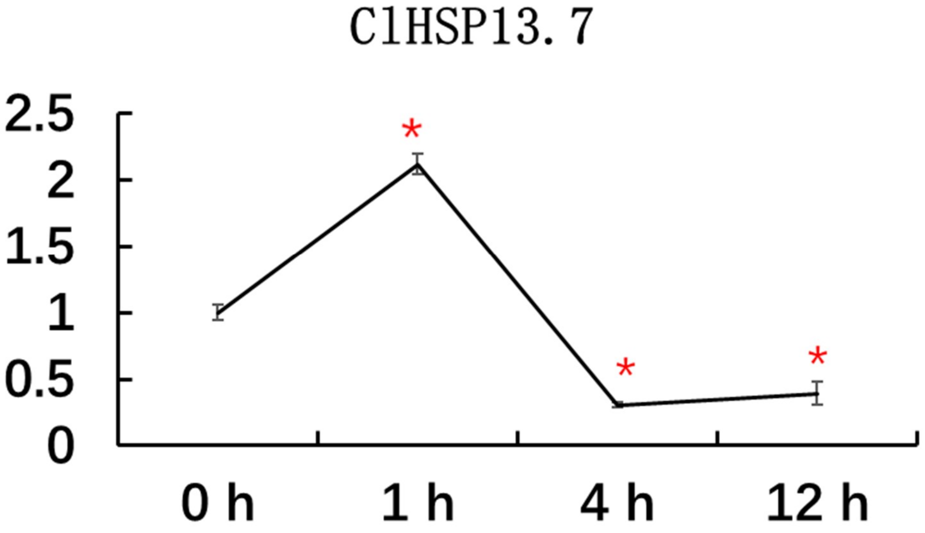
ClHSP13.7
2.5
2
1.5
1
0.5
0
*
*
*
0 h
1 h
4 h
12 h

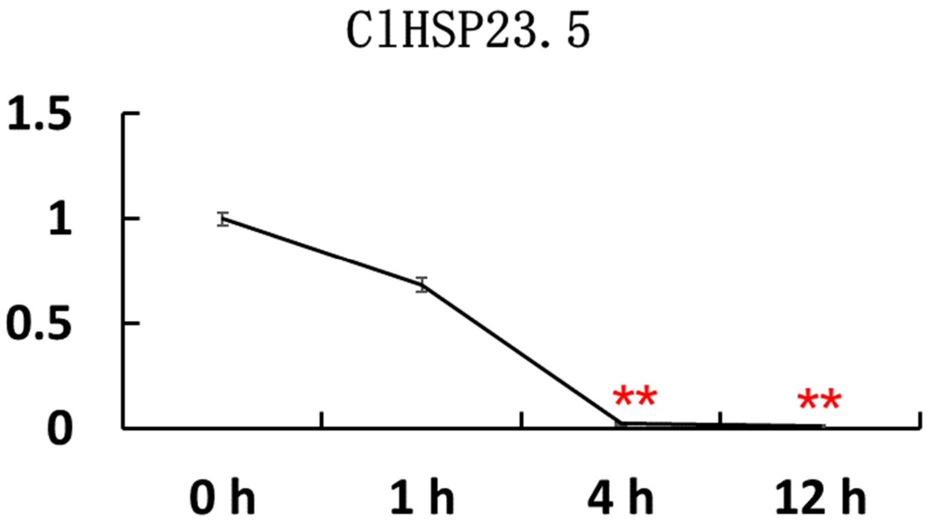
ClHSP23.5
1.5
1
0.5
0
**
**
0 h
1 h
4 h
12 h

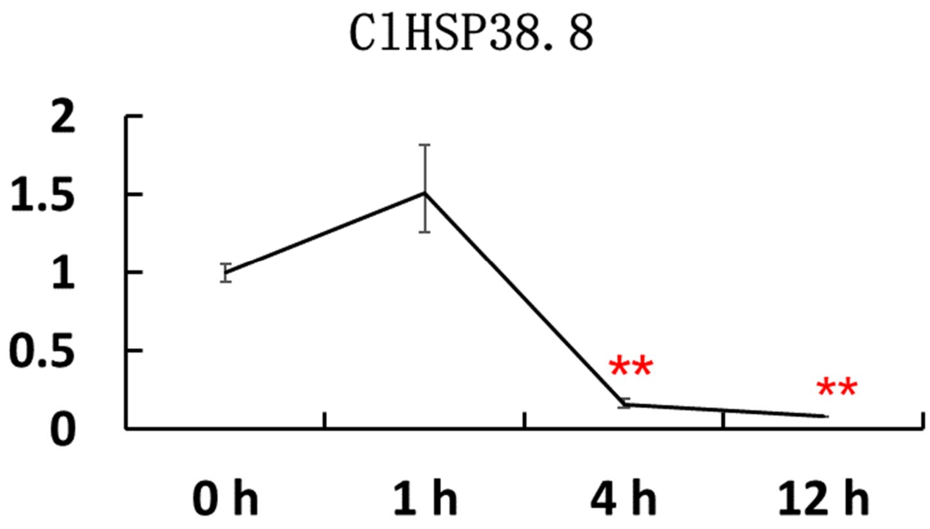
ClHSP38.8
2
1.5
1
0.5
0
**
**
0 h
1 h
4 h
12 h

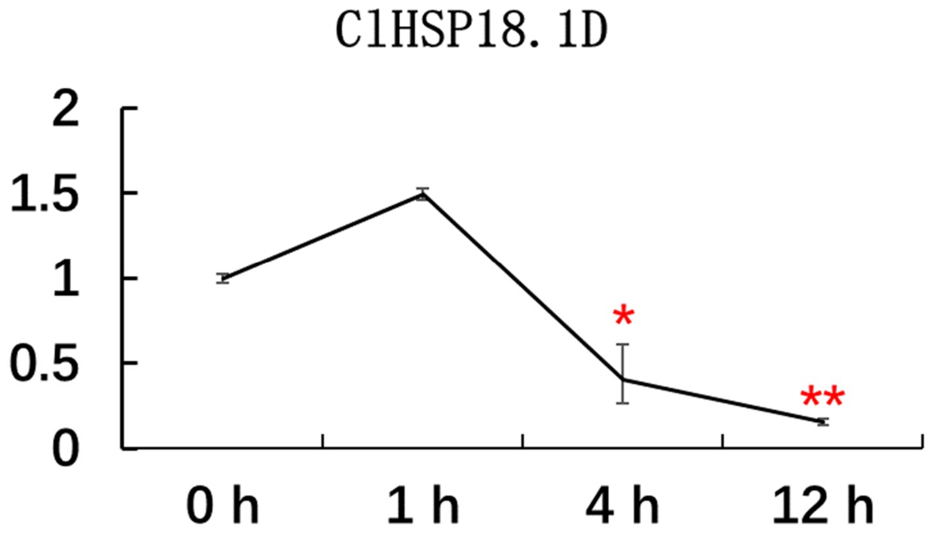
ClHSP18.1D
2
1.5
1
0.5
0
*
**
0 h
1 h
4 h
12 h

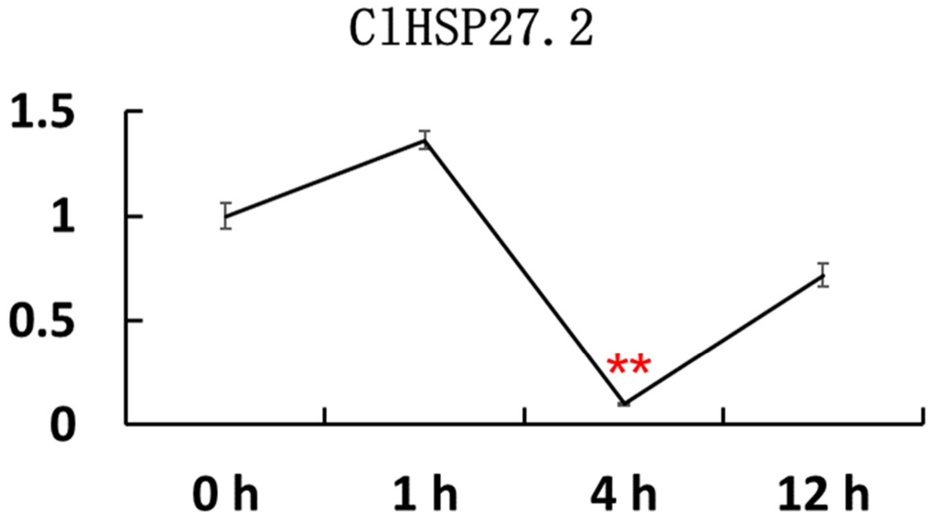
ClHSP27.2
1.5
1
0.5
0
**
0 h
1 h
4 h
12 h

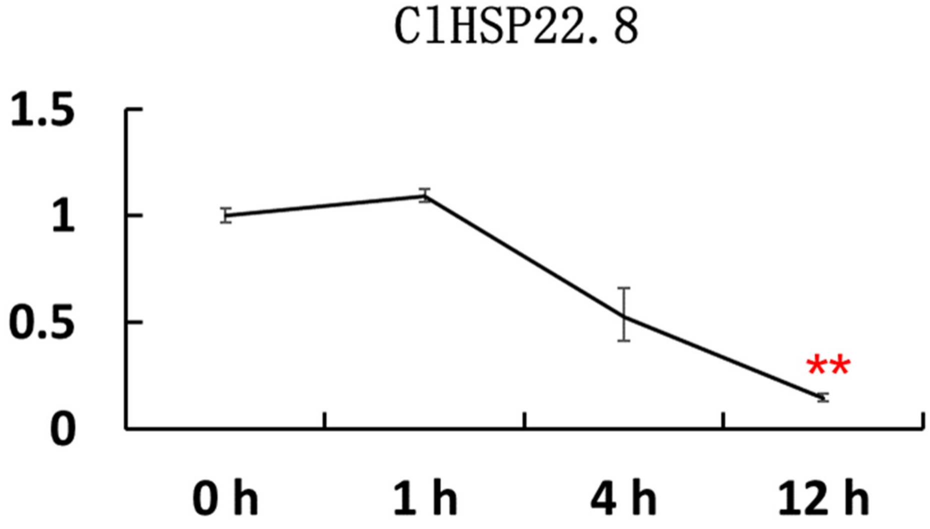
ClHSP22.8
1.5
1
0.5
0
**
0 h
1 h
4 h
12 h

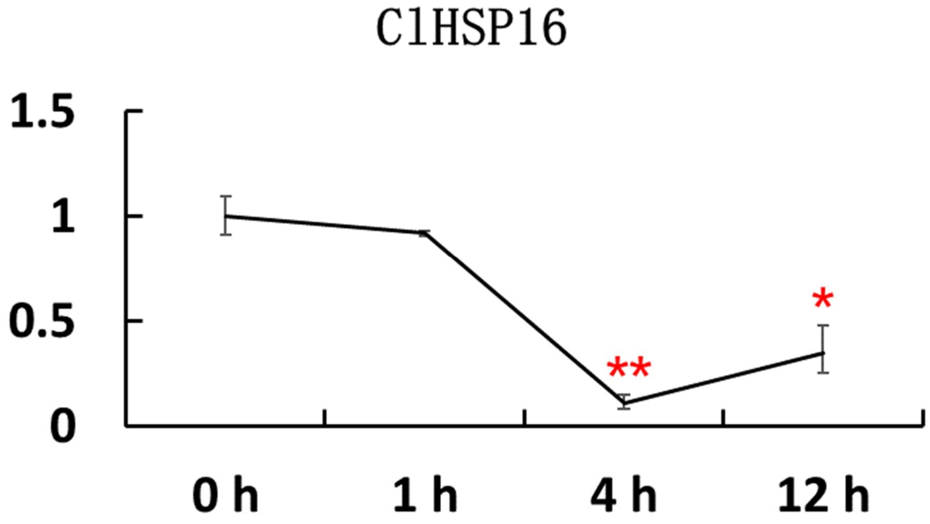
ClHSP16
1.5
1
0.5
0
**
*
0 h
1 h
4 h
12 h

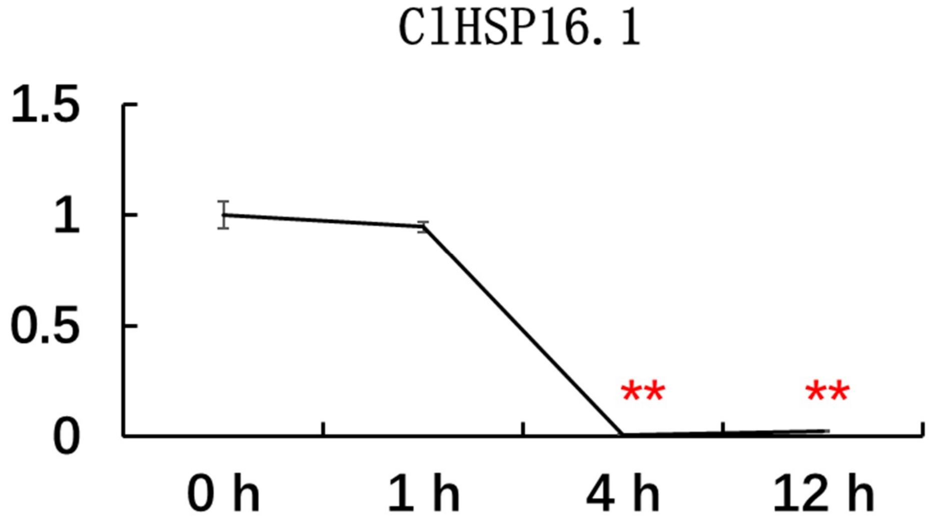
ClHSP16.1
1.5
1
0.5
0
**
**
0 h
1 h
4 h
12 h

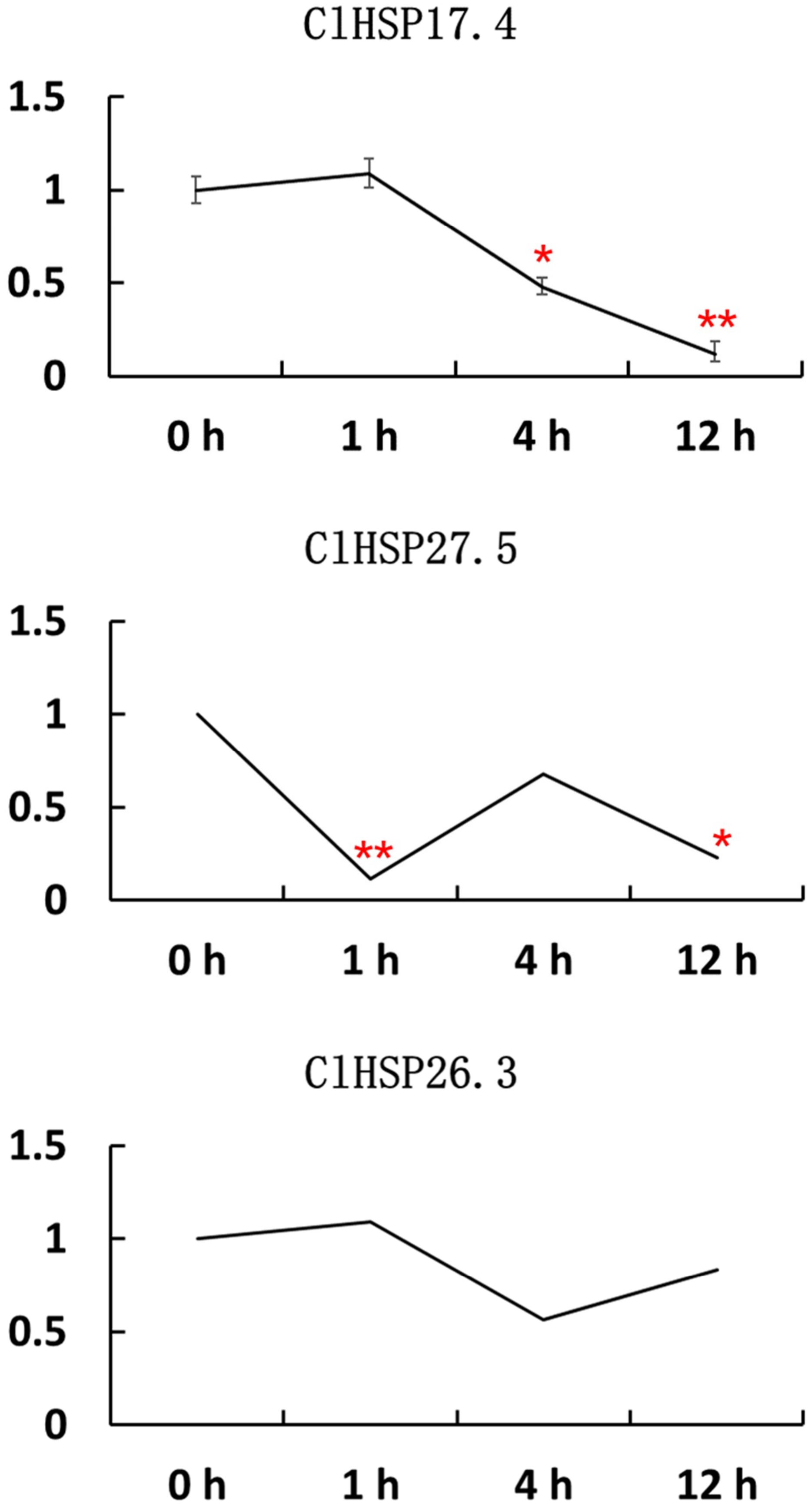
ClHSP17.4
1.5
1
0.5
0
0 h
1 h
4 h
12 h
*
**
ClHSP27.5
1.5
1
0.5
0
0 h
1 h
4 h
12 h
**
*
ClHSP26.3
1.5
1
0.5
0
0 h
1 h
4 h
12 h

ClHSP27.9

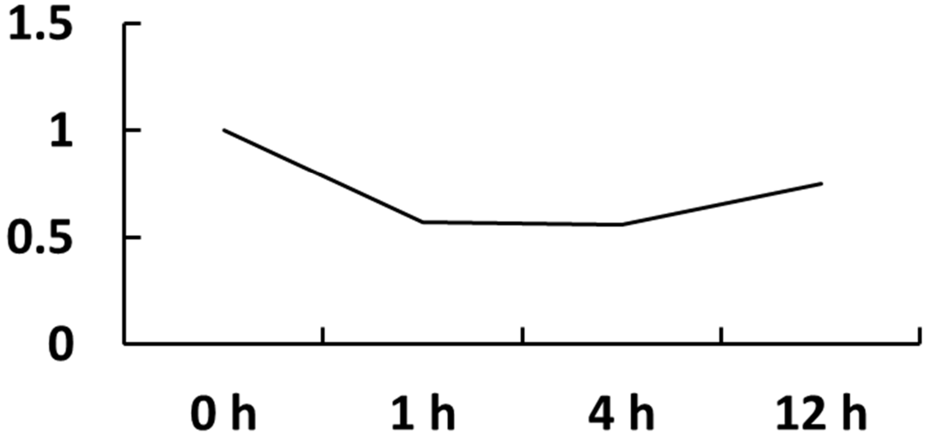

ClHSP39.8

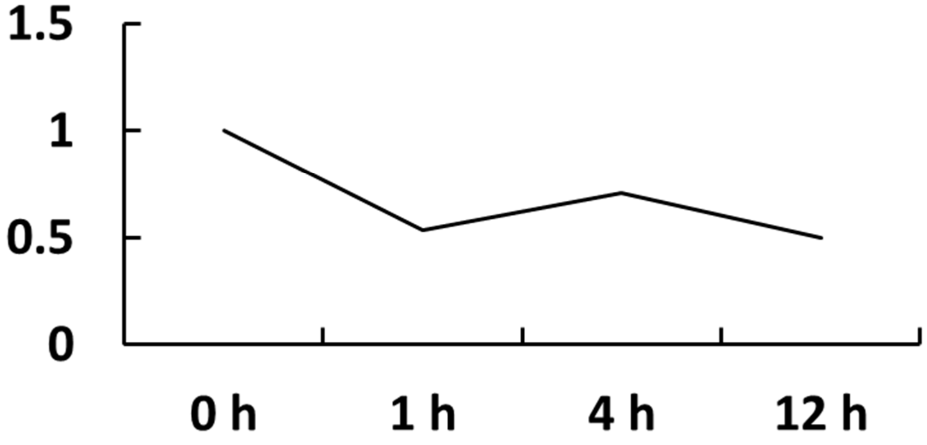

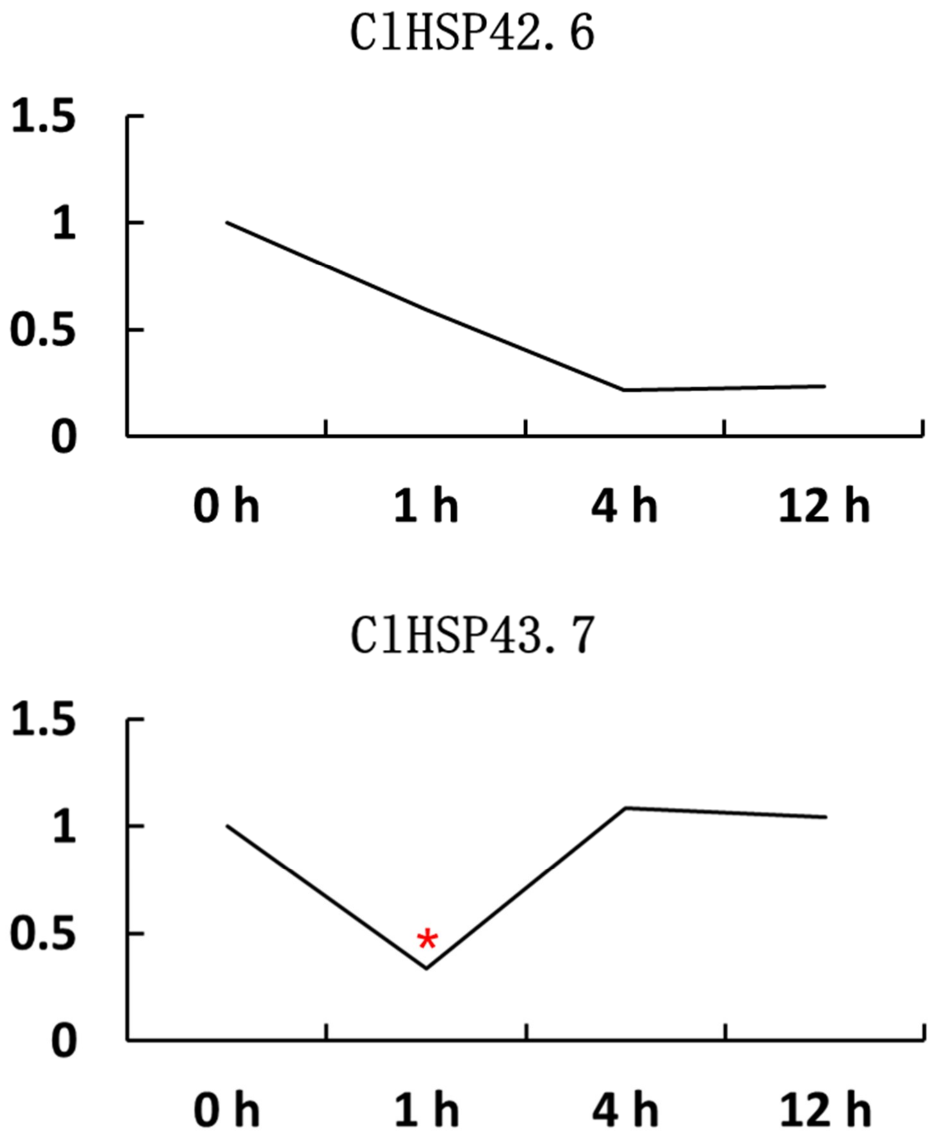

(B)

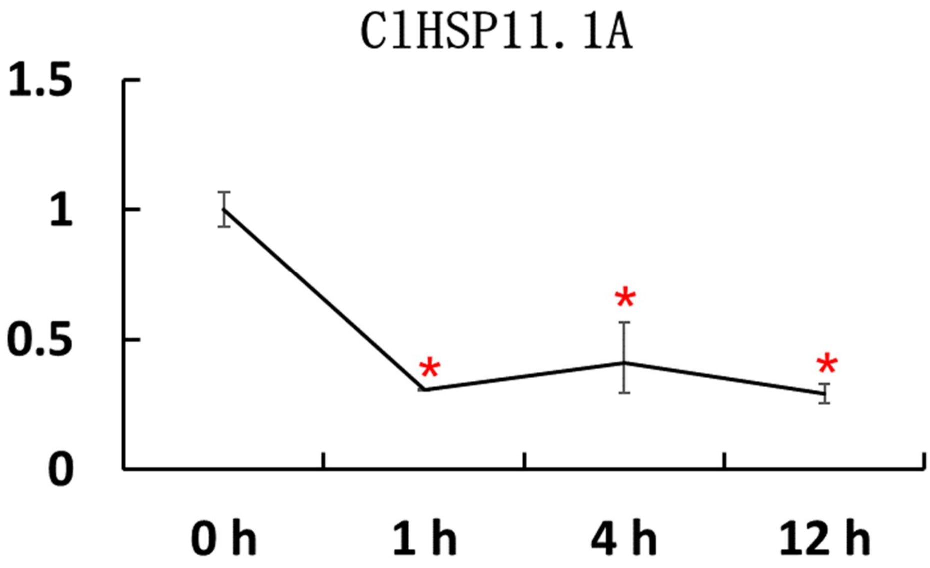
ClHSP11.1A
1.5
1
0.5
0
*
*
*
0 h
1 h
4 h
12 h

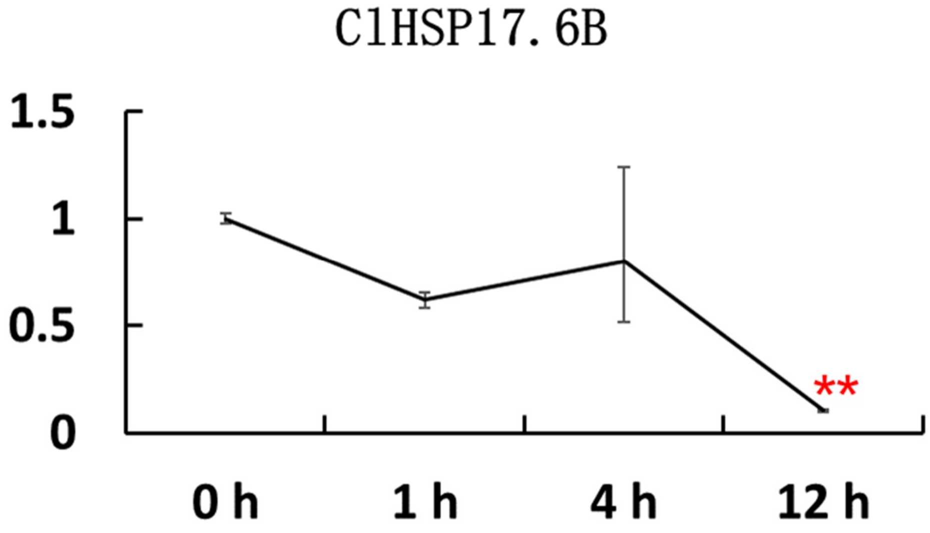
ClHSP17.6B
1.5
1
0.5
0
**
0 h
1 h
4 h
12 h

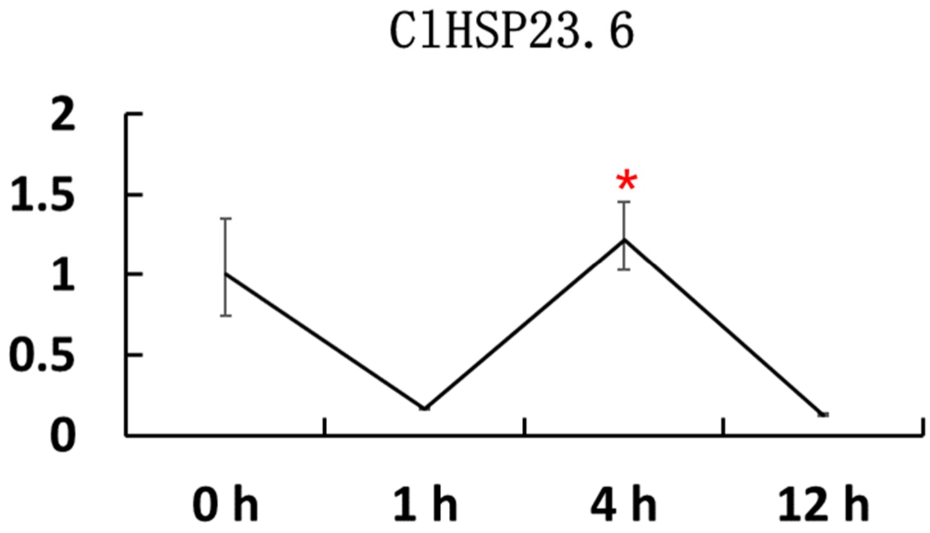
ClHSP23.6
2
1.5
1
0.5
0
*
0 h
1 h
4 h
12 h

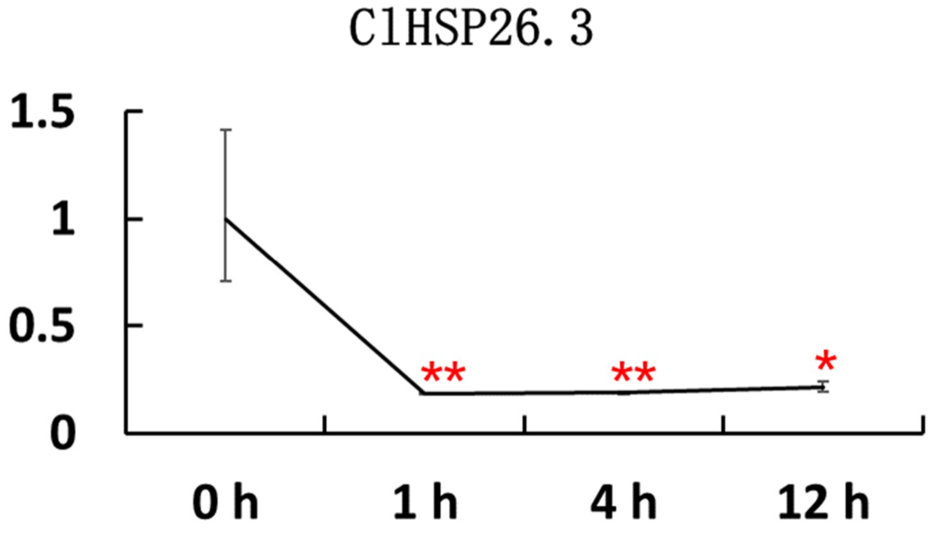
ClHSP26.3
1.5
1
0.5
0
**
**
*
0 h
1 h
4 h
12 h

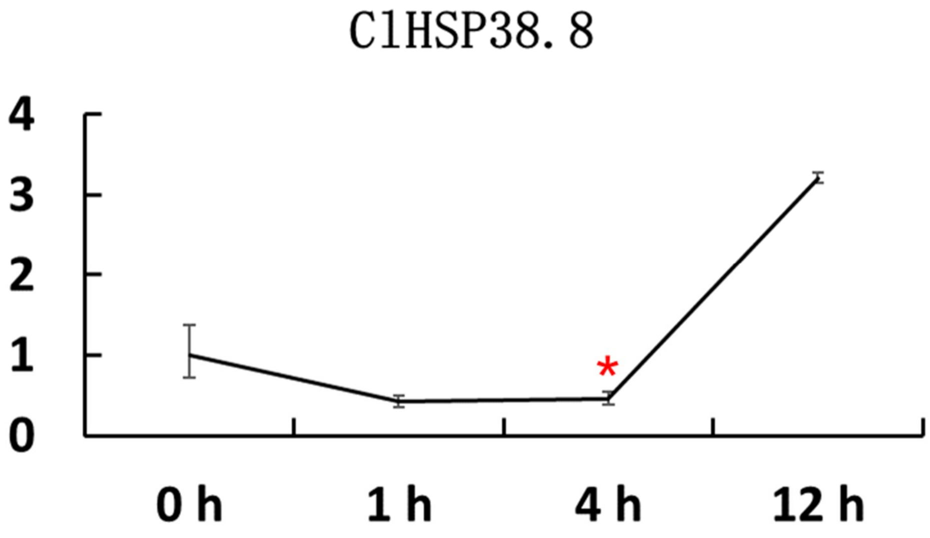
ClHSP38.8
4
3
2
1
0
*
0 h
1 h
4 h
12 h

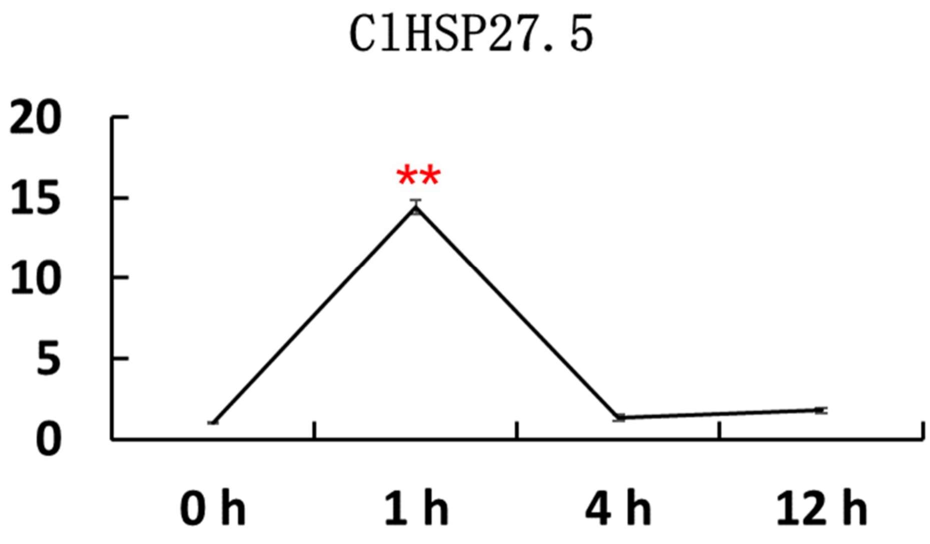
ClHSP27.5
20
15
10
5
0
**
0 h
1 h
4 h
12 h

ClHSP27.9

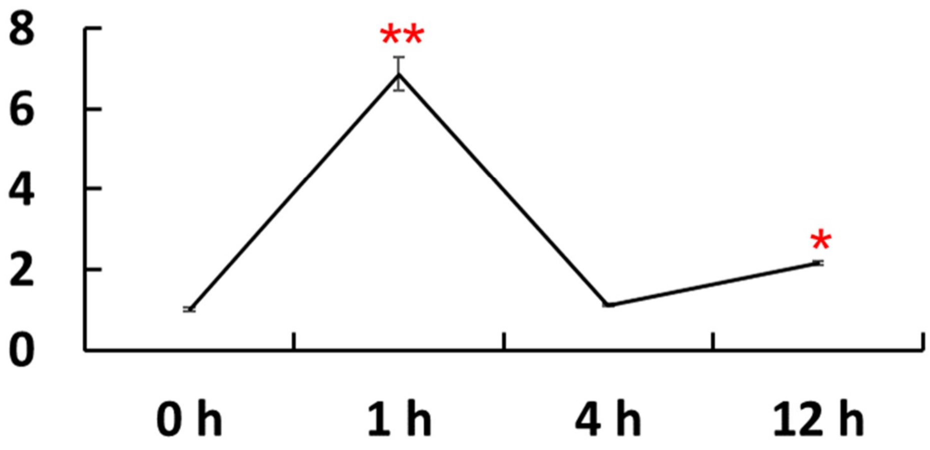

ClHSP39.8

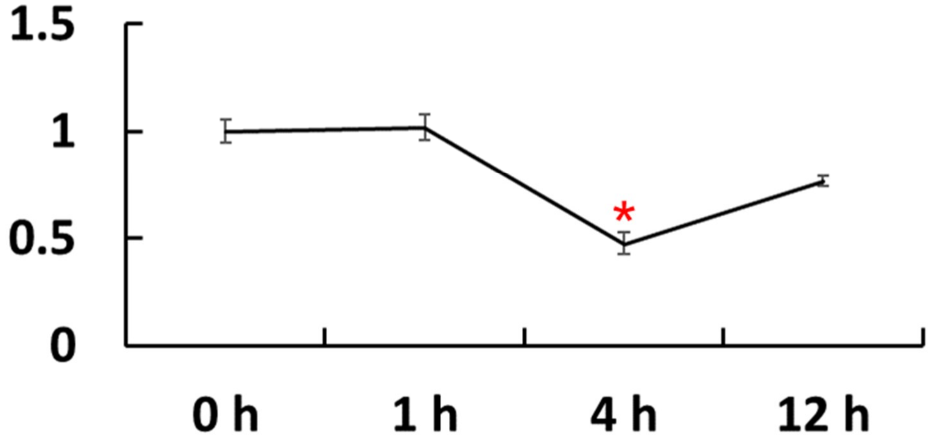

ClHSP42.6

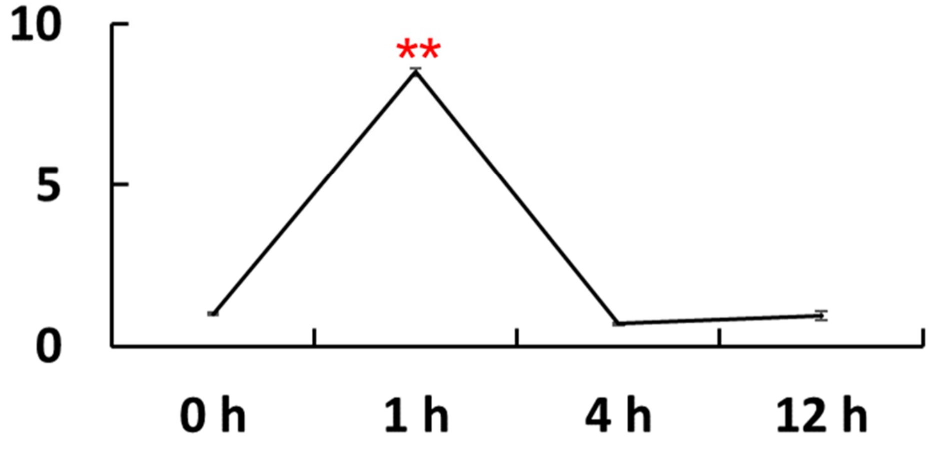

ClHSP43.7

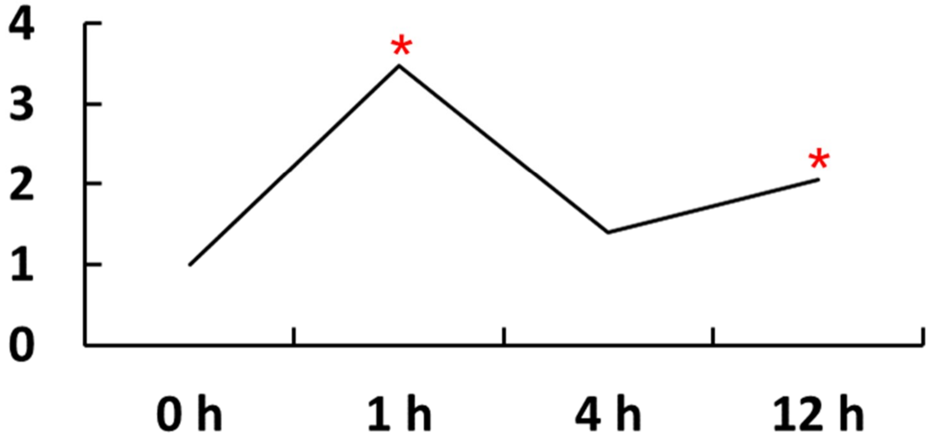

ClHSP50.3

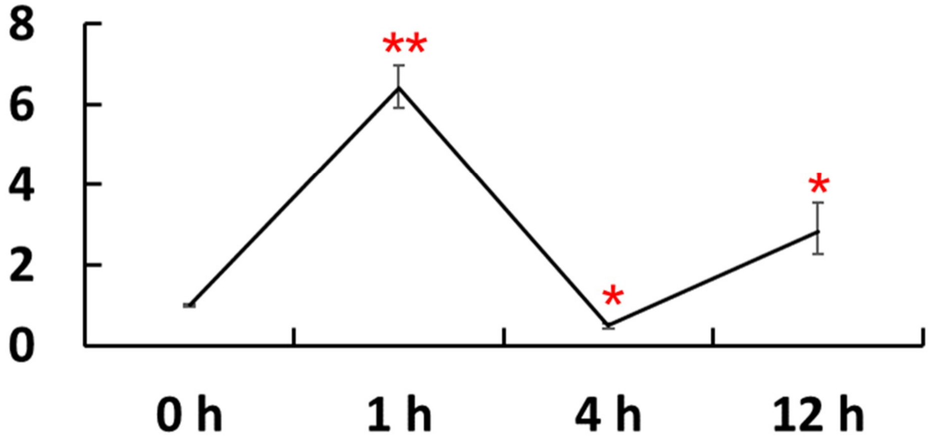

ClHSP55.8

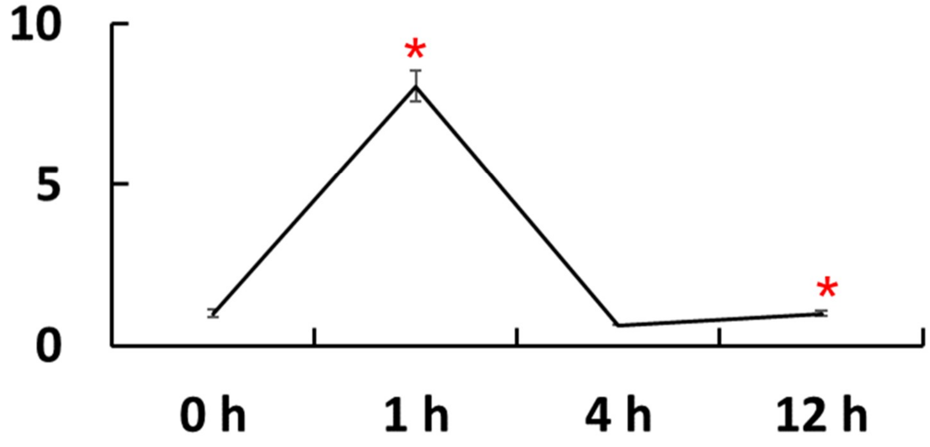

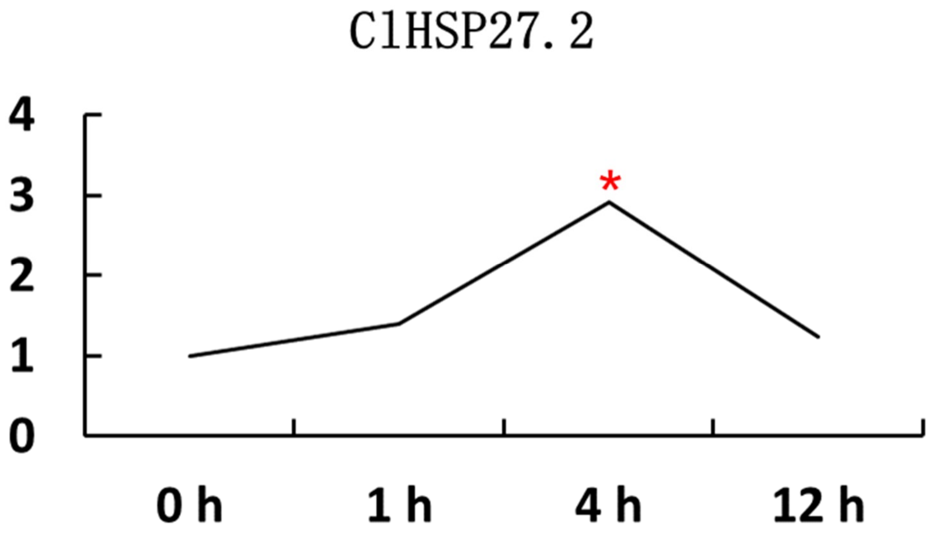
ClHSP27.2
4
3
2
1
0
*
0 h
1 h
4 h
12 h

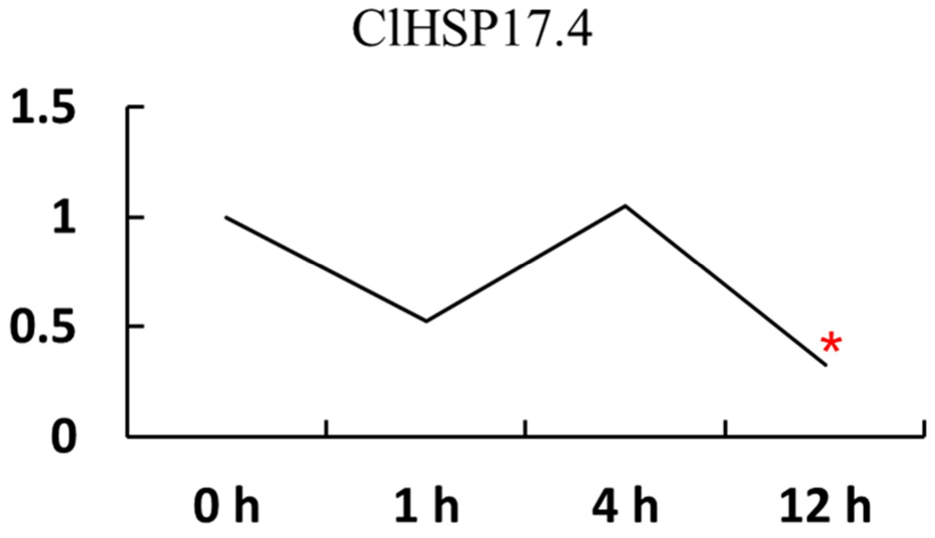
ClHSP17.4
1.5
1
0.5
0
*
0 h
1 h
4 h
12 h

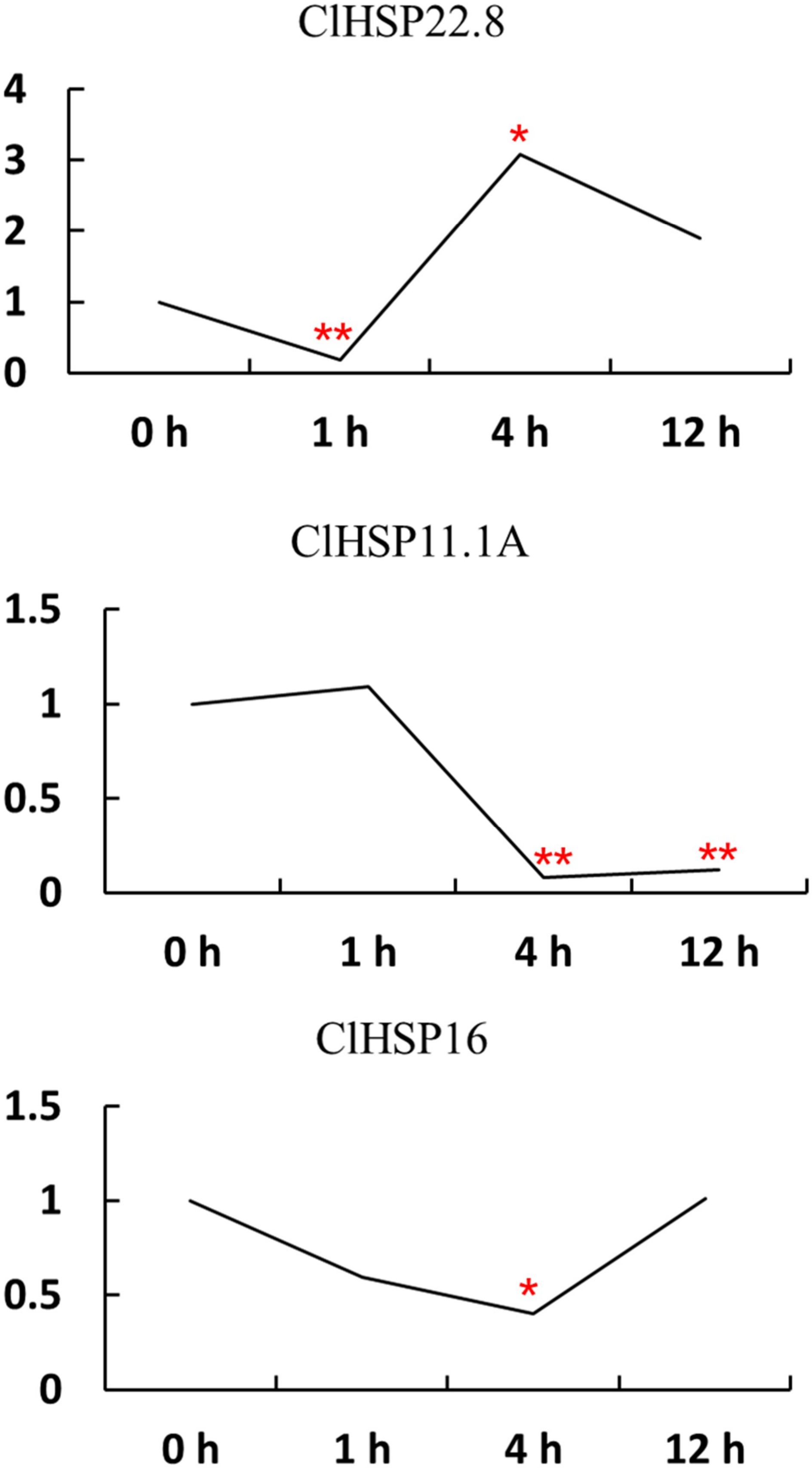
ClHSP22.8
4
3
2
1
0
*
**
0 h
1 h
4 h
12 h
ClHSP11.1A
1.5
1
0.5
0
**
**
0 h
1 h
4 h
12 h
ClHSP16
1.5
1
0.5
0
*
0 h
1 h
4 h
12 h

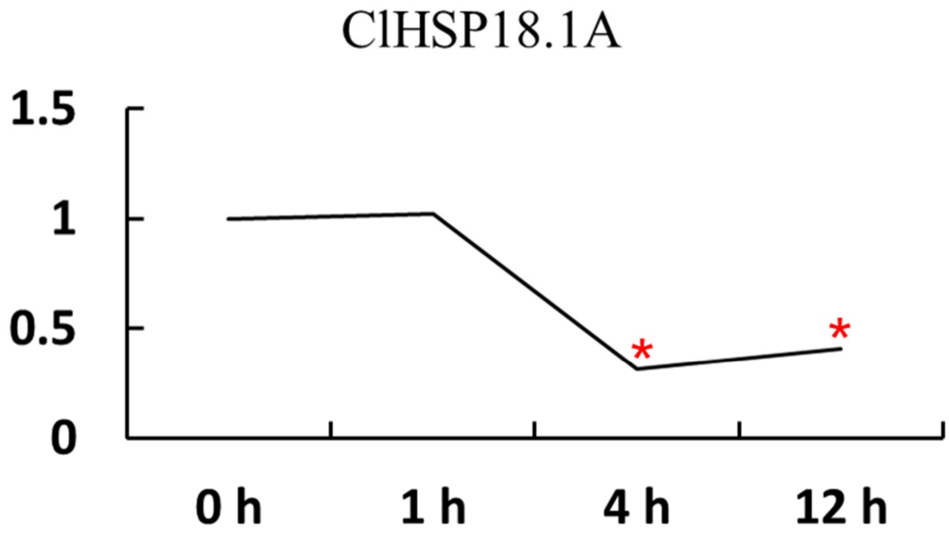
ClHSP18.1A
1.5
1
0.5
0
*
*
0 h
1 h
4 h
12 h

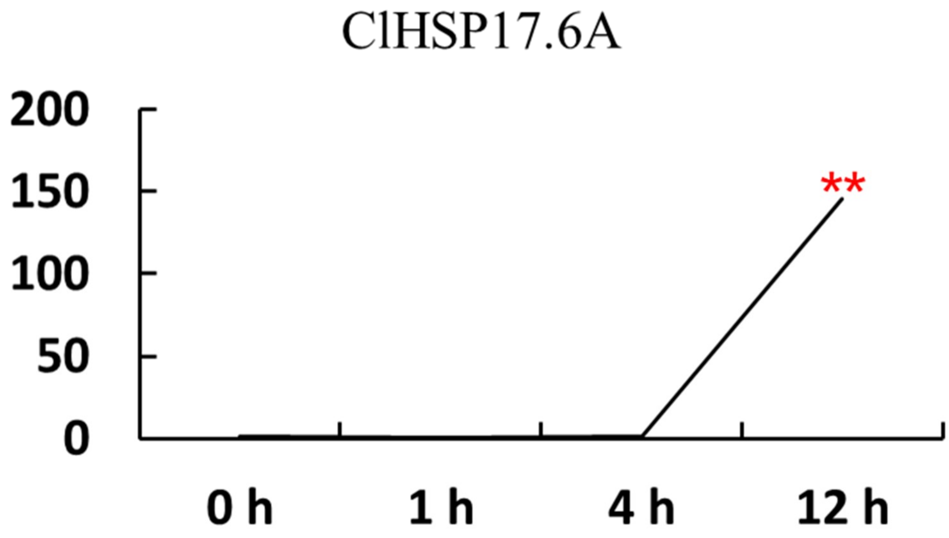
ClHSP17.6A
200
150
100
50
0
**
0 h
1 h
4 h
12 h

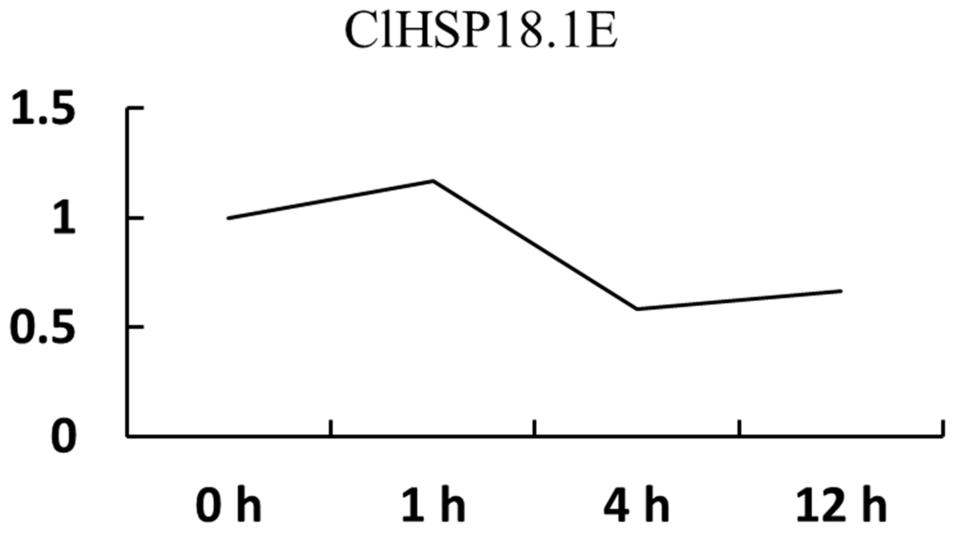
ClHSP18.1E
1.5
1
0.5
0
0 h
1 h
4 h
12 h

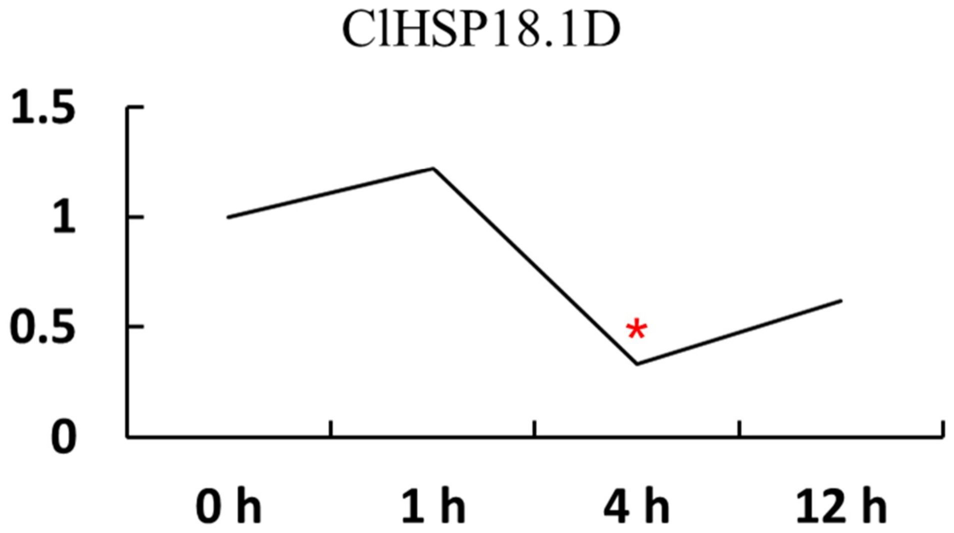
ClHSP18.1D
1.5
1
0.5
0
*
0 h
1 h
4 h
12 h

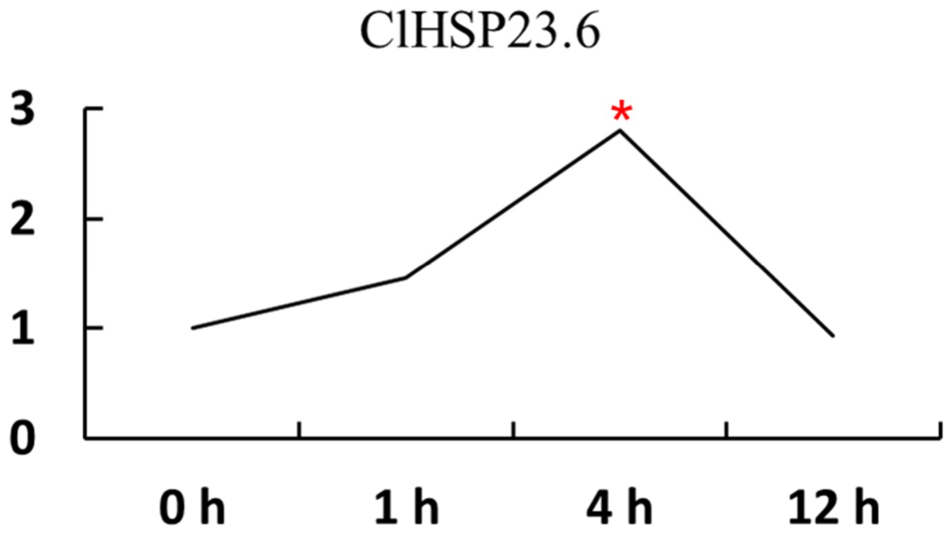
ClHSP23.6
3
2
1
0
*
0 h
1 h
4 h
12 h

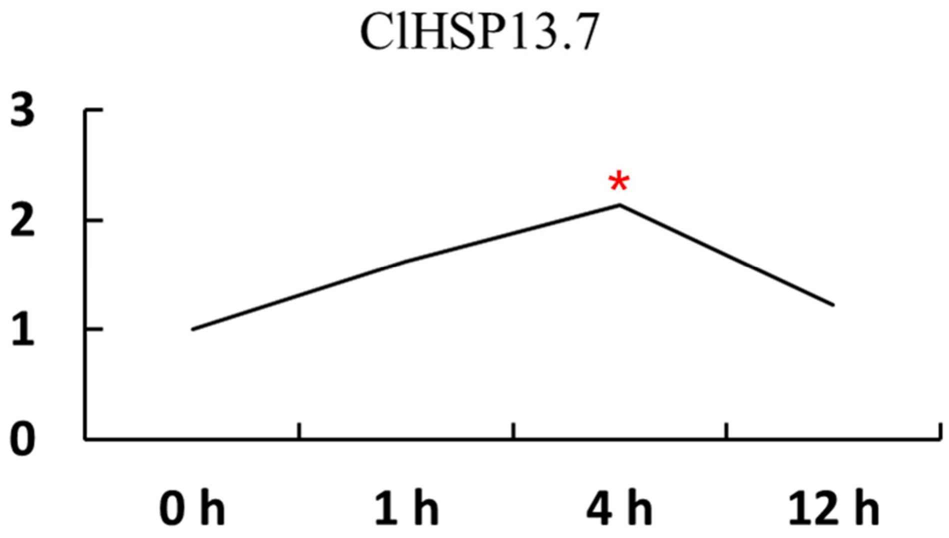
ClHSP13.7
3
2
1
0
*
0 h
1 h
4 h
12 h

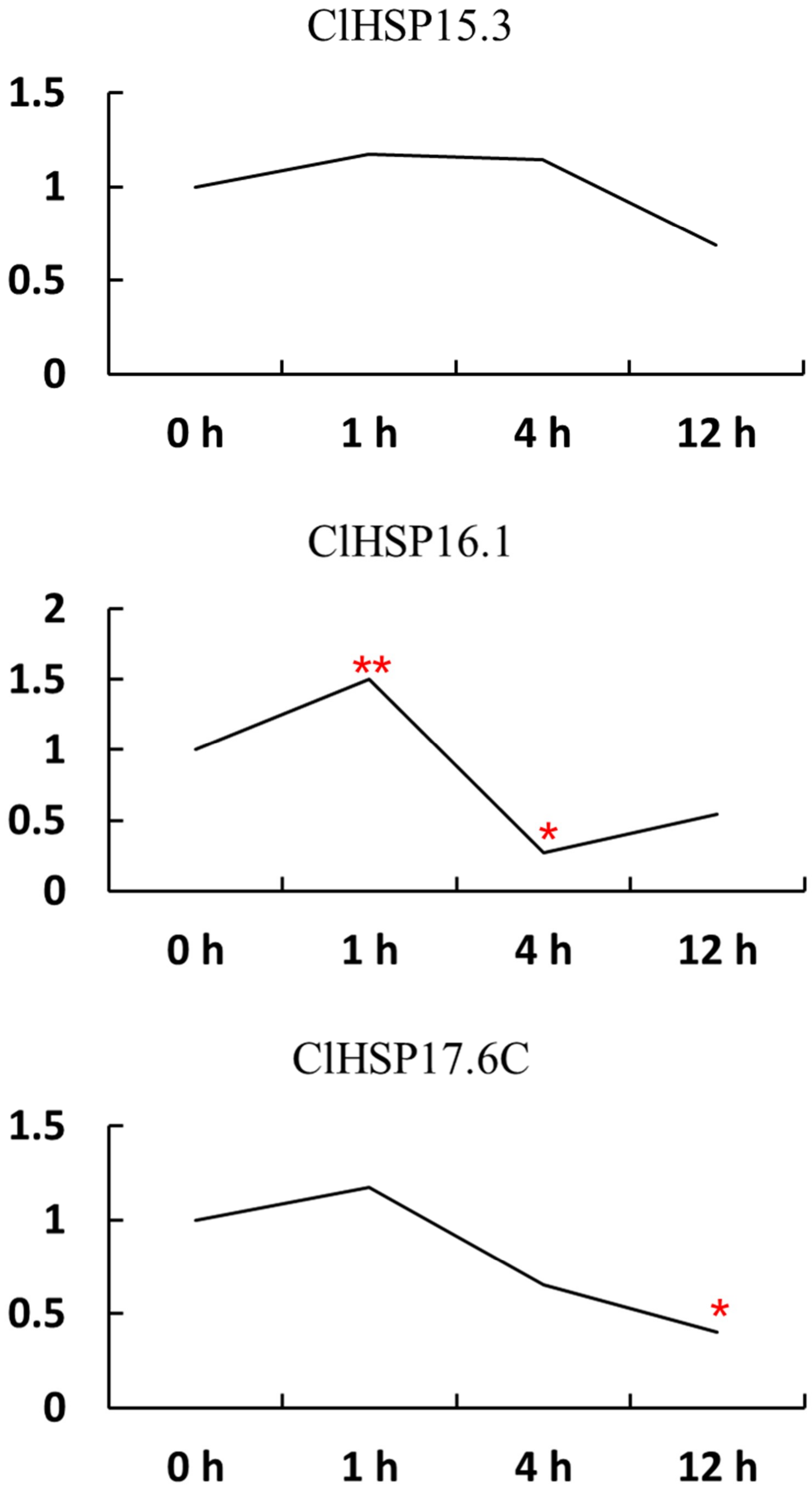
ClHSP15.3
1.5
1
0.5
0
0 h
1 h
4 h
12 h
ClHSP16.1
2
1.5
1
0.5
0
**
*
0 h
1 h
4 h
12 h
ClHSP17.6C
1.5
1
0.5
0
*
0 h
1 h
4 h
12 h

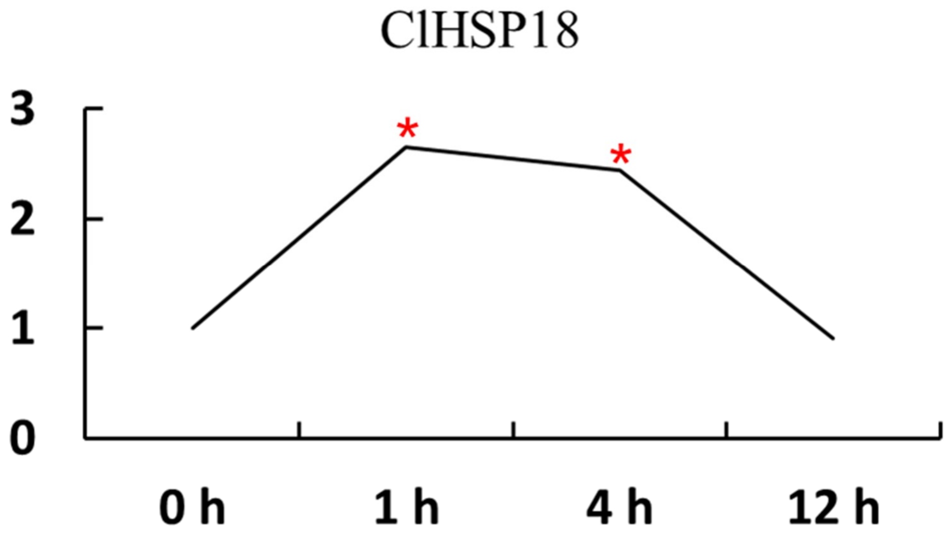
ClHSP18
3
2
1
0
*
*
0 h
1 h
4 h
12 h

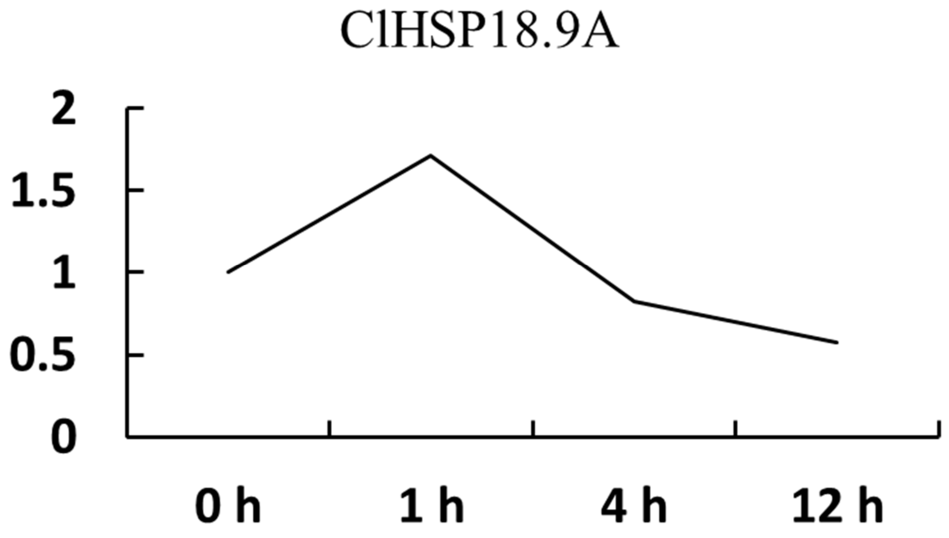
ClHSP18.9A
2
1.5
1
0.5
0
0 h
1 h
4 h
12 h

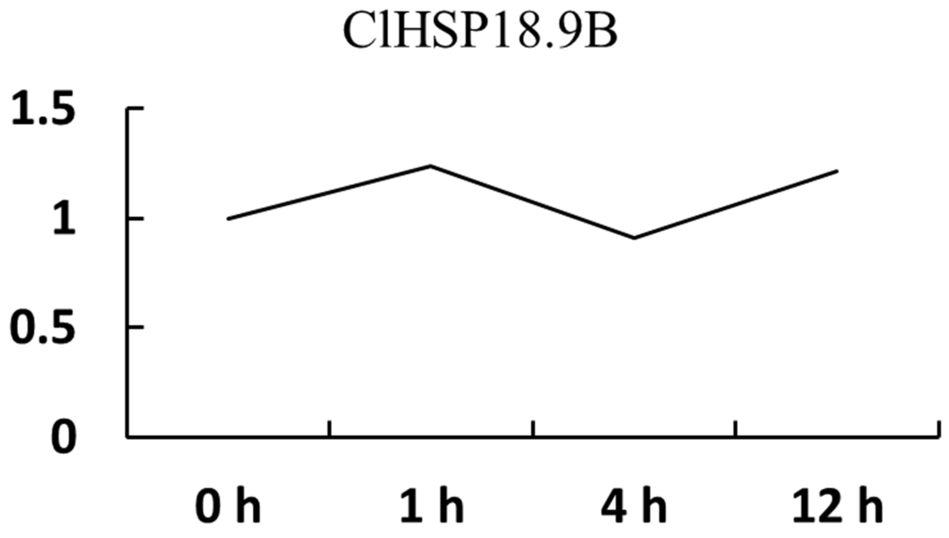
ClHSP18.9B
1.5
1
0.5
0
0 h
1 h
4 h
12 h

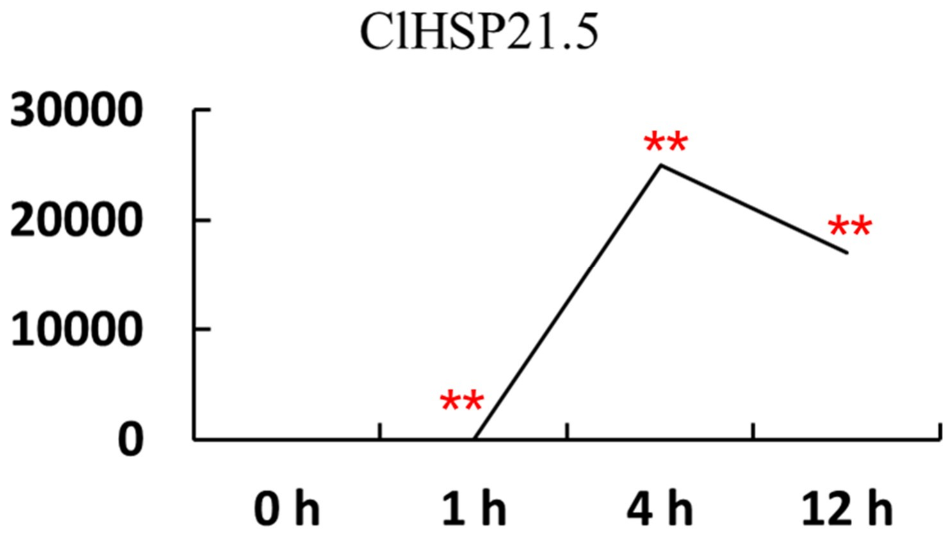
ClHSP21.5
30000
20000
10000
0
**
**
**
0 h
1 h
4 h
12 h

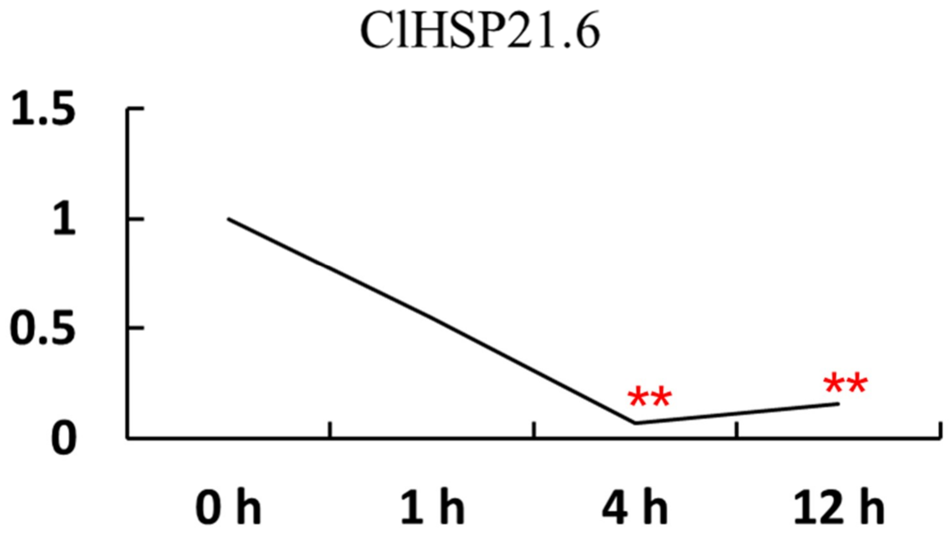
ClHSP21.6
1.5
1
0.5
0
**
**
0 h
1 h
4 h
12 h

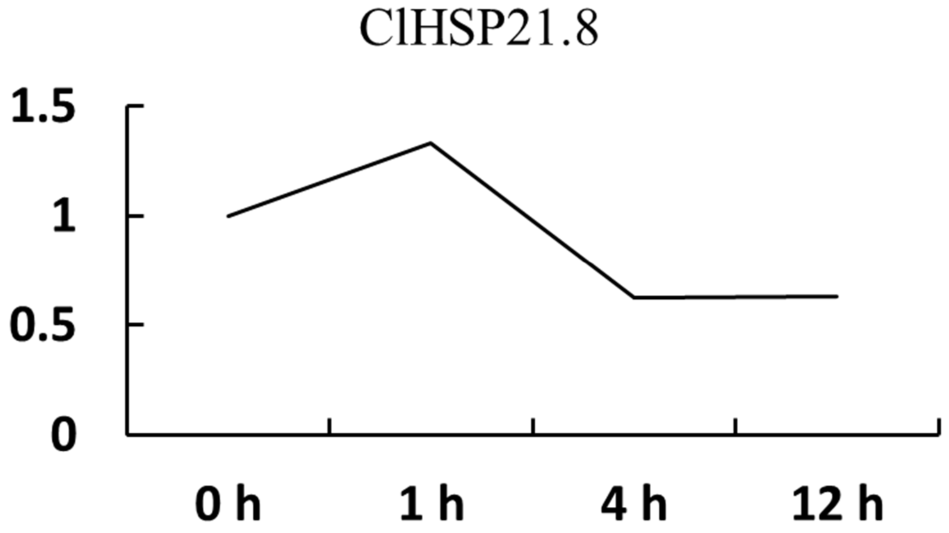
ClHSP21.8
1.5
1
0.5
0
0 h
1 h
4 h
12 h

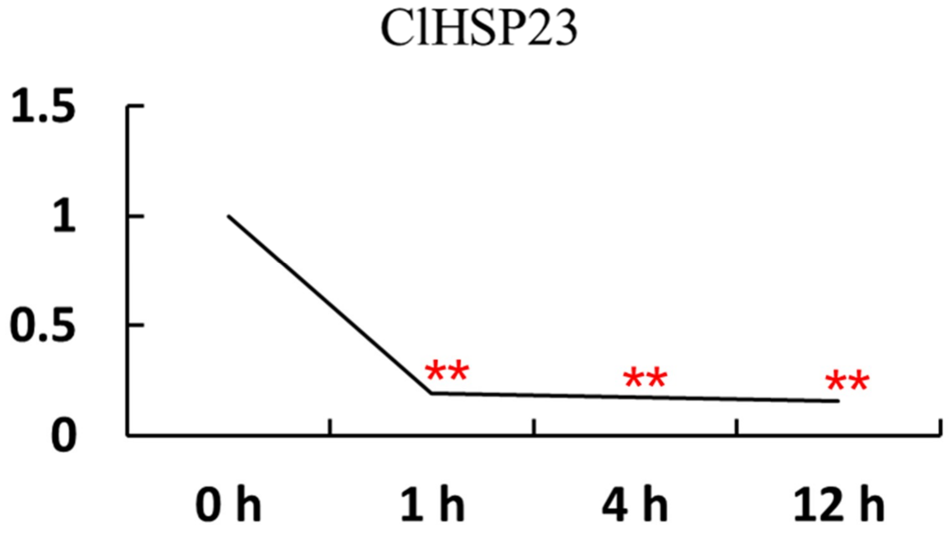
ClHSP23
1.5
1
0.5
0
**
**
**
0 h
1 h
4 h
12 h

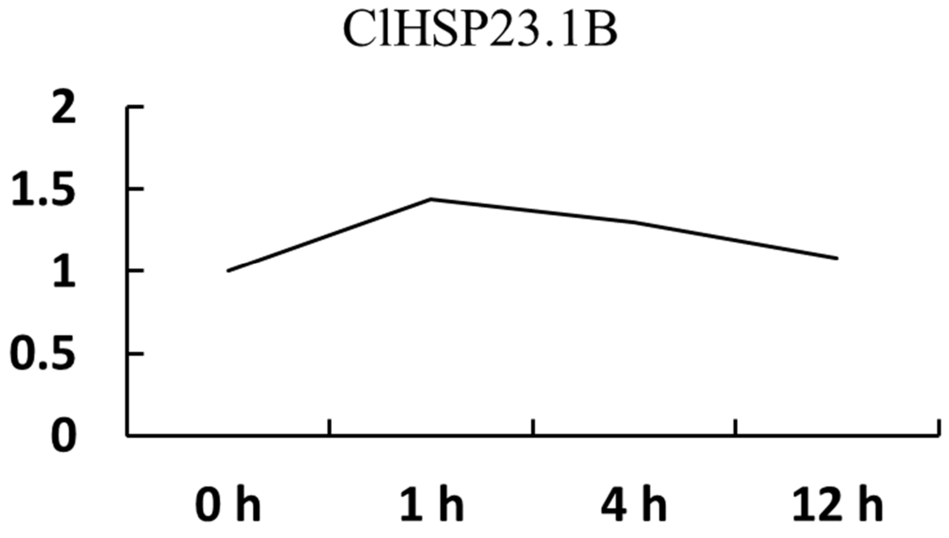

(C)

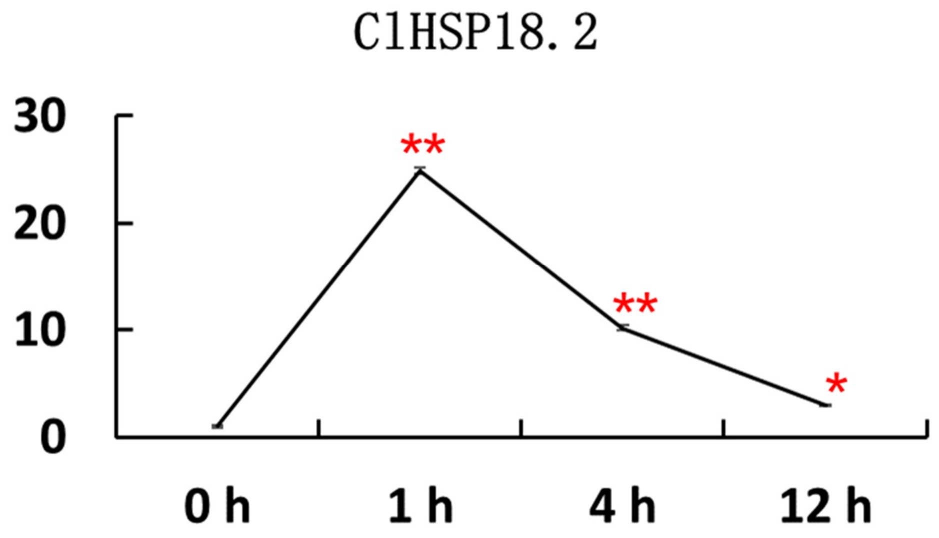

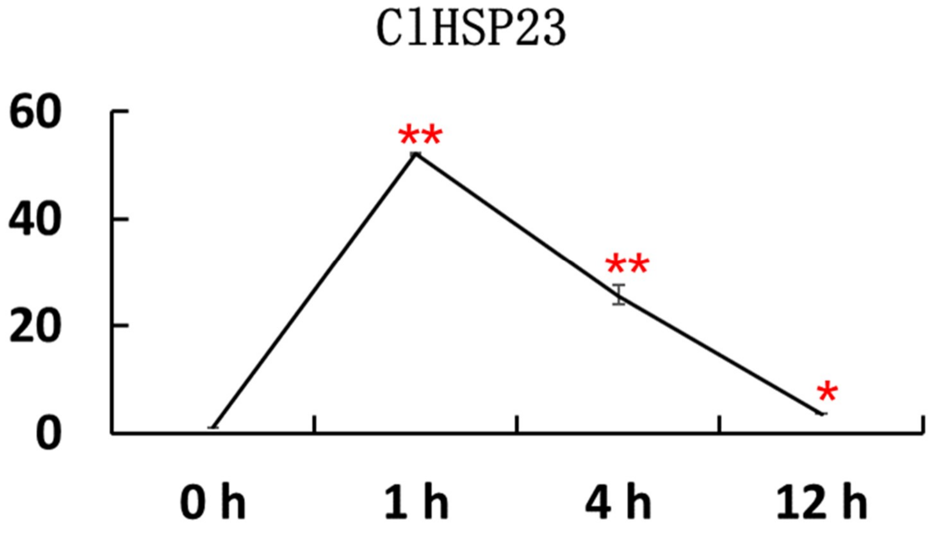
ClHSP23
60
40
20
0
**
**
*
0 h
1 h
4 h
12 h

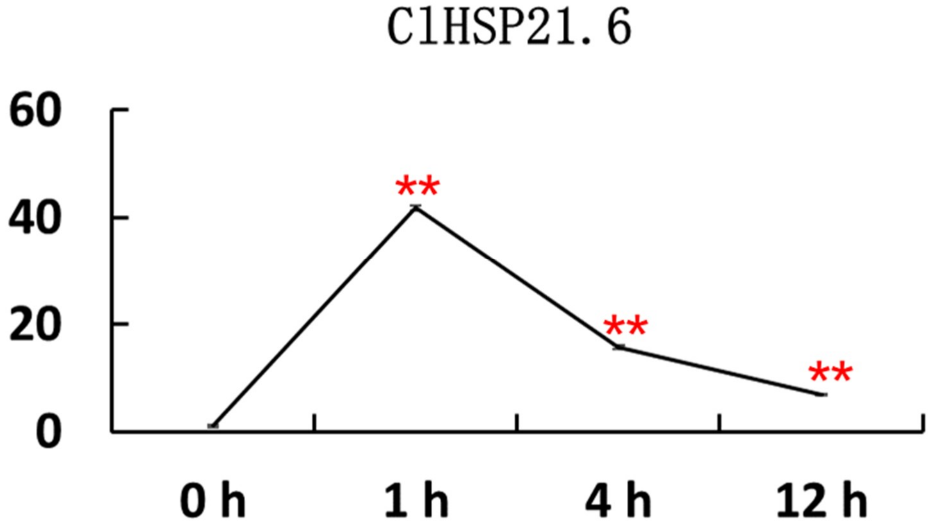
ClHSP21.6
60
40
20
0
**
**
**
0 h
1 h
4 h
12 h

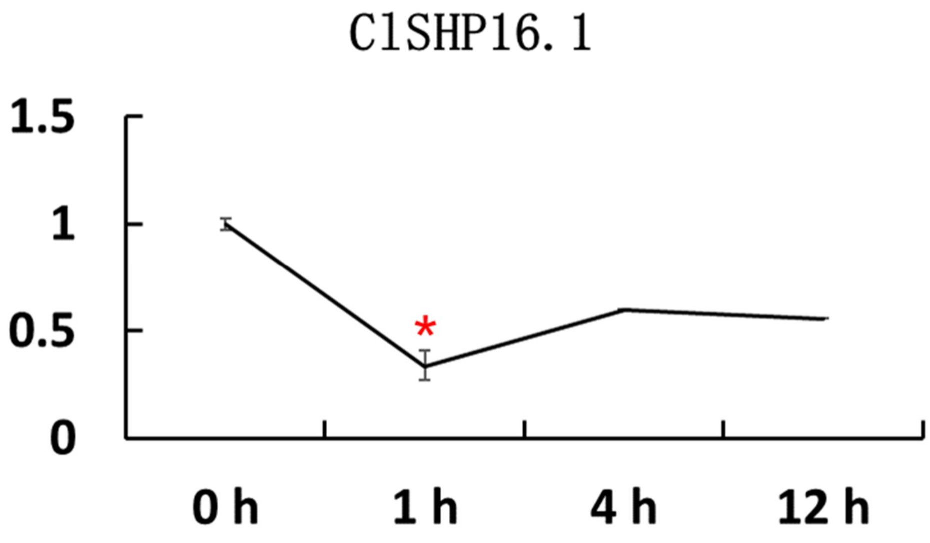
ClSHP16.1
1.5
1
0.5
0
*
0 h
1 h
4 h
12 h

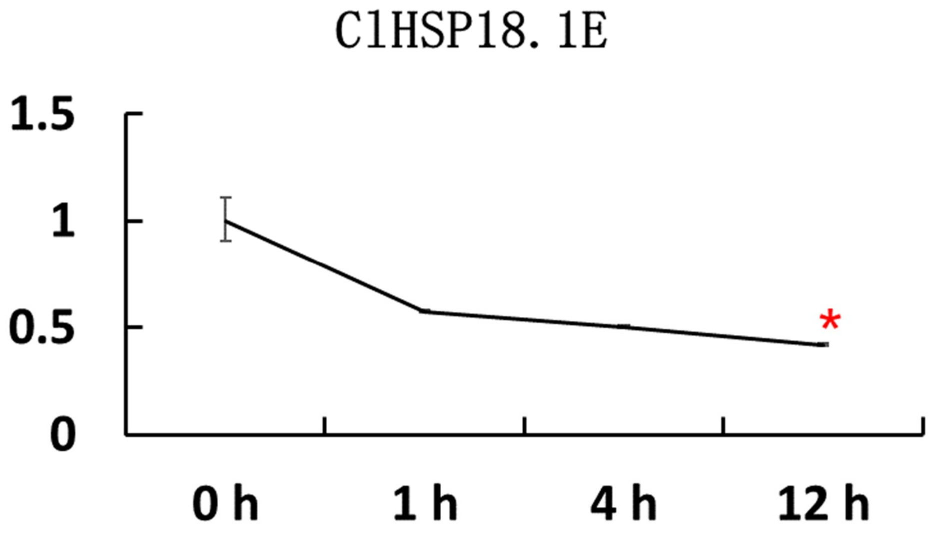
ClHSP18.1E
1.5
1
0.5
0
*
0 h
1 h
4 h
12 h

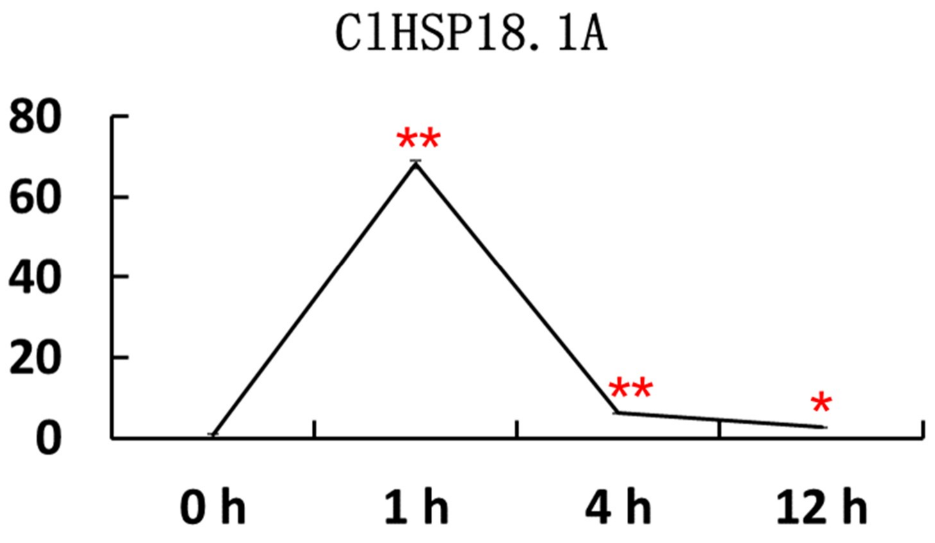

ClHSP18.1A
80
60
40
20
0
**
**
*
0 h
1 h
4 h
12 h

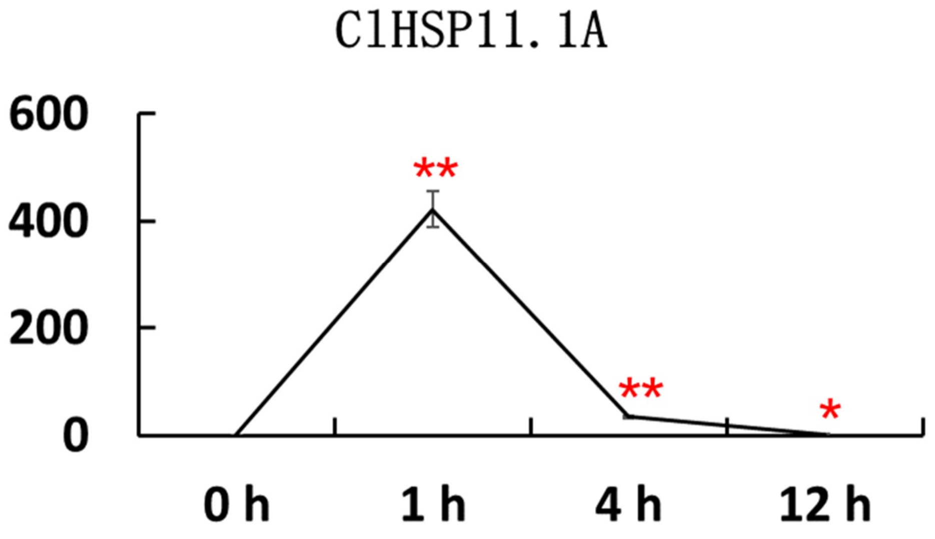

ClHSP11.1A
600
400
200
0
**
**
*
0 h
1 h
4 h
12 h

ClHSP17.6A

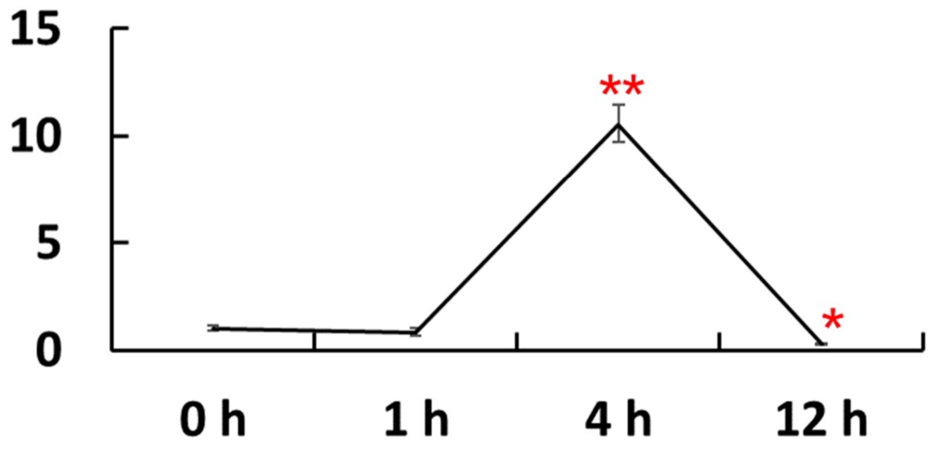

ClHSP17.6B

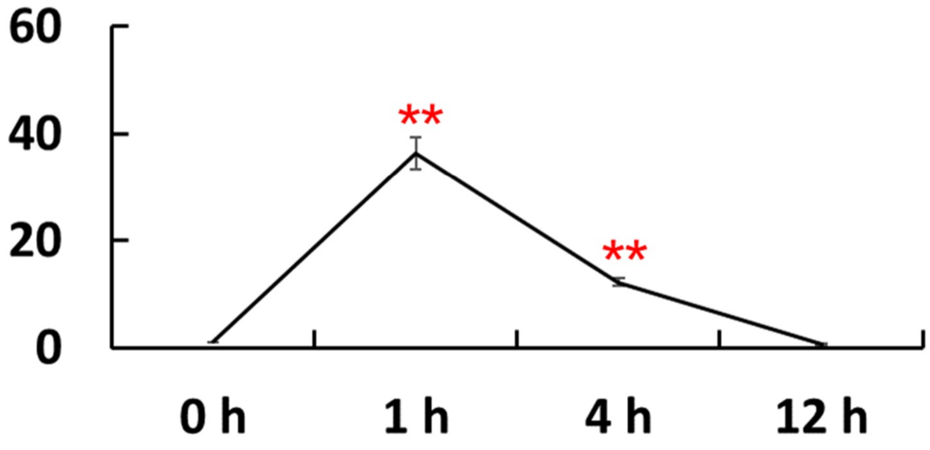

ClHSP27.9

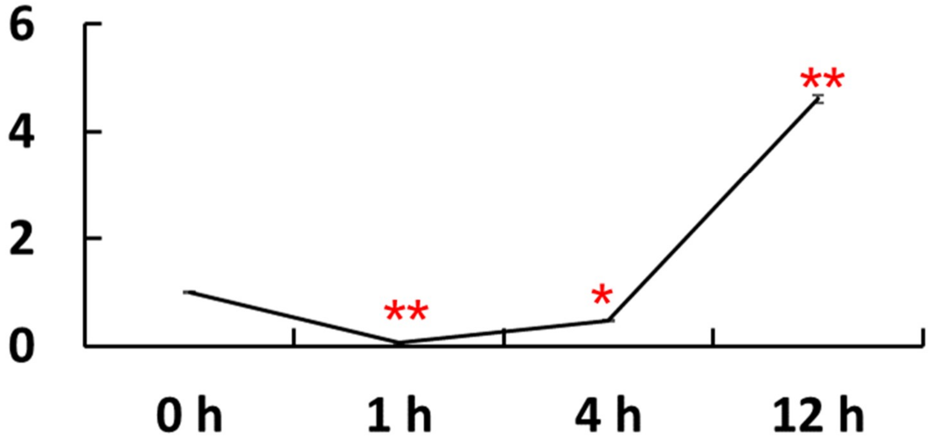

ClHSP39.8

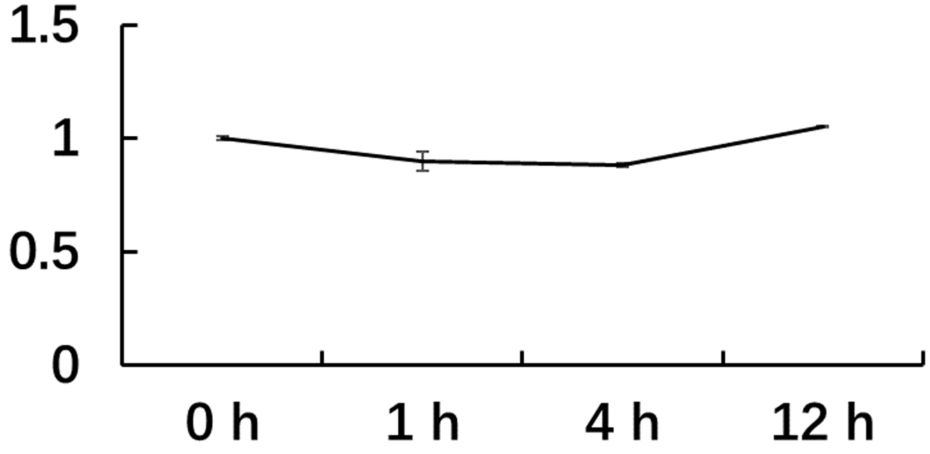

ClSHP42.6

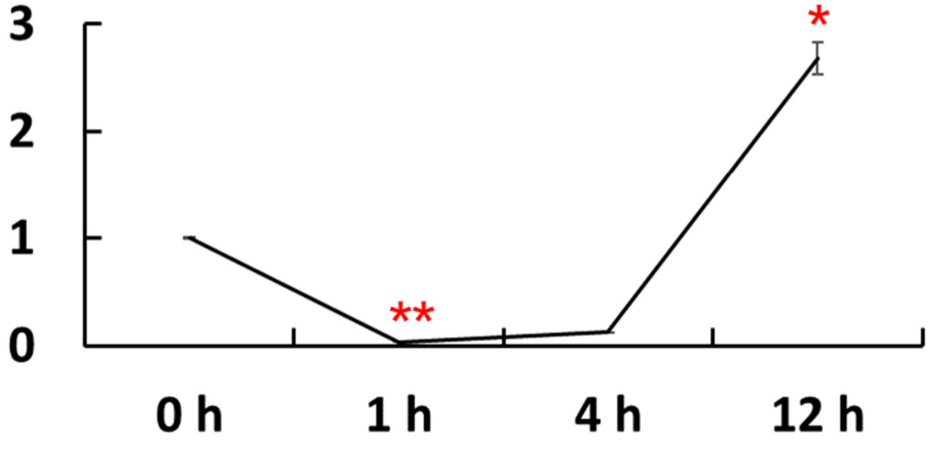

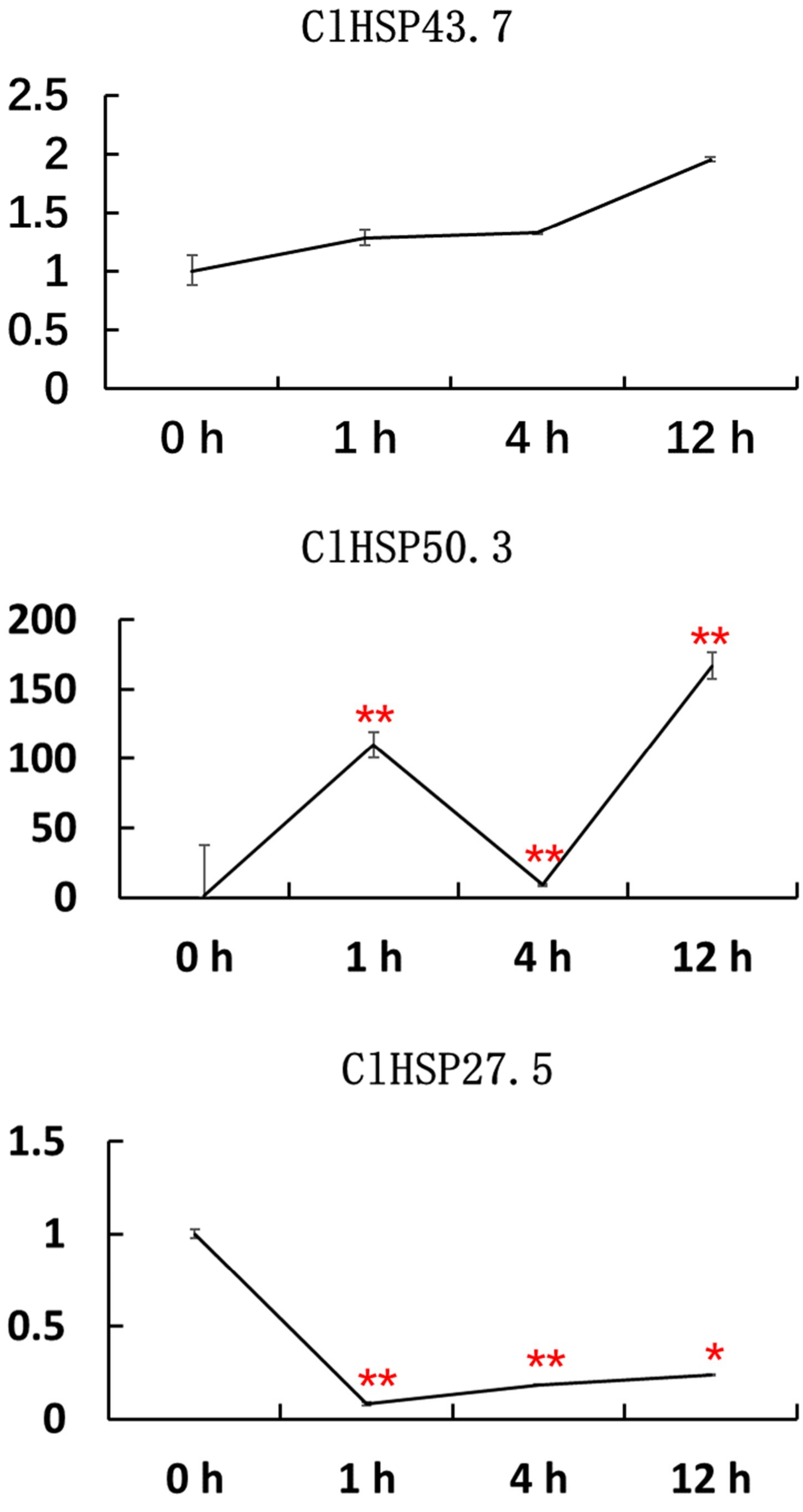
ClHSP43.7
2.5
2
1.5
1
0.5
0
0 h
1 h
4 h
12 h
ClHSP50.3
200
150
100
50
0
**
**
**
0 h
1 h
4 h
12 h
ClHSP27.5
1.5
1
0.5
0
**
**
*
0 h
1 h
4 h
12 h

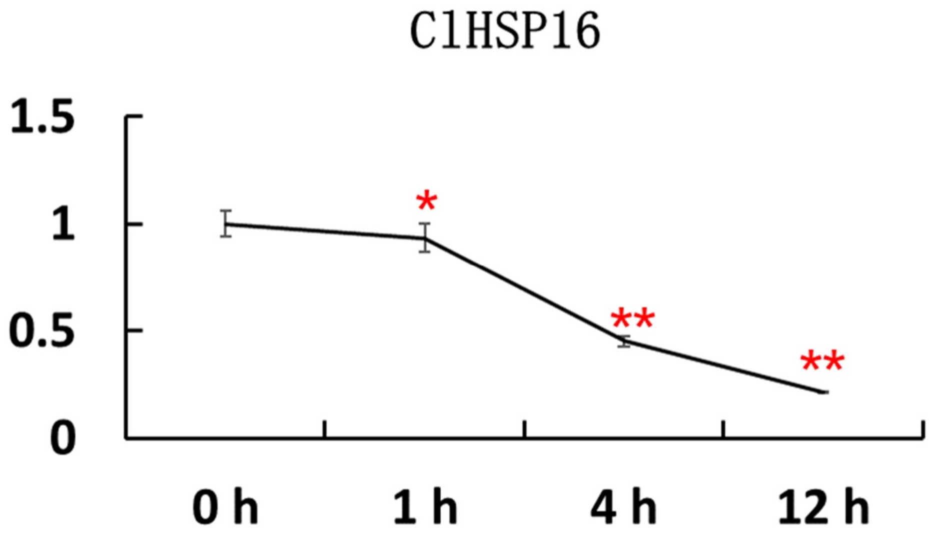
ClHSP16
1.5
1
0.5
0
*
**
**
0 h
1 h
4 h
12 h

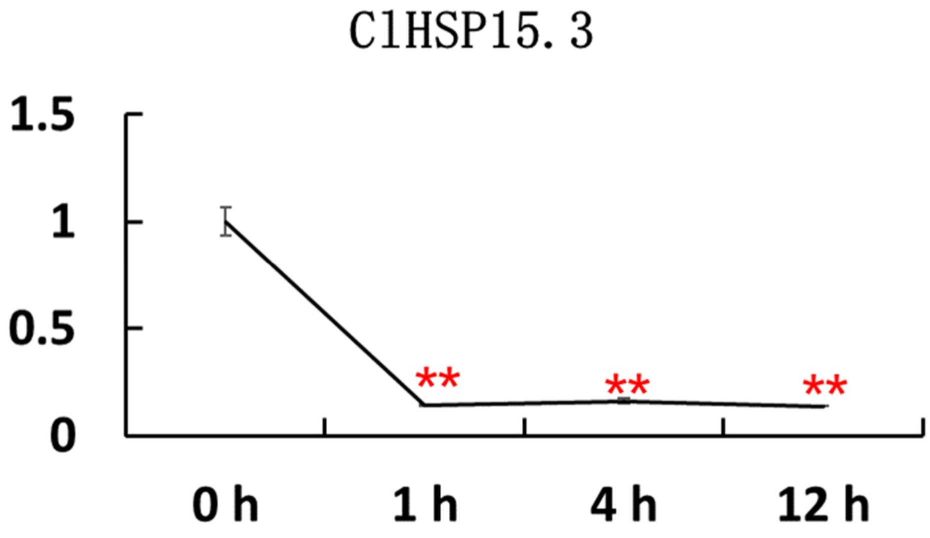
ClHSP15.3
1.5
1
0.5
0
**
**
**
0 h
1 h
4 h
12 h

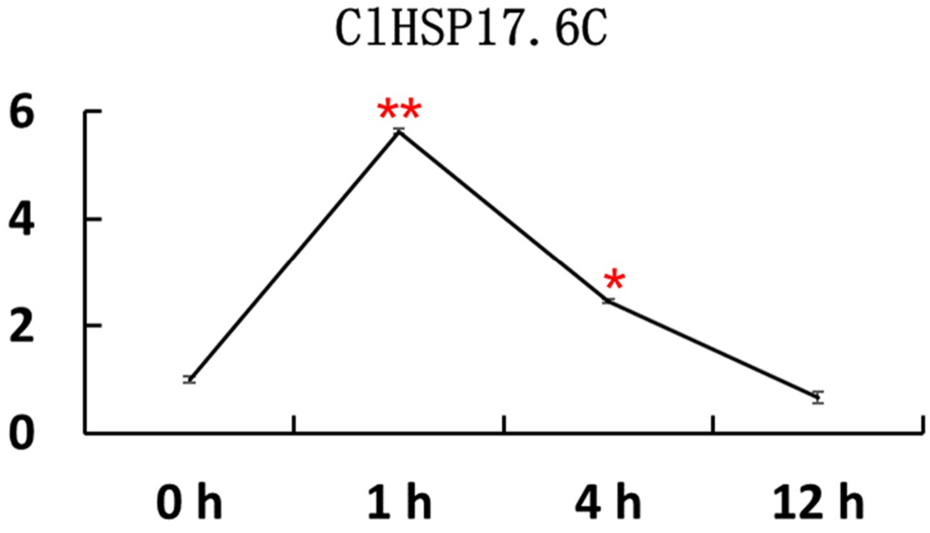
ClHSP17.6C
6
4
2
0
**
*
0 h
1 h
4 h
12 h

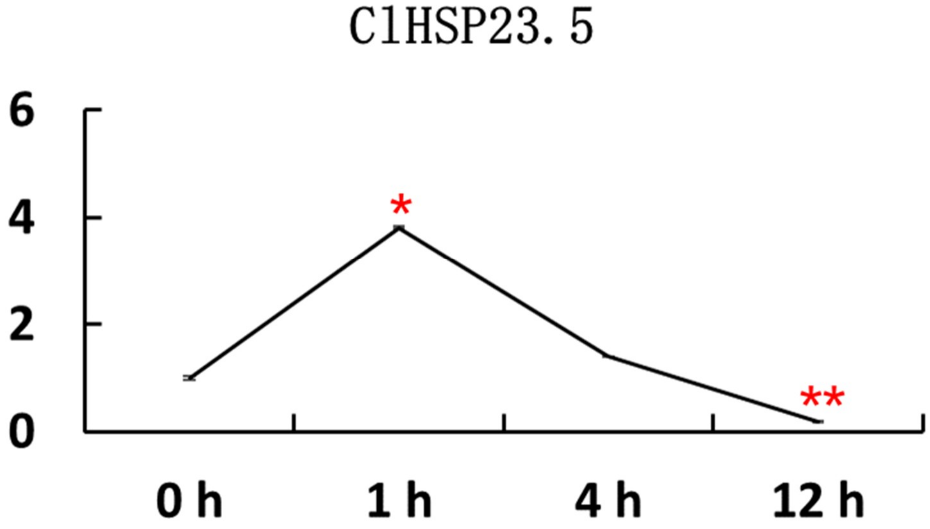
ClHSP23.5
6
4
2
0
*
**
0 h
1 h
4 h
12 h

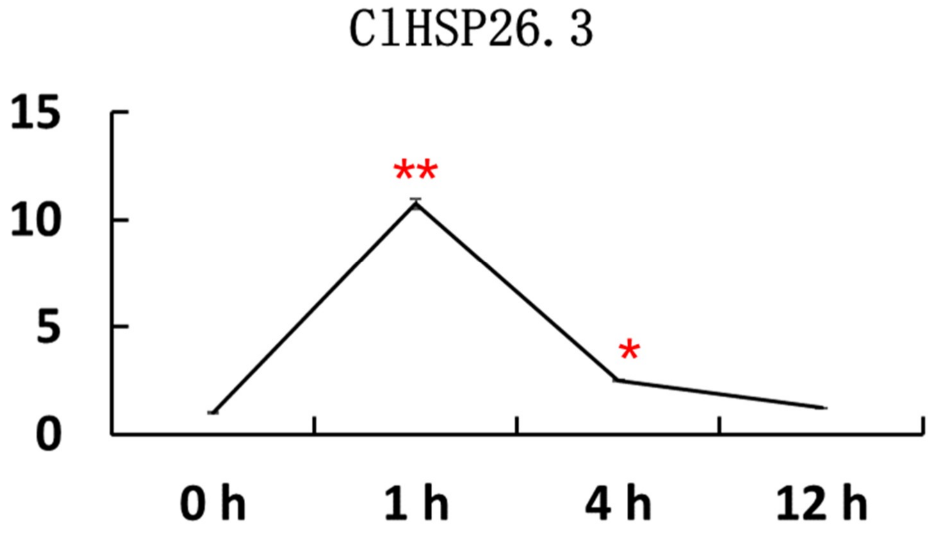
ClHSP26.3
15
10
5
0
**
*
0 h
1 h
4 h
12 h

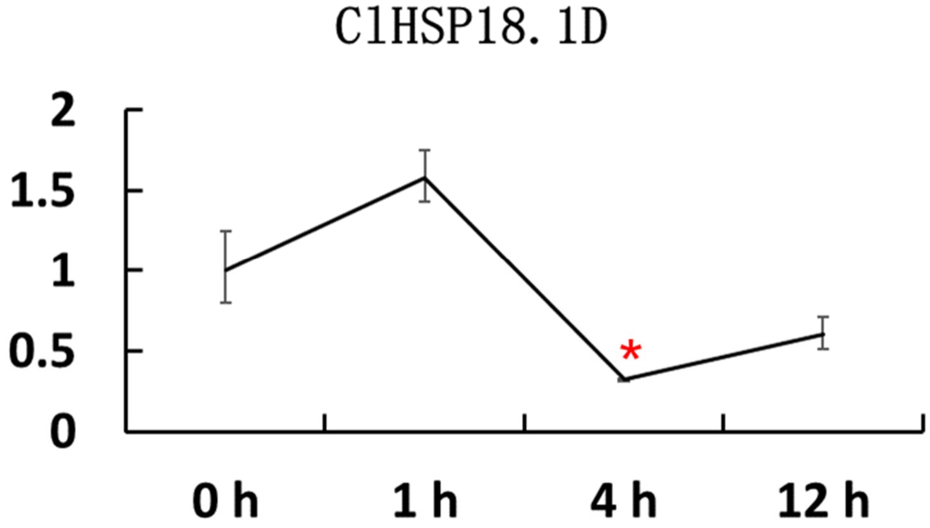

ClHSP18.1D
2
1.5
1
0.5
0
*
0 h
1 h
4 h
12 h

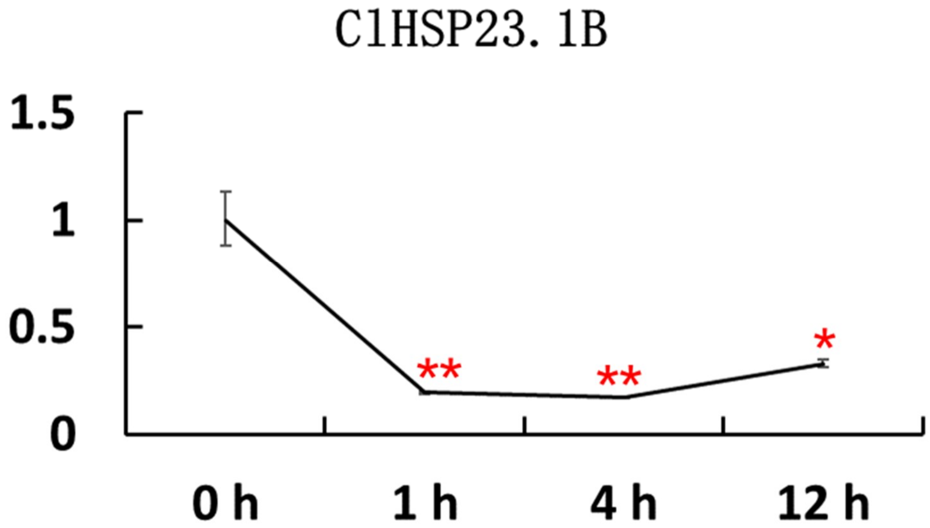

ClHSP23.1B
1.5
1
0.5
0
**
**
*
0 h
1 h
4 h
12 h

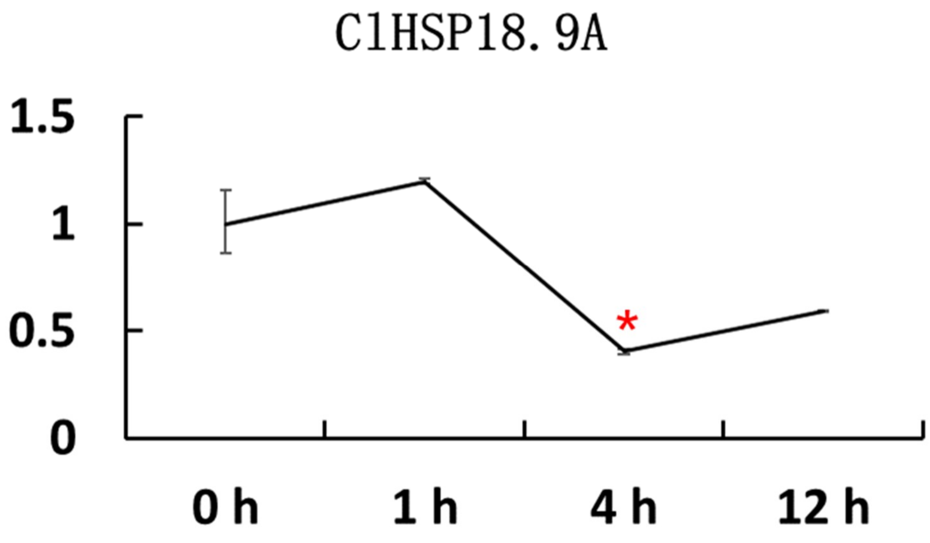
ClHSP18.9A
1.5
1
0.5
0
*
0 h
1 h
4 h
12 h

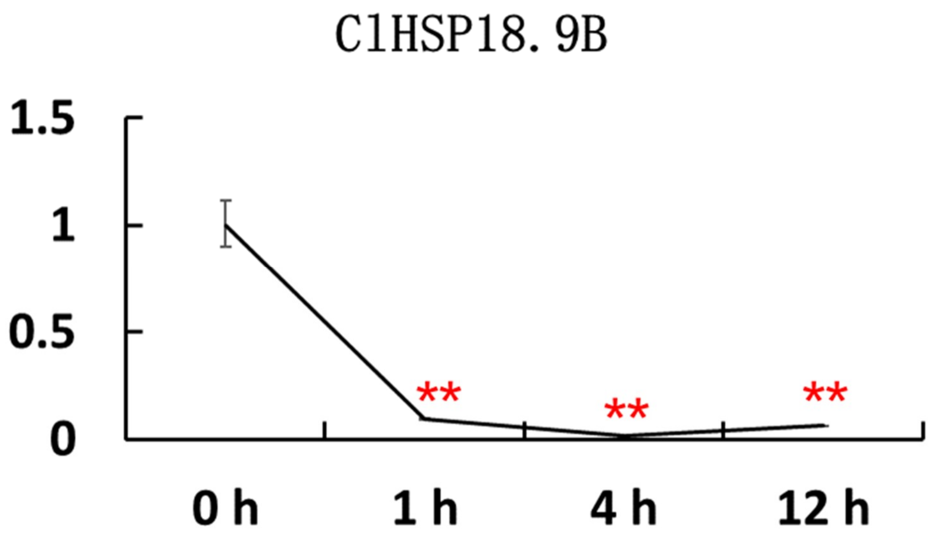
ClHSP18.9B
1.5
1
0.5
0
**
**
**
0 h
1 h
4 h
12 h

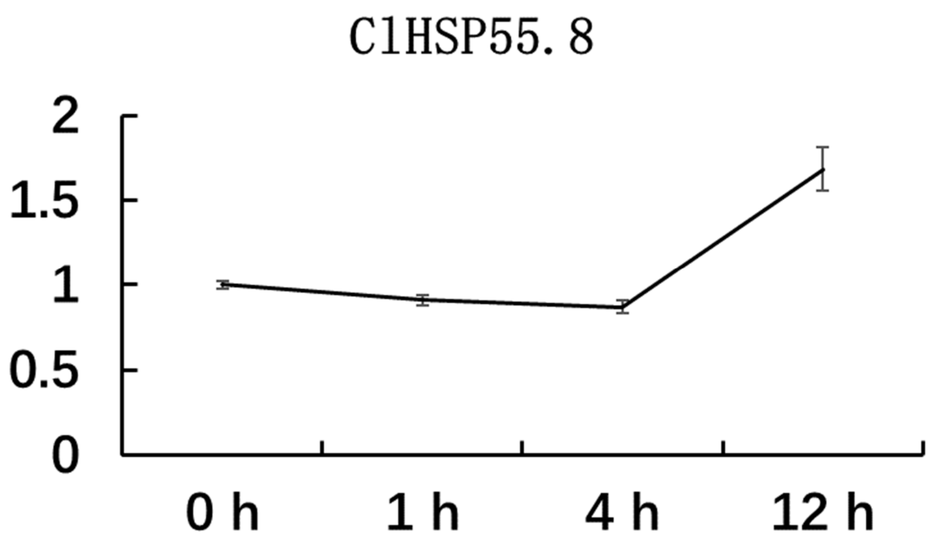
ClHSP55.8
2
1.5
1
0.5
0
0 h
1 h
4 h
12 h

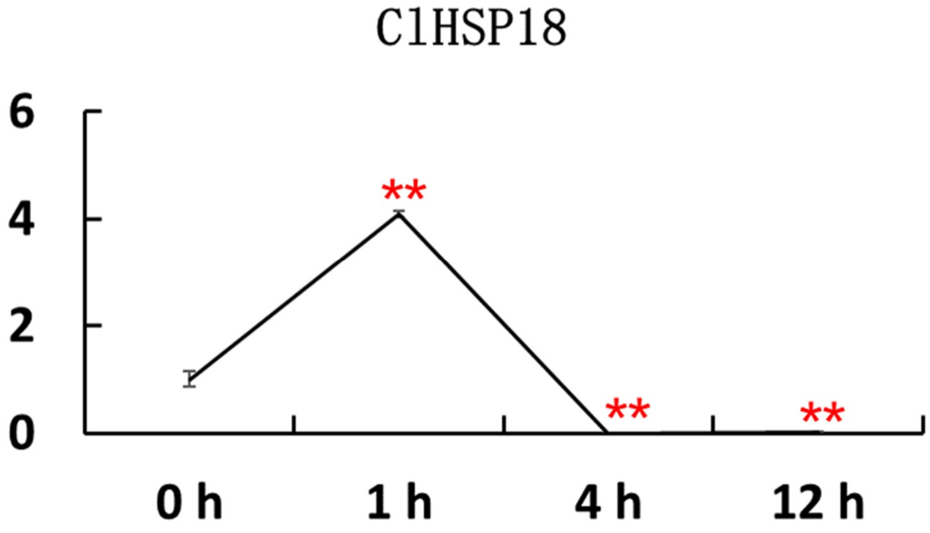
ClHSP18
6
4
2
0
**
**
**
0 h
1 h
4 h
12 h

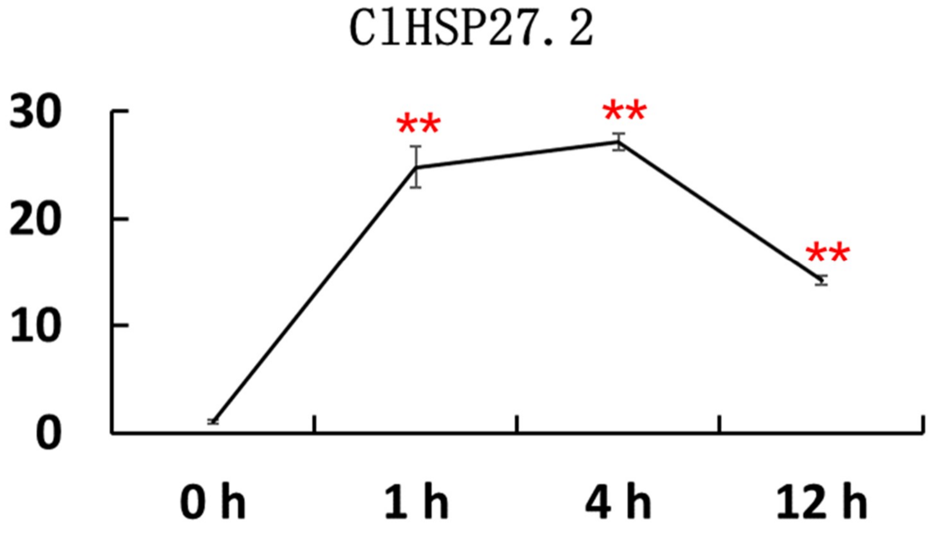
ClHSP27.2
30
20
10
0
**
**
**
0 h
1 h
4 h
12 h

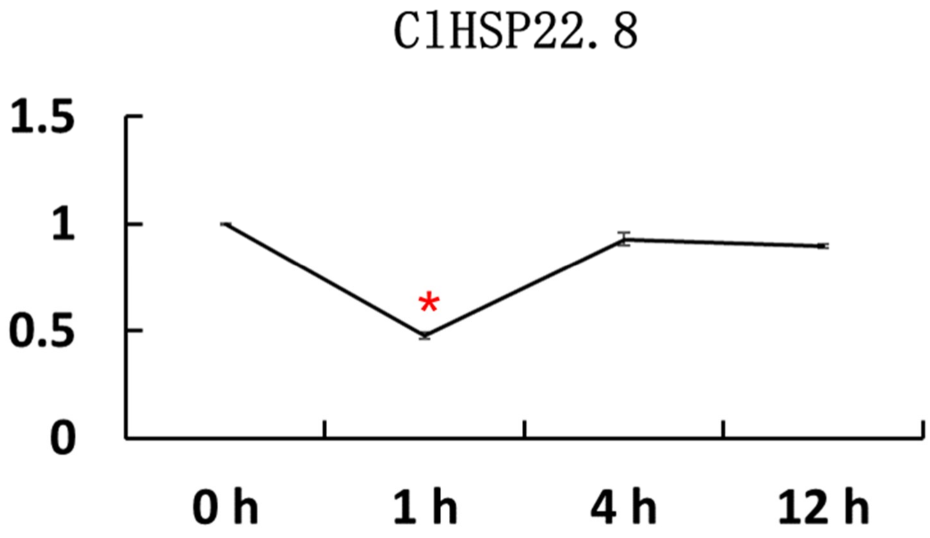
ClHSP22.8
1.5
1
0.5
0
*
0 h
1 h
4 h
12 h

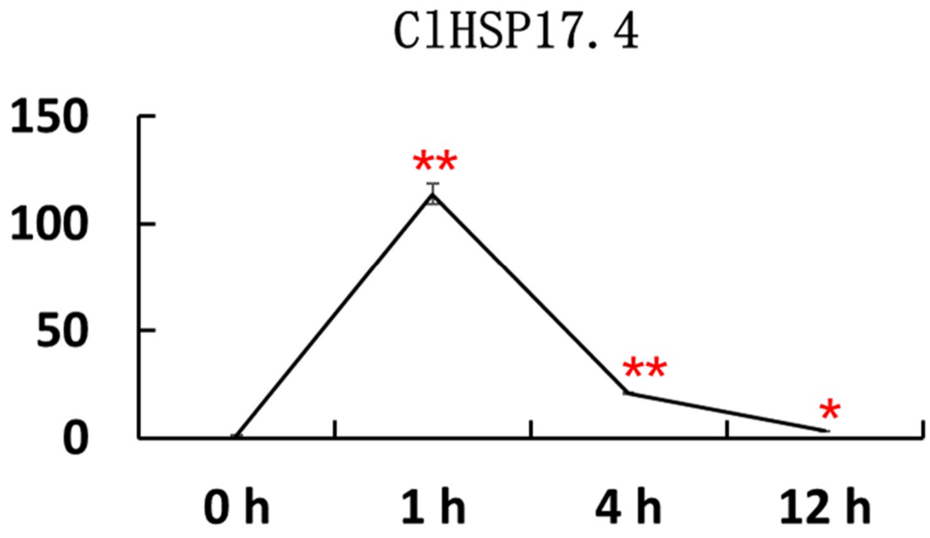
ClHSP17.4
150
100
50
0
**
**
*
0 h
1 h
4 h
12 h

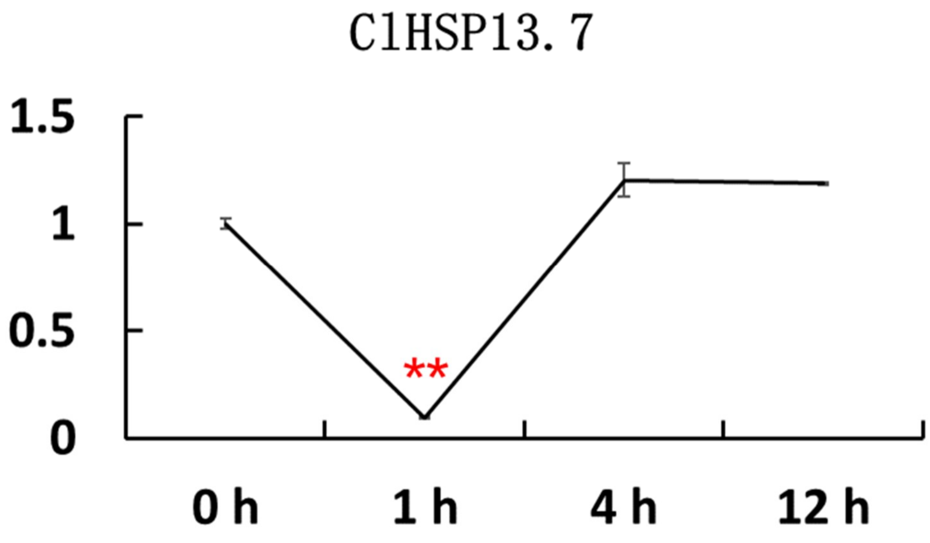

(D)

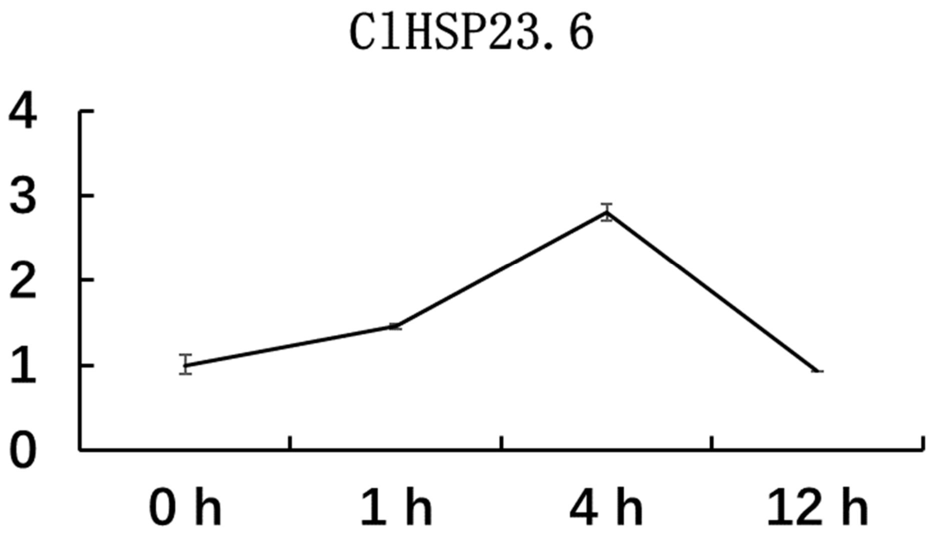

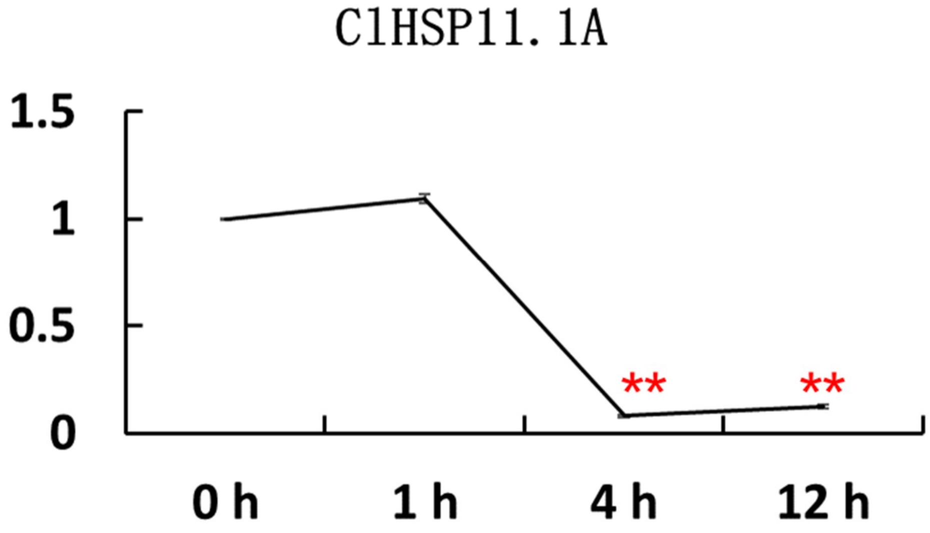
ClHSP11.1A
1.5
1
0.5
0
**
**
0 h
1 h
4 h
12 h

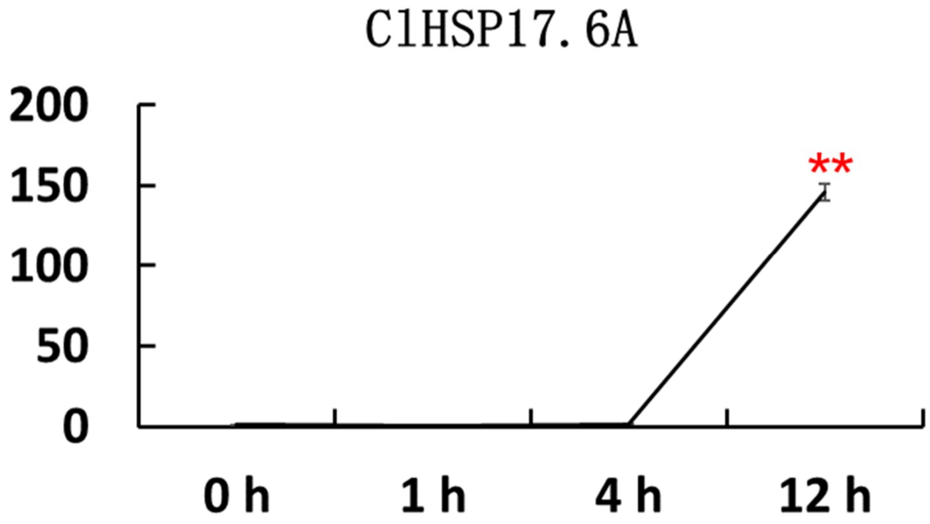
ClHSP17.6A
200
150
100
50
0
**
0 h
1 h
4 h
12 h

ClHSP17.6B

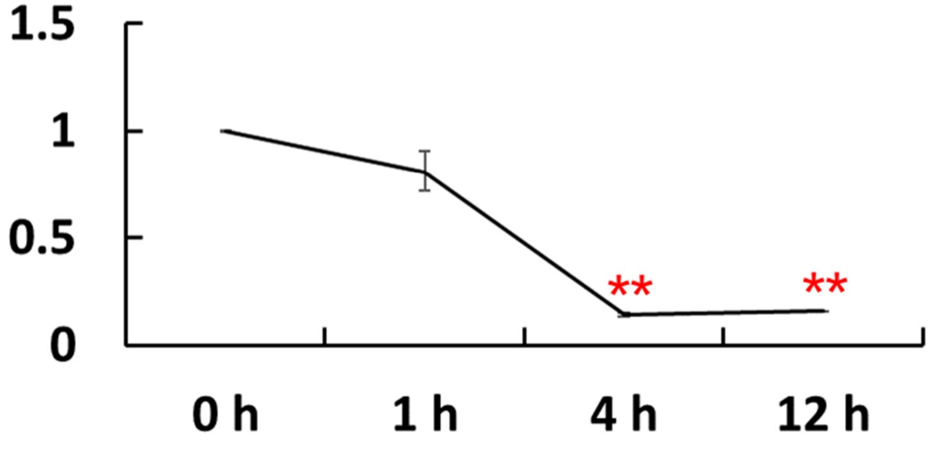

ClHSP18.1D

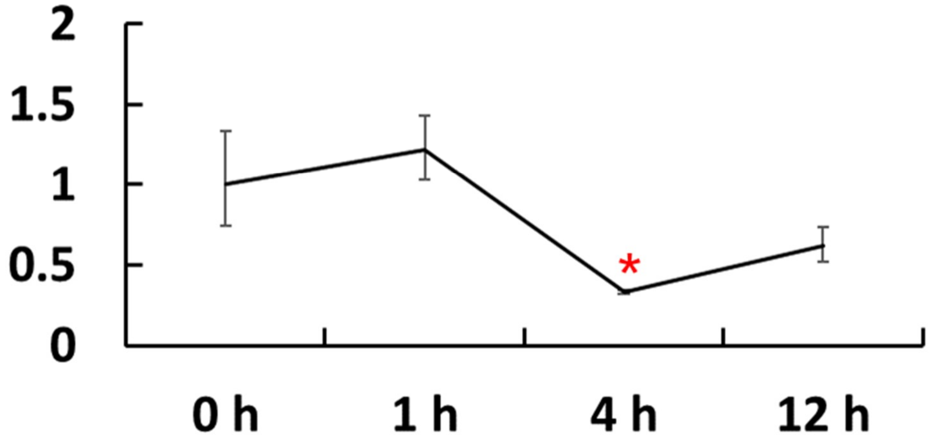

ClHSP18.1A

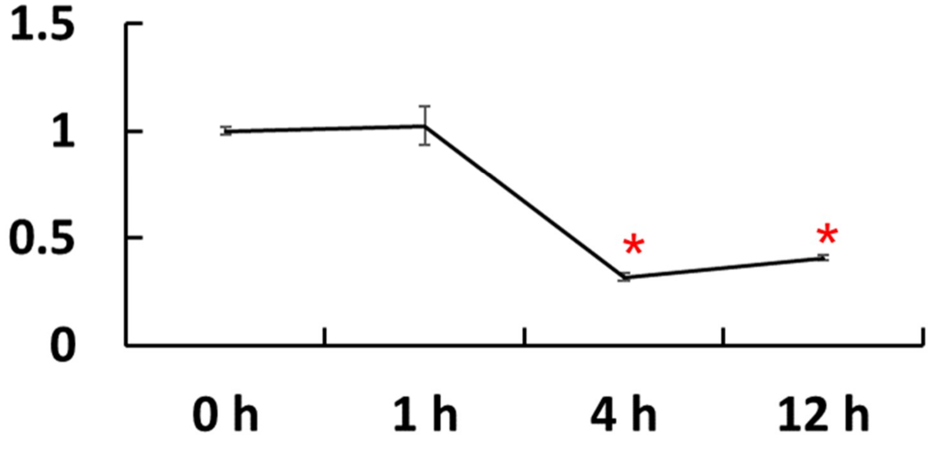

ClHSP26.3

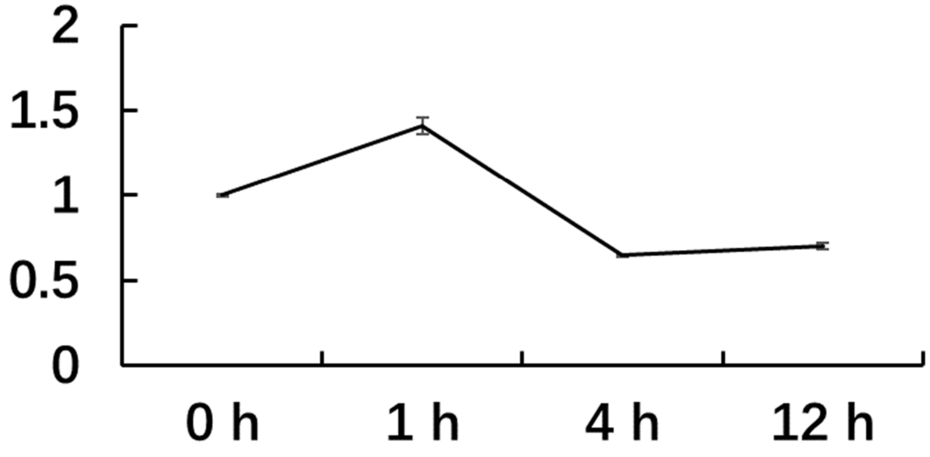

ClHSP38.8

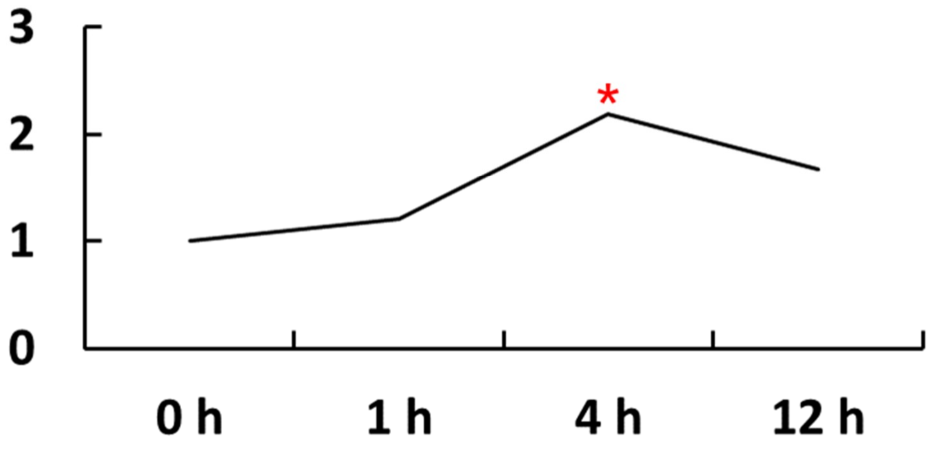

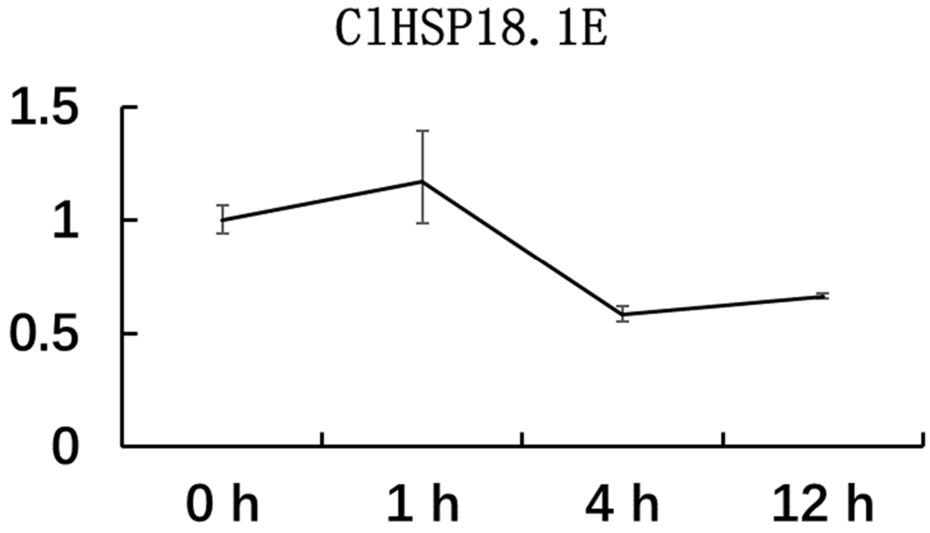
ClHSP18.1E
1.5
1
0.5
0
0 h
1 h
4 h
12 h

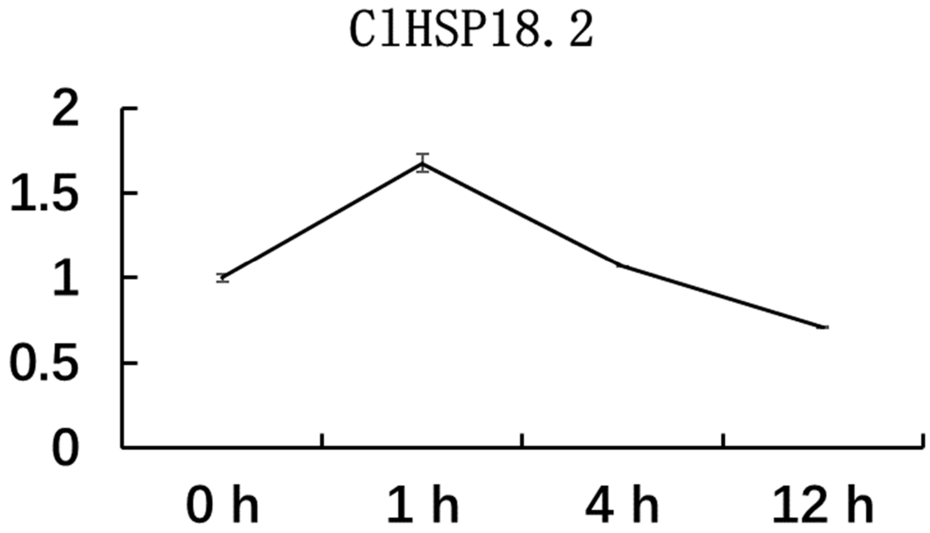
ClHSP18.2
2
1.5
1
0.5
0
0 h
1 h
4 h
12 h

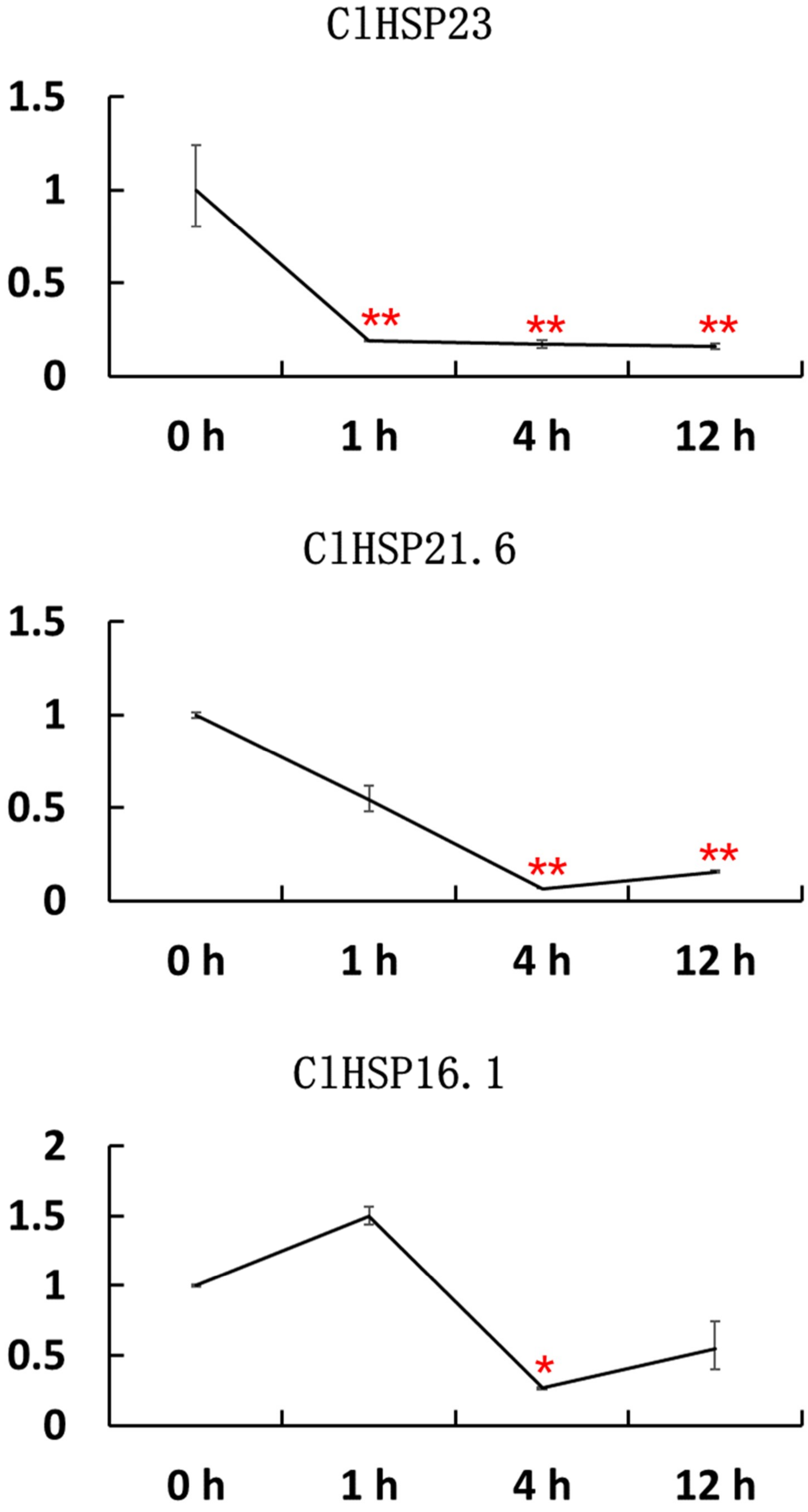
ClHSP23
1.5
1
0.5
0
**
**
**
0 h
1 h
4 h
12 h
ClHSP21.6
1.5
1
0.5
0
**
**
0 h
1 h
4 h
12 h
ClHSP16.1
2
1.5
1
0.5
0
*
0 h
1 h
4 h
12 h

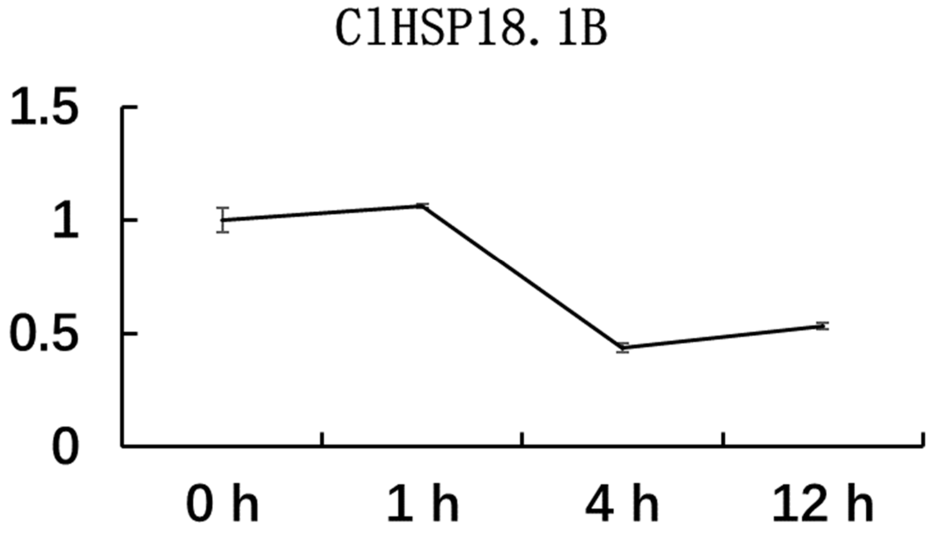
ClHSP18.1B
1.5
1
0.5
0
0 h
1 h
4 h
12 h

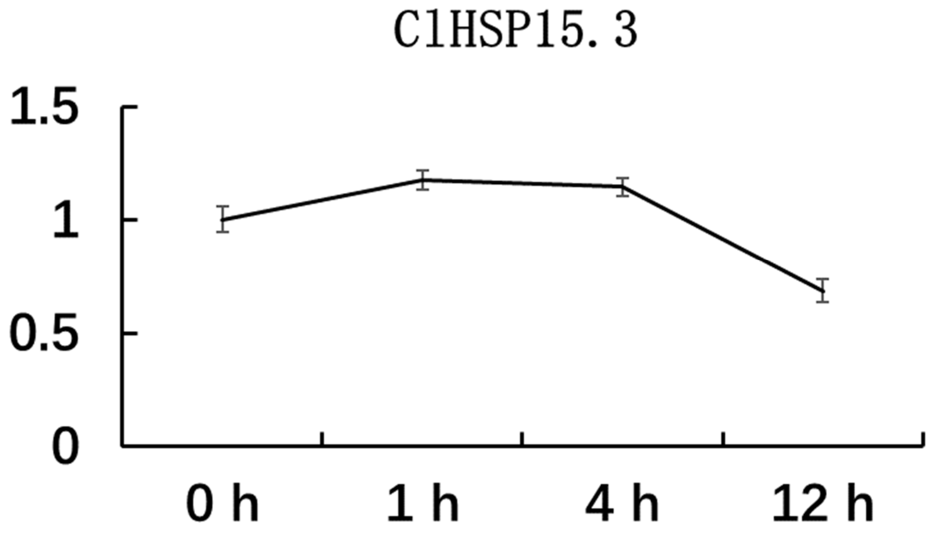
ClHSP15.3
1.5
1
0.5
0
0 h
1 h
4 h
12 h

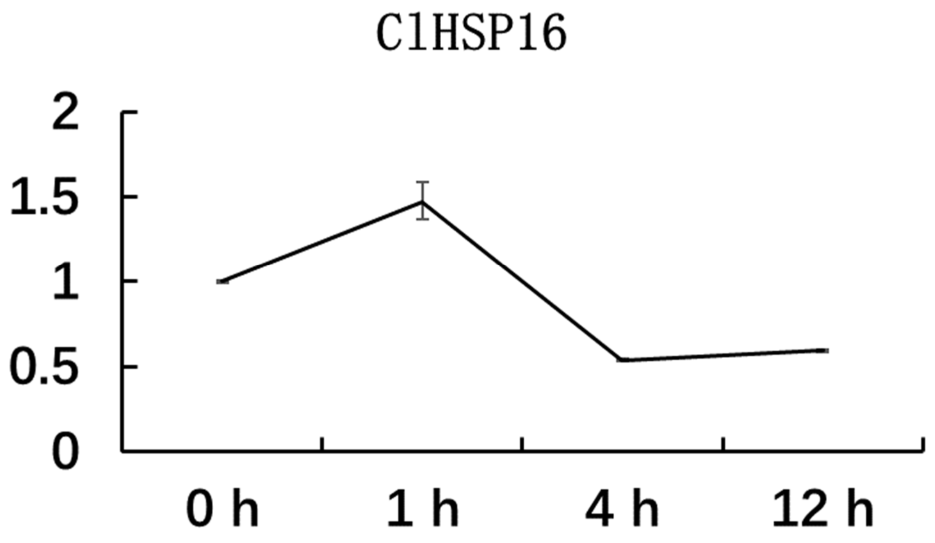
ClHSP16
2
1.5
1
0.5
0
0 h
1 h
4 h
12 h

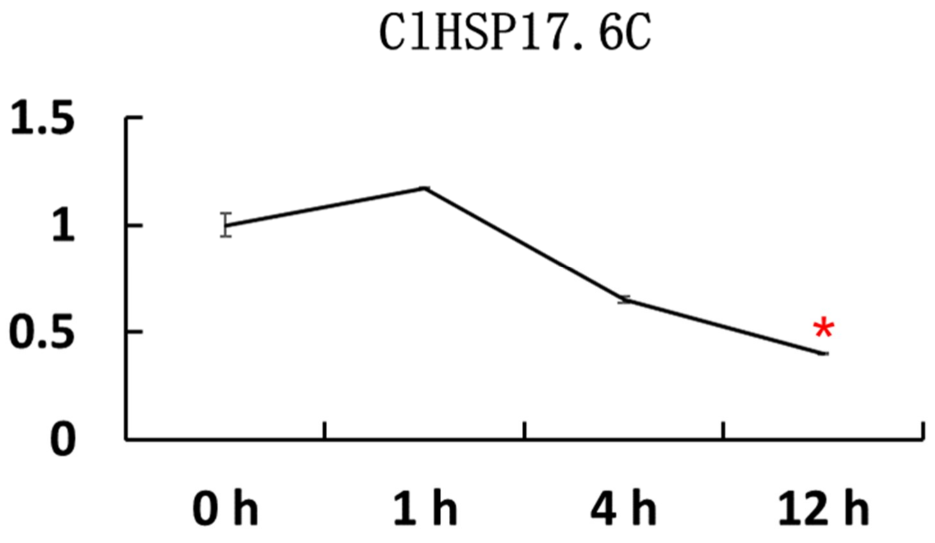
ClHSP17.6C
1.5
1
0.5
0
*
0 h
1 h
4 h
12 h

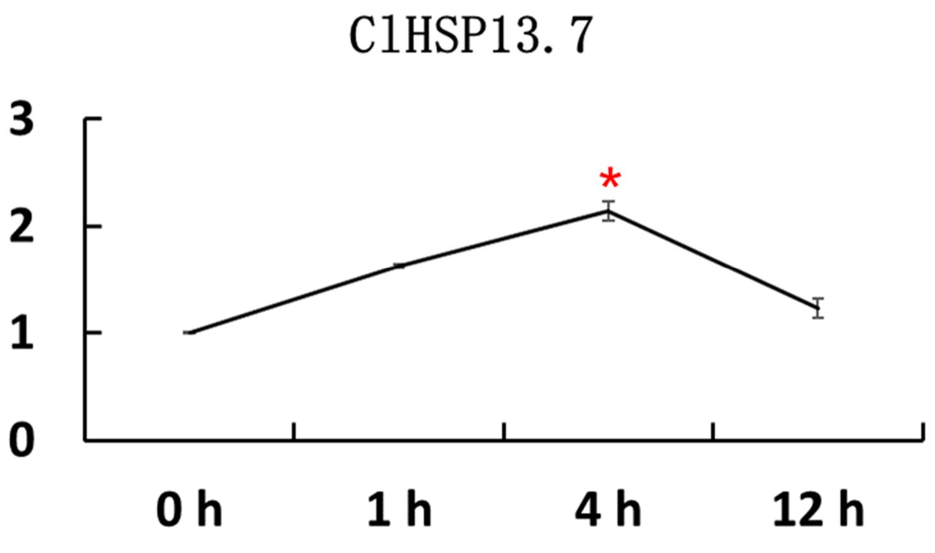
ClHSP13.7
3
2
1
0
*
0 h
1 h
4 h
12 h

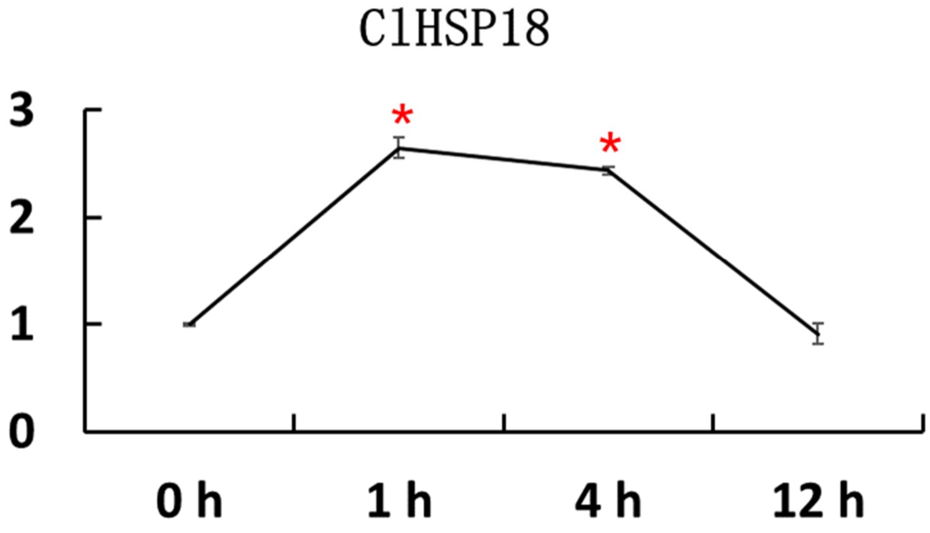
ClHSP18
3
2
1
0
*
*
0 h
1 h
4 h
12 h

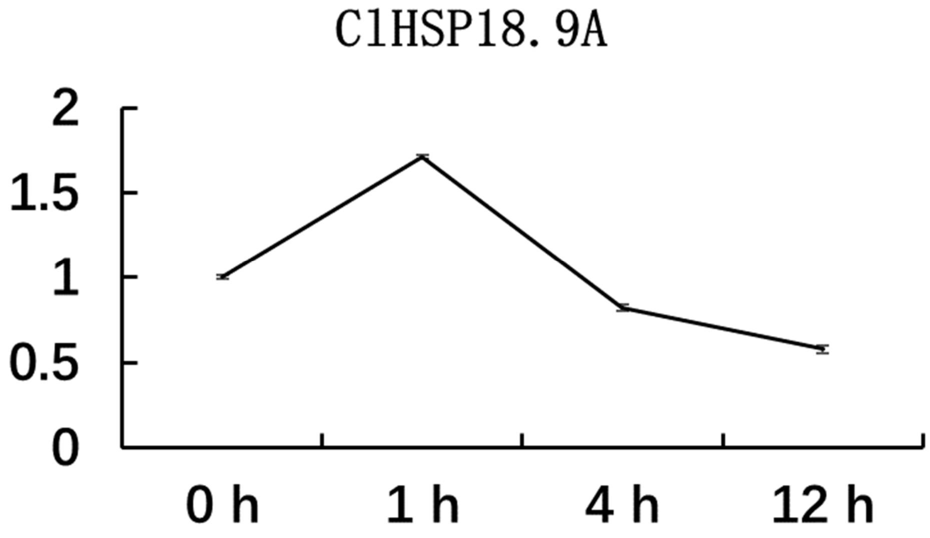
ClHSP18.9A
2
1.5
1
0.5
0
0 h
1 h
4 h
12 h

ClHSP18.9B

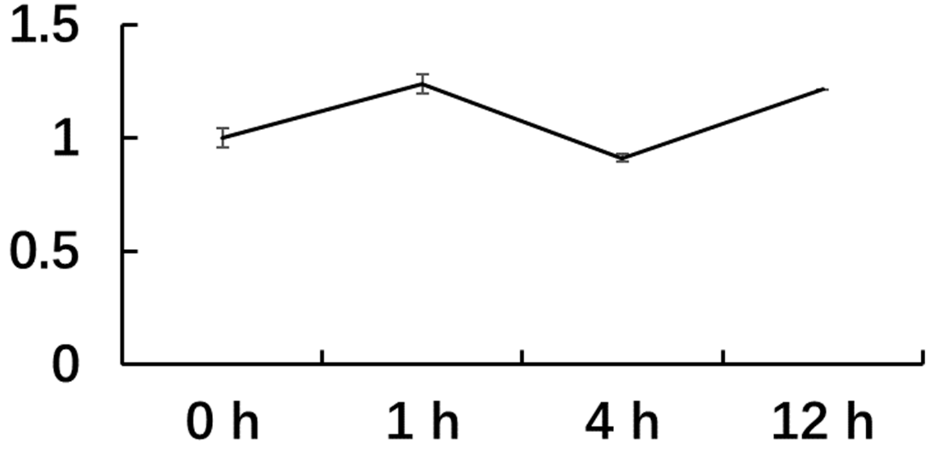

ClHSP21.8

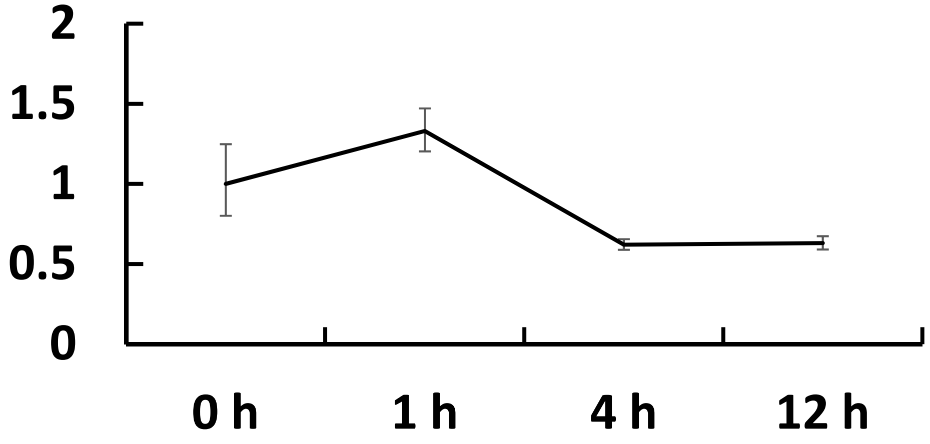

ClHSP23.1B

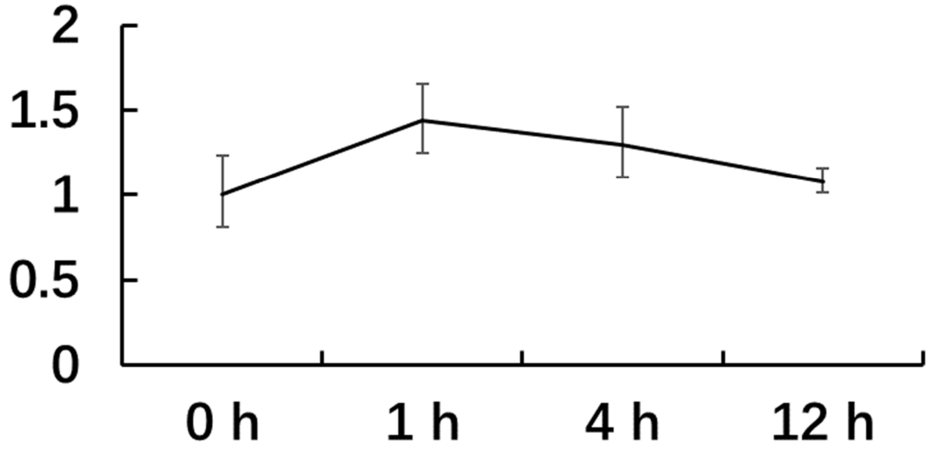

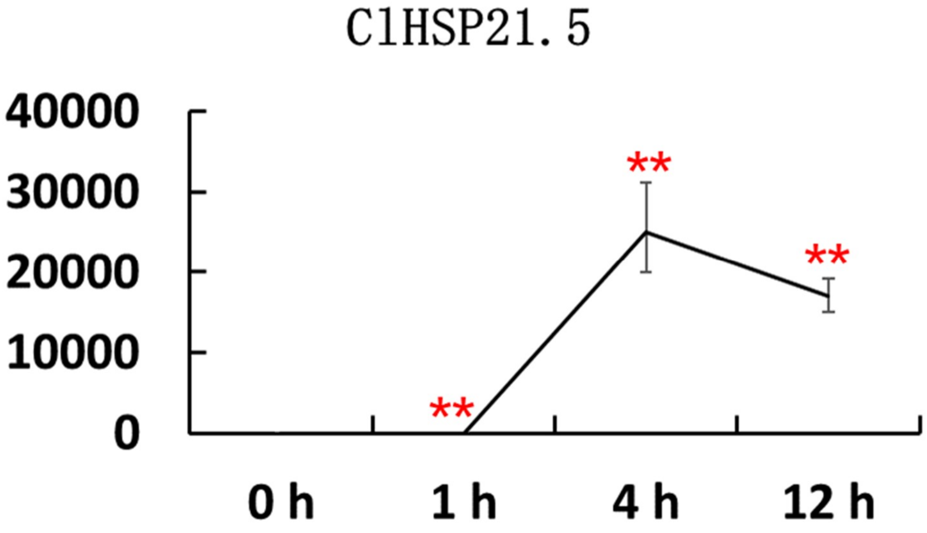
ClHSP21.5
40000
30000
20000
10000
0
**
**
**
0 h
1 h
4 h
12 h

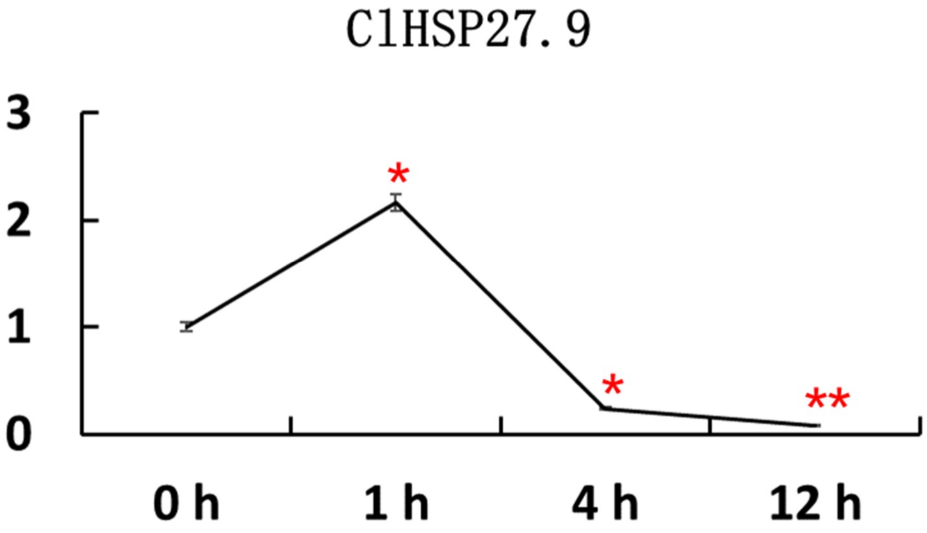
ClHSP27.9
3
2
1
0
*
*
**
0 h
1 h
4 h
12 h

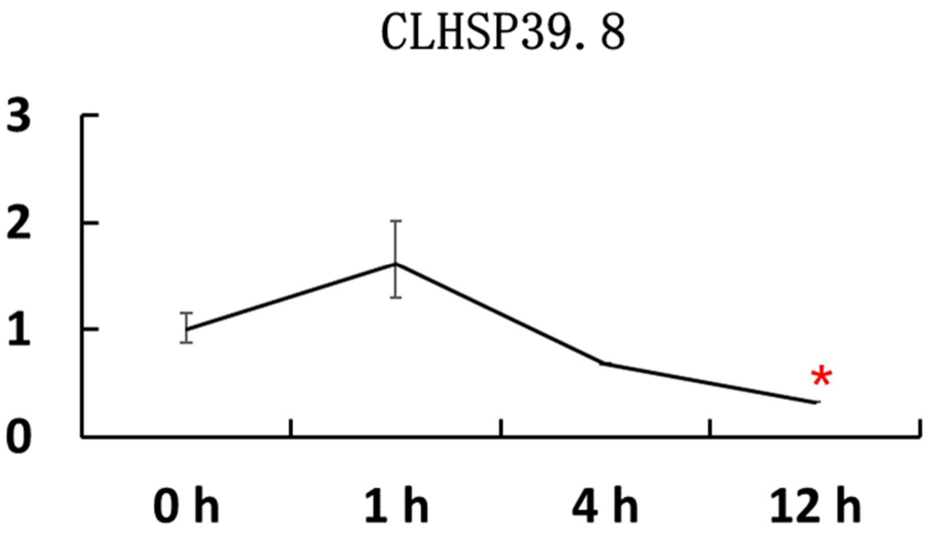
CLHSP39.8
3
2
1
0
*
0 h
1 h
4 h
12 h

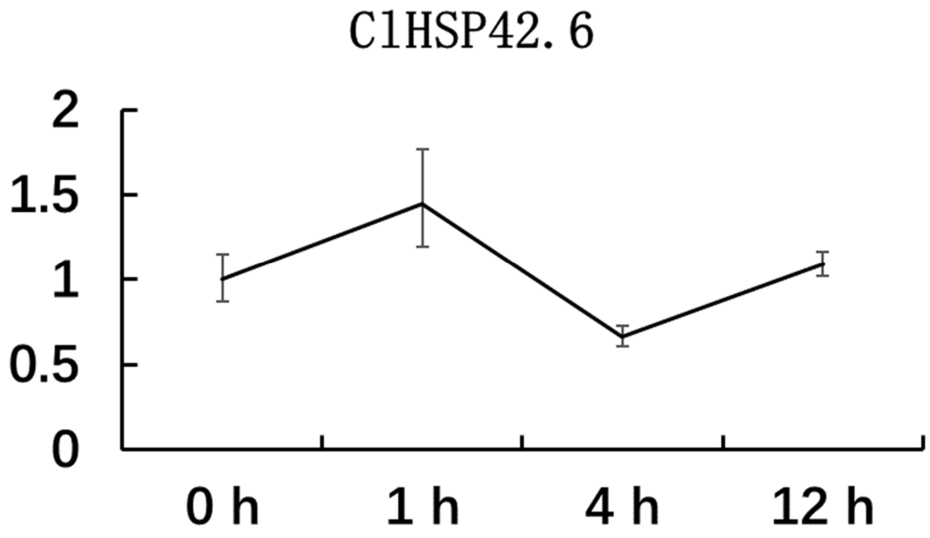
ClHSP42.6
2
1.5
1
0.5
0
0 h
1 h
4 h
12 h

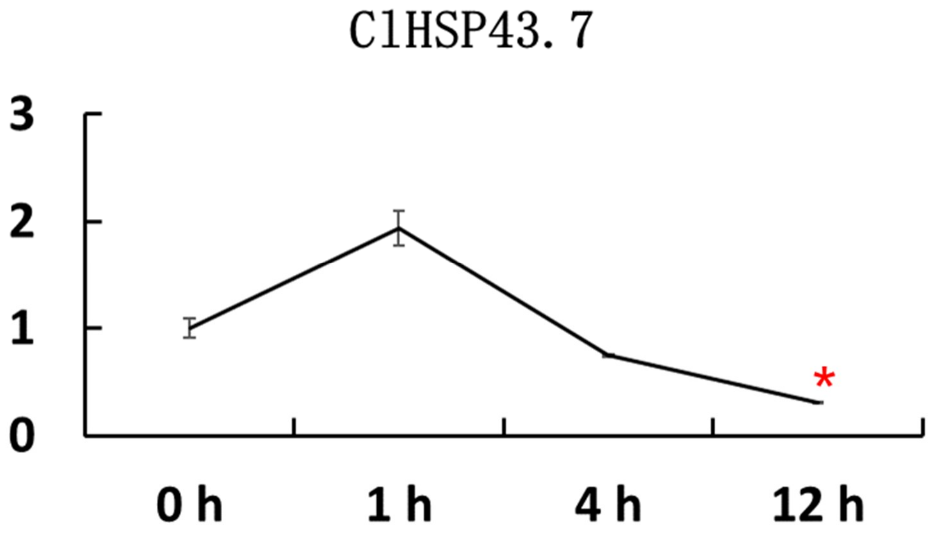
ClHSP43.7
3
2
1
0
*
0 h
1 h
4 h
12 h

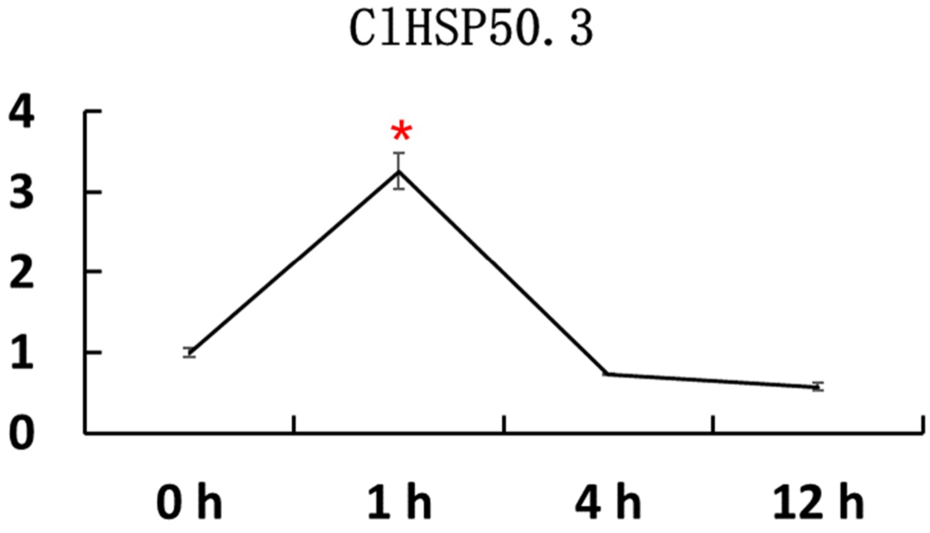
ClHSP50.3
4
3
2
1
0
*
0 h
1 h
4 h
12 h
