# Supplementary figures and images for: Genome-Wide Analysis of Watermelon HSP20s and Their Expression Profiles and Subcellular Locations under Stresses
Source: Int J Mol Sci. 2018 Dec 20;20(1):12. doi: 10.3390/ijms20010012 (PMC6337729; doi:10.3390/ijms20010012)

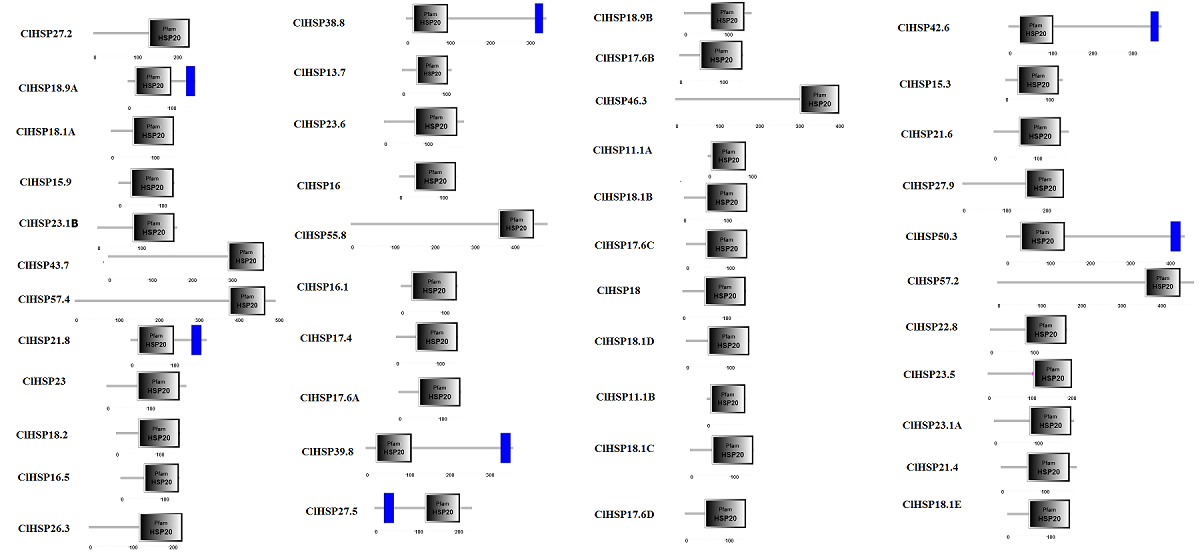

Supplement: Supplementary file 1 [file ijms-20-00012-s001.zip › Figure S1. domain structure of ClHSP20.tif]

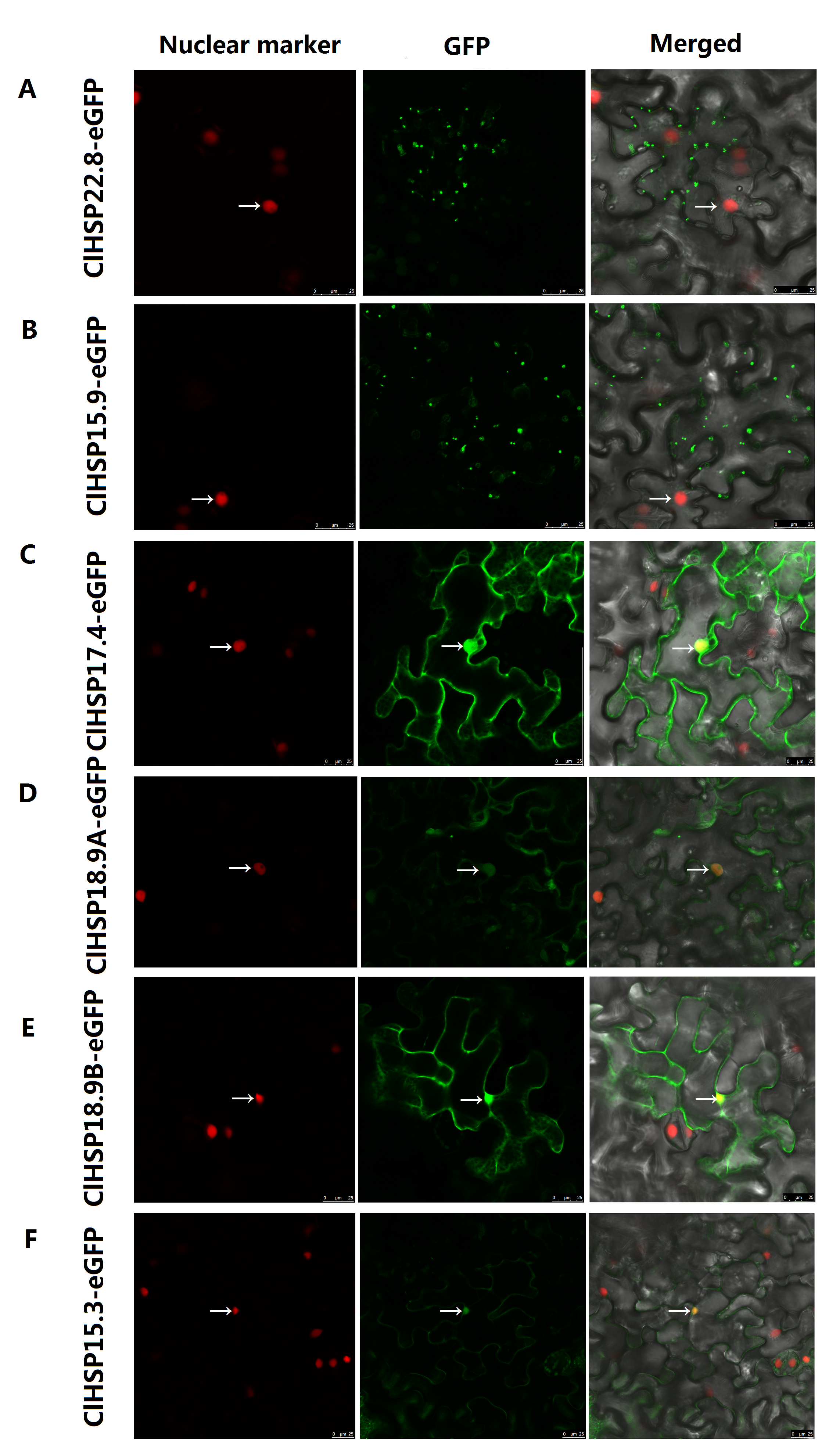

Supplement: Supplementary file 1 [file ijms-20-00012-s001.zip › Figure S2 Sublocation of ClHSP20 .tif]
